# Supplementary material for: Periprosthetic fractures: the next fragility fracture epidemic? A national observational study
Source: BMJ Open. 2020 Dec 10;10(12):e042371. doi: 10.1136/bmjopen-2020-042371 (PMC7733197; doi:10.1136/bmjopen-2020-042371)
Supplement: Supplementary data [file bmjopen-2020-042371supp001.pdf]

Supplementary Data - Periprosthetic fractures – the next fragility fracture epidemic? A national observational study

Supplementary Table 1. The 20 most commonly performed procedures by four-character OPCS code.

Op code	Description	Number	Proportion of operations (%)
W201	Primary open reduction of fracture of long bone and extramedullary fixation using plate NEC	5004	14.8
W283	Removal of internal fixation from bone NEC	1728	5.1
W202	Primary open reduction of fracture of long bone and extramedullary fixation using cerclage	1122	3.3
W373	Revision of total prosthetic replacement of hip joint using cement	843	2.5
W383	Revision of total prosthetic replacement of hip joint not using cement	633	1.9
W384	Revision of one component of total prosthetic replacement of hip joint not using cement	617	1.8
W192	Primary open reduction of fracture of long bone and fixation using rigid nail NEC	591	1.7
W382	Conversion to total prosthetic replacement of hip joint not using cement	588	1.7
W370	Conversion from previous cemented total prosthetic replacement of hip joint	585	1.7
W403	Revision of total prosthetic replacement of knee joint using cement	582	1.7
W374	Revision of one component of total prosthetic replacement of hip joint using cement	501	1.5
W901	Aspiration of joint	495	1.5
W281	Application of internal fixation to bone NEC	448	1.3
W232	Secondary open reduction of fracture of bone and extramedullary fixation HFQ	420	1.2
W322	Allograft of bone NEC	389	1.1
W246	Closed reduction of fracture of bone and fixation using nail or screw	333	1.0
W191	Primary open reduction of fracture of neck of femur and open fixation using pin and plate	310	0.9
W242	Closed reduction of fracture of long bone and rigid internal fixation NEC	300	0.9
W198	Other specified primary open reduction of fracture of bone and intramedullary fixation	298	0.9
W381	Primary total prosthetic replacement of hip joint not using cement	258	0.8
other	All other main procedures combined	33922	53.7

NEC = not elsewhere classified

Bottle A, *et al. BMJ Open* 2020; 10:e042371. doi: 10.1136/bmjopen-2020-042371
