# Supplementary material for: Periprosthetic fractures: the next fragility fracture epidemic? A national observational study
Source: BMJ Open. 2020 Dec 10;10(12):e042371. doi: 10.1136/bmjopen-2020-042371 (PMC7733197; doi:10.1136/bmjopen-2020-042371)
Supplement: Supplementary data [file bmjopen-2020-042371supp002.pdf]

Supplementary Table 2. Operative rate according to age, gender and method of admission

Factor	Elective admissions with operation	Emergency admissions with operation	% of all elective admissions with operation	% of all emergency admissions with operation
Age 0-44, female	65	98	95.6	66.7
Age 0-44, male	146	225	88.5	74.3
Age 45-64, female	229	636	95.0	76.9
Age 45-64, male	164	476	92.1	77.5
Age 65-84, female	552	4058	82.5	73.6
Age 65-84, male	304	2298	85.4	74.9
Age 85+, female	140	3249	64.5	67.0
Age 85+, male	56	1092	62.2	69.0
Total	1656	12132	83.5	71.8

Bottle A, *et al. BMJ Open* 2020; 10:e042371. doi: 10.1136/bmjopen-2020-042371
