# Supplementary material for: Periprosthetic fractures: the next fragility fracture epidemic? A national observational study
Source: BMJ Open. 2020 Dec 10;10(12):e042371. doi: 10.1136/bmjopen-2020-042371 (PMC7733197; doi:10.1136/bmjopen-2020-042371)
Supplement: Supplementary data [file bmjopen-2020-042371supp003.pdf]

Supplementary Table 3. Length of stay in nights according to major operation status

Patient group	LOS type	N	Lower quartile	Median	Mean	Upper quartile
All	Acute stay	18888	7	14	20.5	26
All	Total	18888	8	17	25.0	33
No major operation	Acute stay	5100	4	10	17.6	22
No major operation	Total	5100	4	11	19.8	26
Major operation	Acute stay	13788	9	16	21.6	27
Major operation	Total	13788	10	19	26.9	35

"Acute stay" means the stay at the first (acute) hospital; "Total" covers the whole admission, including interhospital transfers

Bottle A, *et al. BMJ Open* 2020; 10:e042371. doi: 10.1136/bmjopen-2020-042371
