# Supplementary material for: Periprosthetic fractures: the next fragility fracture epidemic? A national observational study
Source: BMJ Open. 2020 Dec 10;10(12):e042371. doi: 10.1136/bmjopen-2020-042371 (PMC7733197; doi:10.1136/bmjopen-2020-042371)
Supplement: Supplementary data [file bmjopen-2020-042371supp004.pdf]

Supplementary Table 4. Length of stay in nights by age and gender

Patient group	LOS type	N	Lower quartile	Median	Mean	Upper quartile
Age 0-44, female	Acute stay	215	1	3	5.6	8
	Total	215	1	3	6.8	9
Age 0-44, male	Acute stay	468	0	2	4.0	5
	Total	468	0	2	4.7	5
Age 45-64, female	Acute stay	1068	3.5	8	12.1	14.5
	Total	1068	4	9	14.6	17
Age 45-64, male	Acute stay	792	3	8	11.8	14
	Total	792	3	8	13.5	15
Age 65-84, female	Acute stay	6186	8	14	20.2	25
	Total	6186	9	17	24.6	31
Age 65-84, male	Acute stay	3424	7	14	19.5	24
	Total	3424	8	16	23.9	30
Age 85+, female	Acute stay	5063	11	19	25.2	32
	Total	5063	12	23	31.0	41
Age 85+, male	Acute stay	1672	11	19	25.7	33
	Total	1672	13	23	31.4	43

“Acute stay” means the stay at the first (acute) hospital; “Total” covers the whole admission, including interhospital transfers
