# Supplementary material for: Periprosthetic fractures: the next fragility fracture epidemic? A national observational study
Source: BMJ Open. 2020 Dec 10;10(12):e042371. doi: 10.1136/bmjopen-2020-042371 (PMC7733197; doi:10.1136/bmjopen-2020-042371)
Supplement: Supplementary data [file bmjopen-2020-042371supp005.pdf]

Supplementary Table 5. Length of stay in nights by age, gender and operative status

Patient group	LOS type	Operated	N	Lower quartile	Median	Mean	Upper quartile
Age 0-44, female	Acute stay	No	52	1	2	4.0	5
		Yes	52	1	3	6.1	8
	Total	No	163	1	2	4.4	6
		Yes	163	1	4	7.6	10
Age 0-44, male	Acute stay	No	97	0	1	3.2	3
		Yes	97	1	2	4.3	5
	Total	No	371	0	1	3.6	3
		Yes	371	1	2	5.0	6
Age 45-64, female	Acute stay	No	203	1	4	8.3	11
		Yes	203	4	9	13.0	15
	Total	No	865	1	4	10.2	13
		Yes	865	5	10	15.6	18
Age 45-64, male	Acute stay	No	152	1	3	7.4	7.5
		Yes	152	4	9	12.8	15
	Total	No	640	1	4	8.9	8
		Yes	640	4	10	14.5	17
Age 65-84, female	Acute stay	No	1576	3	10	16.4	20
		Yes	1576	9	16	21.4	26
	Total	No	4610	3	10	17.9	23
		Yes	4610	11	19	26.8	33
Age 65-84, male	Acute stay	No	822	2	8	15.1	19
		Yes	822	9	15	20.9	25
	Total	No	2602	2	8	16.5	21
		Yes	2602	10	18	26.1	32
Age 85+, female	Acute stay	No	1674	6	14	21.8	28
		Yes	1674	13	20	26.9	34
	Total	No	3389	7	16	25.3	33
		Yes	3389	15	26	33.7	44
Age 85+, male	Acute stay	No	524	7	14	21.9	27
		Yes	524	13	21	27.5	34
	Total	No	1148	7	16	24.9	34
		Yes	1148	16	26	34.3	46

"Acute stay" means the stay at the first (acute) hospital; "Total" covers the whole admission, including interhospital transfers

Bottle A, *et al. BMJ Open* 2020; 10:e042371. doi: 10.1136/bmjopen-2020-042371
