# Supplementary figures and images for: Periprosthetic fractures: the next fragility fracture epidemic? A national observational study
Source: BMJ Open. 2020 Dec 10;10(12):e042371. doi: 10.1136/bmjopen-2020-042371 (PMC7733197; doi:10.1136/bmjopen-2020-042371)

Supplementary Figure 1. Kaplan Meier Survival Curve by sex

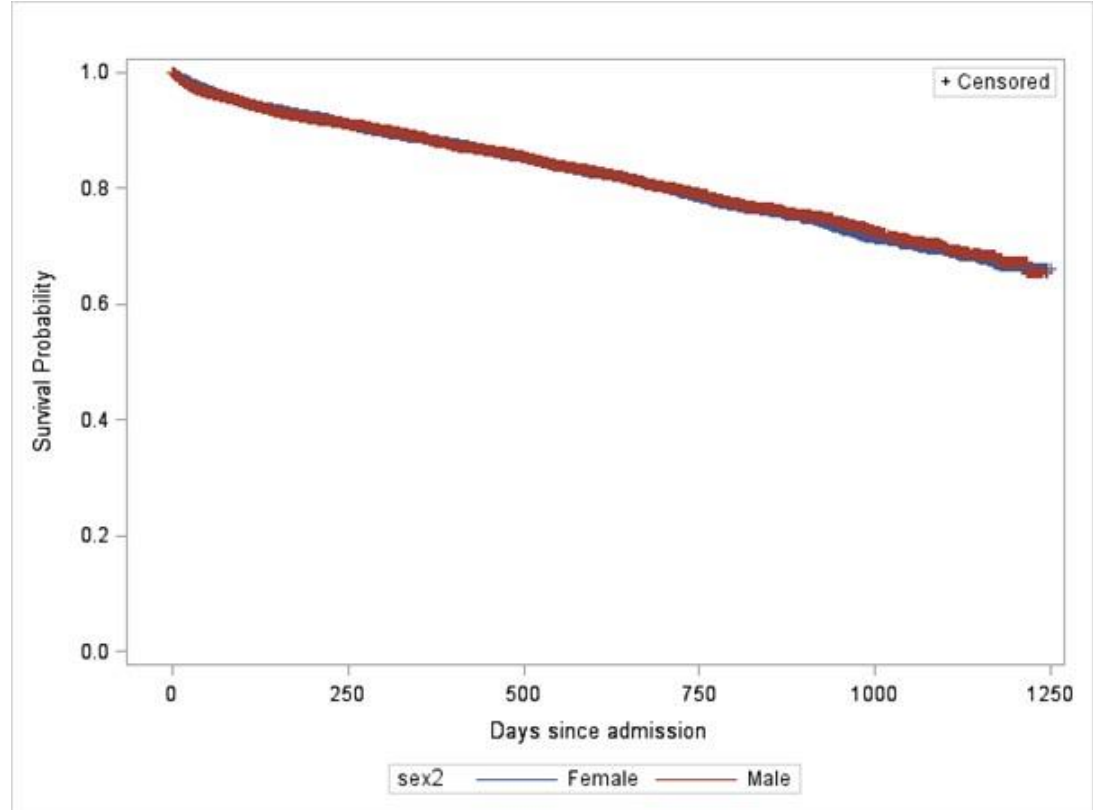

Supplement: Supplementary data [file bmjopen-2020-042371supp006.pdf]

Supplementary Figure 2. Kaplan Meier Survival Curve by age range

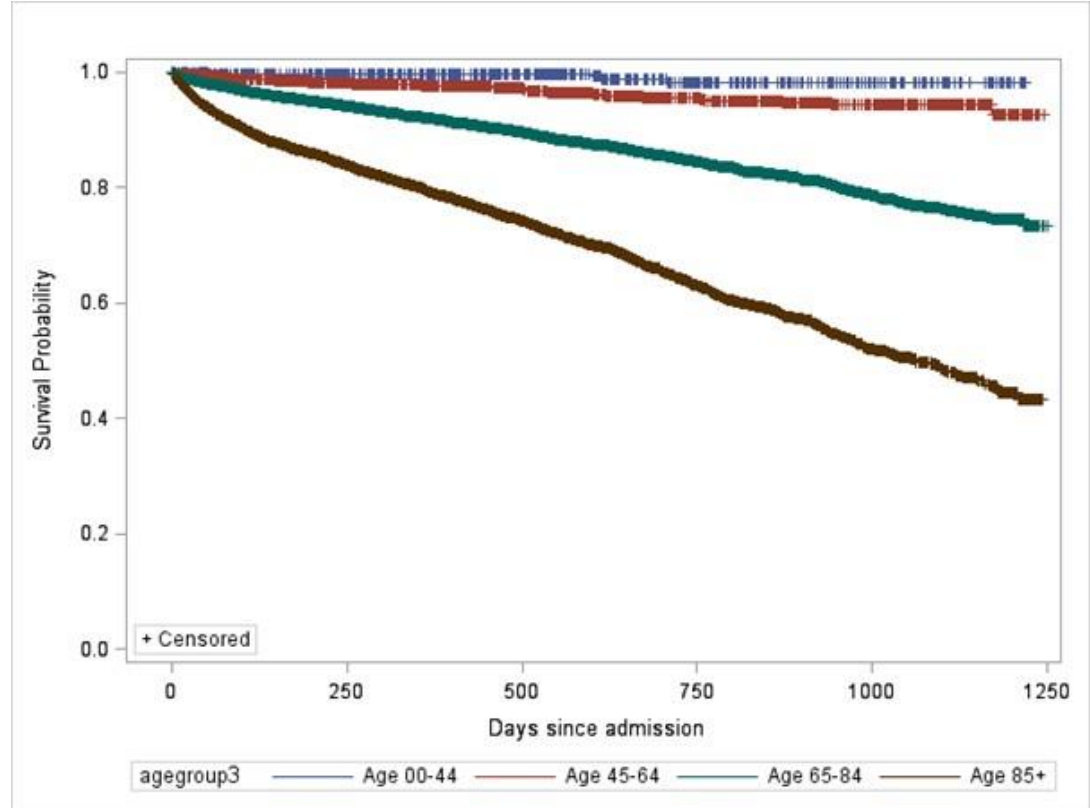

Supplement: Supplementary data [file bmjopen-2020-042371supp007.pdf]
