# Supplementary material for: Periprosthetic fractures: the next fragility fracture epidemic? A national observational study
Source: BMJ Open. 2020 Dec 10;10(12):e042371. doi: 10.1136/bmjopen-2020-042371 (PMC7733197; doi:10.1136/bmjopen-2020-042371)
Supplement: Author's manuscript [file bmjopen-2020-042371.draft_revisions.pdf]

BMJ Open is committed to open peer review. As part of this commitment we make the peer review history of every article we publish publicly available.

When an article is published we post the peer reviewers' comments and the authors' responses online. We also post the versions of the paper that were used during peer review. These are the versions that the peer review comments apply to.

The versions of the paper that follow are the versions that were submitted during the peer review process. They are not the versions of record or the final published versions. They should not be cited or distributed as the published version of this manuscript.

BMJ Open is an open access journal and the full, final, typeset and author-corrected version of record of the manuscript is available on our site with no access controls, subscription charges or pay-per-view fees (http://bmjopen.bmj.com).

If you have any questions on BMJ Open's open peer review process please email info.bmjopen@bmj.com

BMJ Open

# Periprosthetic fractures – the next fragility fracture epidemic? A national observational study



| | |
|---|---|
| Journal: | *BMJ Open* |
| Manuscript ID | bmjopen-2020-042371 |
| Article Type: | Original research |
| Date Submitted by the Author: | 02-Jul-2020 |




| | |
|---|---|
| Complete List of Authors: | Bottle, Alex; Imperial College, Primary Care and Social Medicine<br>Griffiths, Richard; Peterborough City Hospital, Anaesthesia<br>White, Stuart; Royal Sussex County Hospital,<br>Wynn-Jones, Henry; Wrightington Hospital, The Centre for Hip Surgery<br>Aylin, Paul; Imperial College London, Epidemiology and Public Health<br>Moppett, Iain; University of Nottingham, Anaesthesia & Critical Care<br>Chowdhury, Emyr; North West Anglia NHS Foundation Trust<br>Wilson, Helen; Royal Surrey County Hospital NHS Foundation Trust, Department of Geriatric Medicine<br>Davies, Benjamin; University of Cambridge, Department of Surgery |




| | |
|---|---|
| Keywords: | Adult orthopaedics < ORTHOPAEDIC & TRAUMA SURGERY, EPIDEMIOLOGY, GERIATRIC MEDICINE |


SCHOLARONE™
Manuscripts

*I, the Submitting Author has the right to grant and does grant on behalf of all authors of the Work (as defined in the below author licence), an exclusive licence and/or a non-exclusive licence for contributions from authors who are: i) UK Crown employees; ii) where BMJ has agreed a CC-BY licence shall apply, and/or iii) in accordance with the terms applicable for US Federal Government officers or employees acting as part of their official duties; on a worldwide, perpetual, irrevocable, royalty-free basis to BMJ Publishing Group Ltd ("BMJ") its licensees and where the relevant Journal is co-owned by BMJ to the co-owners of the Journal, to publish the Work in this journal and any other BMJ products and to exploit all rights, as set out in our licence.*

*The Submitting Author accepts and understands that any supply made under these terms is made by BMJ to the Submitting Author unless you are acting as an employee on behalf of your employer or a postgraduate student of an affiliated institution which is paying any applicable article publishing charge ("APC") for Open Access articles. Where the Submitting Author wishes to make the Work available on an Open Access basis (and intends to pay the relevant APC), the terms of reuse of such Open Access shall be governed by a Creative Commons licence – details of these licences and which Creative Commons licence will apply to this Work are set out in our licence referred to above.*

*Other than as permitted in any relevant BMJ Author's Self Archiving Policies, I confirm this Work has not been accepted for publication elsewhere, is not being considered for publication elsewhere and does not duplicate material already published. I confirm all authors consent to publication of this Work and authorise the granting of this licence.*

For peer review only

Title:
# Periprosthetic fractures – the next fragility fracture epidemic? A national observational study

Authors:
Alex Bottle[1] (robert.bottle@imperial.ac.uk)
Richard Griffiths[2] (richardgriffiths1@nhs.net)
Stuart M White[3] (igasbest@hotmail.com)
Henry Wynn-Jones[4] (henrywynnjones@gmail.com)
Paul Aylin[1] (p.aylin@imperial.ac.uk)
Iain Moppett[5] (iain.moppett@nottingham.ac.uk)
Emyr A H Chowdhury[6] (emyr.chowdhury@nhs.net)
Helen Wilson[7] (hwilson6@nhs.net)
Benjamin M Davies[8] (bmd35@cam.ac.uk)

Affiliations:
1 – Dr Foster Unit, Dept of Epidemiology and Public Health, School of Public Health, Imperial College London, Reynolds Building, St Dunstan's Road, London, United Kingdom
2 – Department of Anaesthesia, Peterborough City Hospital, Peterborough, United Kingdom
3 – Haywards Heath Hip Fracture Unit, Princess Royal Hospital, Brighton and Sussex University Hospitals NHS Trust, Haywards Heath, United Kingdom
4 – The Centre for Hip Surgery, Wrightington Hospital, Hall Lane, Appley Bridge, Lancashire, United Kingdom
5 – Anaesthesia and Critical Care, Division of Clinical Neuroscience, The University of Nottingham, Queen's Medical Centre, Nottingham, United Kingdom
6 – Department of Trauma and Orthopaedics, Peterborough City Hospital, Peterborough, United Kingdom
7 – Department of General Medicine, Stroke and Care of the Elderly, Royal Surrey County Hospital, Guildford, United Kingdom
8 – Division of Trauma and Orthopaedics, University of Cambridge, Addenbrooke's Hospital, Cambridge, United Kingdom

Corresponding Author:
Mr Benjamin Davies, Division of Trauma and Orthopaedics, University of Cambridge, Box 180, Addenbrooke's Hospital, Hills Road, Cambridge, CB2 2QQ, United Kingdom
Email: bmd35@cam.ac.uk
Telephone: 07974 192502

Keywords:
Periprosthetic fracture
Fragility fracture
Trauma
Hospital Episode Statistics

Word Count: 3,686

For peer review only

Abstract:
Objectives –
Periprosthetic fractures have considerable clinical implications for patients and financial implications for healthcare systems. This study aims to determine the burden of periprosthetic fractures of the lower and upper limbs in England and identify any factors associated with differences in treatment and outcome.
Design –
A national, observational study
Setting –
England
Participants –
All individuals admitted to hospital with peri-prosthetic factures between 01/04/2015 and 31/12/2018.
Primary and Secondary Outcome Measures –
Mortality, length of stay, change in rate of admissions.
Methods –
We analysed Hospital Episode Statistics (HES) data using the ICD-10 code M96.6 ("Fracture of bone following insertion of orthopaedic implant, joint prosthesis, or bone plate") to identify periprosthetic fractures recorded between April 2013 and December 2018. We determined the demographics, procedures performed, mortality rates, and discharge destinations. Patient characteristics associated with having a procedure during the index admission were estimated using logistic regression. The annual rate of increase in admissions was estimated using Poisson regression.
Results –
Between 01/04/2015 and 31/12/2018, there were 13,565 patients who had 18,888 admissions (89.5% emergency) with M96.6 in the primary diagnosis field. There was a 13% year-on-year increase in admissions for periprosthetic fracture in England during that period. Older people, people living in deprived areas, and those with heart failure or neurological disorders were less likely to receive an operation. 14.4% of patients did not return home after hospital discharge. The overall in-patient mortality was 4.3% and total 30-day mortality 3.3%.
Conclusions –
The clinical and operational burden of periprosthetic fractures is considerable and increasing rapidly. We suggest that the management of people with periprosthetic fractures should be undertaken and funded in a similar manner to that successfully employed for people sustaining hip fractures, utilising national standards and data collection to monitor and improve performance.

**Article Summary - strengths and limitations of this study**

- This is the first study to use ICD-10 code M96.6 to identify trends in peri-prosthetic fracture rates.
- This data can be used to help develop specific management pathways and allocate resources.
- Use of HES data is limited by potential inaccuracies in coding.
- The study data does not include functional outcomes which would provide useful information.



For peer review only - http://bmjopen.bmj.com/site/about/guidelines.xhtml

## Introduction

Since the successful introduction of total hip replacement in the 1960s, joint replacement has revolutionised the treatment of severely arthritic joints.[1] Joint replacement prostheses are available for all the major joints of the upper and lower limbs. In 2018, approximately 200,000 arthroplasty procedures were recorded in the 16th Annual report of the National Joint Registry (NJR) for England and Wales.[2] Between the advent of the registry, on 1st April 2003, and 31st December 2018, 2.75 million joint replacement procedures have been recorded. In addition, 28,684 femoral hemiarthroplasties were performed in 2018 for proximal femoral fractures.[3] The increasing life expectancy in the UK[4], combined with the effects of chronic disease and frailty[5] lead to an increased likelihood of sustaining a fracture due to osteoporosis and an increased incidence of falls. Fractures can also occur around joint replacements, and these are termed periprosthetic fractures. Periprosthetic fractures may require either fixation or revision of the implant. Surgery to treat such fractures is often complex and/or prolonged, requiring specialist input from a multidisciplinary team.

There has been no comprehensive assessment of the burden of these injuries upon the healthcare system in England. The NJR collects data on revision arthroplasty and identifies those performed for peri-prosthetic fracture. Fractures treated without the need to revise the implant are not recorded at present in the NJR. The alternative sources of data are the UK's four national hospital administrative databases: Hospital Episodes Statistics (HES) for England, and similar systems for each of the other three countries. Clinical coders use ICD-10 and UK-specific OPCS coding systems to record diagnosis and procedure information respectively. Owing to the complex classification of fractures and different techniques to treat them, there are many operative codes that could be used. In March 2015, NHS England issued new guidance to clinical coders regarding periprosthetic fractures.[6] The new ICD10 code M96.6 ("Fracture of bone following insertion of orthopaedic implant, joint prosthesis, or bone plate") is designed to capture all patients who have experienced a periprosthetic fracture at any time during an episode of care, regardless of site or operative treatment. It also captures fractures around fixation devices such as plates and intramedullary nails.

The purpose of this observational study is to quantify the burden of these cases on the NHS and identify any trends in the treatment of these conditions which may inform future healthcare resource planning. Using England's national hospital administrative database, HES, we describe the admissions with the new M96.6 diagnosis code, time trends, patient characteristics, operative rate and short-term outcomes. A literature review carried out by the authors did not identify any previous reporting of these national level data.

## Methods

Data

Hospital Episode Statistics (HES)[7] comprises over 125 million admitted patient, outpatient and emergency department records from hospitals within England's National Health Service (NHS) annually. Inpatient records consist of consultant episodes: each episode covers the period during which a patient is the responsibility of a given physician. A single admission at a given hospital may comprise multiple episodes. Episodes were linked into admissions and linkage with inter-hospital transfers creates "superspells"; we will use the term "admission" throughout to refer to superspells. HES provides data on in-hospital mortality and is linked to the national death register. One primary and up to 19 secondary diagnoses are coded using ICD-10;[8] one primary and up to 23 secondary procedures are coded using the UK's own "OPCS" coding system. Area-level socio-economic deprivation status in population-weighted



For peer review only - http://bmjopen.bmj.com/site/about/guidelines.xhtml

quintiles was linked to HES via the patient's postcode. Coding is completed by specialist clinical coders (rather than clinicians) in hospitals from the medical record. Once received centrally the data is cleaned to remove duplicates.

We extracted all admissions from HES with the ICD-10 code M96.6 in the primary diagnosis field (which corresponds to the main problem treated) and discharge dates between April 2013 and December 2018.

Outcome measures

We report the primary procedure performed, length of stay (acute hospital and superspell), final destination on discharge, in-hospital mortality rate and total 30-day mortality rate.

We also report the proportion of admissions with a procedure, excluding scans via the U chapter of OPCS (Diagnostic Imaging, Testing and Rehabilitation), urinary catheterisation, and auxiliary or minor procedures via the X, Y and Z chapters of OPCS (Miscellaneous and Subsidiary Classification of Methods and Sites of Operation).

Statistical analysis

With any coding change, some variation in the uptake rate between hospitals is expected. We tried to separate the uptake of the new code from any genuine underlying change in the number of admissions by dividing hospital trusts into "early adopters" and the rest. "Early adopters" were defined as those with two or more discharges with M96.6 as the primary diagnosis in March or April 2015. Simple Poisson regression models estimated the linear trend in admissions for each group of trusts ("early adopters" and "other") and for all combined. As a sensitivity analysis the modelling was repeated after excluding the first six months of the new coding period, i.e. including only admissions from October 2015 onwards.

Patient characteristics were described for the cohort from April 2015 including: age, gender, method of admission (whether emergency or not), area-level socio-economic deprivation[9] and comorbidity (using the HES ICD-10 secondary diagnosis codes). The proportion of admissions with a procedure coded, as defined above, was noted and the factors associated with a procedure derived from a logistic regression model with age, gender, deprivation and comorbidities as covariates entered at once with no selection or elimination.

Ethical Approval

No ethical approval was required for this study which deals only in national-level aggregate data.

**Results**

Between 1st April 2013 and 31st December 2018, there were 24,697 admissions with M96.6 recorded in any diagnosis field. After the coding guidance was introduced in March 2015, there were 22,955 such admissions (84.5% classified as emergency) for 16,105 patients. For the 80.3% who had M96.6 in the primary diagnosis field, there were 18,888 admissions (89.5% emergency) for 13,565 patients.



For peer review only - http://bmjopen.bmj.com/site/about/guidelines.xhtml

Change in admissions over time

Table 1 gives the number of admissions by year, split by whether M96.6 was in the primary or in the secondary diagnosis fields.

*Table 1: Total numbers of admissions with M96.6 recorded since April 2013*

| Financial year (Apr 1 to Mar 31) | Numbers of admissions with M96.6 as primary diagnosis | Numbers of admissions with M96.6 only as secondary diagnosis |
|---|---|---|
| 2013/4 | 429 | 384 |
| 2014/5 | 514 | 415 |
| 2015/16 | 3832 | 974 |
| 2016/17 | 5232 | 1077 |
| 2017/18 | 5435 | 1123 |
| 2018/19 to Dec 31 2018 | 4389 (5852 full year pro rata) | 893 (1191 full year pro rata) |
| Total | 19831 | 4866 |

The subsequent analysis is restricted to admissions with M96.6 in the primary diagnosis.

Figure 1 illustrates admissions by month since April 2014 to December 2018, with elective and emergency admissions shown separately. The rapid rise in admissions from April 2015 coincides with the introduction of the M96.6 coding guidance.

<Figure 1 here>

116 out of 141 hospital trusts using the M96.6 code were labelled early adopters. The rate of increase (slope) for the non-early adopters was significantly higher than that for the early adopters (p=0.001 for the interaction term). Taking the early adopters as being the "true" underlying trend, there was a 13% annual rise in M96.6 admissions between April 2015 and December 2018 (Table 2). Excluding the first six months since the coding change did not change the rate of increase significantly.

*Table 2: Poisson regression showing annual increase in use of M96.6 diagnosis code nationally and split by whether hospitals were early adopters of this code*

| Set of trusts | Relative Risk per month | 95% CI | Relative Risk per year | 95% CI |
|---|---|---|---|---|
| All | 1.011 | 1.010 - 1.012 | 1.14 | 1.13-1.15 |
| Early M96.6 adopters | 1.010 | 1.009 - 1.011 | 1.13 | 1.11-1.14 |
| Non early adopters | 1.016 | 1.013 - 1.018 | 1.21 | 1.17-1.24 |
| All (Oct 2015 onwards only) | 1.009 | 1.008 - 1.010 | 1.11 | 1.10-1.13 |

Patient Characteristics

The majority of admissions were for women (66.4% female, 33.6% male). Table 3 details patient characteristics.



For peer review only - http://bmjopen.bmj.com/site/about/guidelines.xhtml

*Table 3: Patient Characteristics*

| Factor | Admissions | Proportion of total admissions (%) |
|---|---|---|
| Age-sex | | |
| Age 0-44, female | 215 | 1.1 |
| Age 0-44, male | 468 | 2.5 |
| Age 45-64, female | 1068 | 5.7 |
| Age 45-64, male | 792 | 4.2 |
| Age 65-84, female | 6186 | 32.8 |
| Age 65-84, male | 3424 | 18.1 |
| Age 85+, female | 5063 | 26.8 |
| Age 85+, male | 1672 | 8.9 |
| Deprivation | | |
| Quintile 1 (least deprived) | 4796 | 25.4 |
| Quintile 2 | 4467 | 23.6 |
| Quintile 3 | 4090 | 21.7 |
| Quintile 4 | 3143 | 16.6 |
| Quintile 5 (most deprived) | 2300 | 12.2 |
| Quintile 6 (not known) | 92 | 0.5 |
| Dementia | 2634 | 13.9 |
| Alcohol misuse | 625 | 3.3 |
| Arrhythmias | 4130 | 21.9 |
| Heart failure | 1445 | 7.7 |
| COPD | 3337 | 17.7 |
| Deficiency anaemia | 493 | 2.6 |
| Depression | 1231 | 6.5 |
| Diabetes mellitus | 2877 | 15.2 |
| Fluid disorders | 1396 | 7.4 |
| Hypertension | 9208 | 48.8 |
| Hypothyroidism | 1804 | 9.6 |
| Liver disease | 229 | 1.2 |
| Cancer with metastasis | 251 | 1.3 |
| Obesity | 784 | 4.2 |
| Other neurological conditions | 1204 | 6.4 |
| Paraplegia | 180 | 1 |
| Pulmonary circulatory disorders | 204 | 1.1 |
| PVD | 520 | 2.8 |
| Renal disease | 2189 | 11.6 |
| Rheumatological disorders | 1619 | 8.6 |
| Solid tumours | 650 | 3.4 |
| Valvular disease | 1091 | 5.8 |

Operative rate and main procedures performed

Given that the coding advice only came into force in March 2015, we now restrict the analysis to the 11,019 discharges from April 2015 onwards, again with M96.6 as the primary diagnosis.


For peer review only - http://bmjopen.bmj.com/site/about/guidelines.xhtml
For peer review only

Primary open reduction [W201 + W192 + W202 + W191 + W198] was the commonest recorded surgical technique (21.6%); revision of the prosthesis [W373 + W383 + W384 + W374 + W403 + W393 + W404] was performed in 10.4% (the commonest procedures are listed in Supplementary Table 1).

The overall operative rates were 74.7% in 2015/2016, 72.1% in 2016/17 and 74.5% in 2017/2018. For the elective admissions, 83.5% had a procedure performed; for the emergency admissions, 71.8% had a procedure performed, with an overall proportion of 73.0%.

Table 4 gives the operative rate by patient characteristic. Formal statistical analysis of the rate of operative intervention was performed using logistic regression (See Supplementary Table 2 for operative rates by age, gender, and method of admission). Patients of both sexes aged greater than 84 years were significantly less likely to have an operation than the youngest age group. Surgery was also less likely with increasing socio-economic deprivation, Chronic Obstructive Pulmonary Disease (COPD), and neurological disorders. Surgery was more likely in the presence of recorded fluid and electrolyte disturbance, hypertension, and obesity (OR 1.78, 95% CI 1.46-2.17).

*Table 4: Operative rate by patient characteristics*

| Factor | No operation | Operation | Proportion with operation (%) | Adjusted OR for operation | P value |
|---|---|---|---|---|---|
| Age 00-44, female | 52 | 163 | 75.8 | 1.23 (0.83 to 1.81) | 0.299 |
| Age 00-44, male | 97 | 371 | 79.3 | 1 | |
| Age 45-64, female | 203 | 865 | 81.0 | 1.29 (0.91 to 1.83) | 0.158 |
| Age 45-64, male | 152 | 640 | 80.8 | 1.27 (0.88 to 1.82) | 0.206 |
| Age 65-84, female | 1576 | 4610 | 74.5 | 0.86 (0.62 to 1.19) | 0.371 |
| Age 65-84, male | 822 | 2602 | 76.0 | 0.97 (0.69 to 1.34) | 0.830 |
| Age 85+, female | 1674 | 3389 | 66.9 | 0.6 (0.44 to 0.83) | 0.002 |
| Age 85+, male | 524 | 1148 | 68.7 | 0.67 (0.48 to 0.94) | 0.019 |
| Quintile 1 (least deprived) | 1267 | 3529 | 73.6 | 1 | |
| Quintile 2 | 1183 | 3284 | 73.5 | 1.00 (0.91 to 1.10) | 0.998 |
| Quintile 3 | 1082 | 3008 | 73.5 | 0.98 (0.89 to 1.08) | 0.742 |
| Quintile 4 | 882 | 2261 | 71.9 | 0.89 (0.80 to 0.98) | 0.025 |
| Quintile 5 (most deprived) | 655 | 1645 | 71.5 | 0.84 (0.75 to 0.95) | 0.004 |
| Quintile 6 (not known) | 31 | 61 | 66.3 | 0.64 (0.41 to 1.01) | 0.053 |
| Alcohol misuse | 151 | 474 | 75.8 | 0.93 (0.76 to 1.14) | 0.484 |
| Arrhythmias | 1208 | 2922 | 70.8 | 0.96 (0.89 to 1.05) | 0.385 |
| COPD | 879 | 2458 | 73.7 | 0.84 (0.74 to 0.96) | 0.008 |
| Cancer with metastasis | 65 | 186 | 74.1 | 1.02 (0.93 to 1.11) | 0.681 |
| Deficiency anaemia | 150 | 343 | 69.6 | 0.89 (0.73 to 1.08) | 0.229 |
| Dementia | 910 | 1724 | 65.5 | 0.88 (0.77 to 1.00) | 0.055 |



For peer review only - http://bmjopen.bmj.com/site/about/guidelines.xhtml

| | | | | | |
|---|---|---|---|---|---|
| Depression | 319 | 912 | 74.1 | 1.02 (0.89 to 1.16) | 0.830 |
| Diabetes mellitus | 777 | 2100 | 73.0 | 0.98 (0.89 to 1.07) | 0.613 |
| Fluid disorders | 340 | 1056 | 75.6 | 1.31 (1.15 to 1.49) | <0.001 |
| Heart failure | 466 | 979 | 67.8 | 1.13 (1.06 to 1.21) | 0.0004 |
| Hypertension | 2424 | 6784 | 73.7 | 1.07 (0.95 to 1.19) | 0.264 |
| Hypothyroidism | 483 | 1321 | 73.2 | 0.98 (0.71 to 1.35) | 0.907 |
| Liver disease | 56 | 173 | 75.5 | 1.03 (0.75 to 1.42) | 0.868 |
| Obesity | 125 | 659 | 84.1 | 1.78 (1.46 to 2.17) | <0.001 |
| Other neurological conditions | 362 | 842 | 69.9 | 0.80 (0.70 to 0.91) | 0.001 |
| Peripheral vascular disease | 143 | 377 | 72.5 | 0.87 (0.63 to 1.21) | 0.401 |
| Paraplegia | 54 | 126 | 70.0 | 1.28 (0.92 to 1.79) | 0.145 |
| Pulmonary circulatory disorders | 49 | 155 | 76.0 | 1.00 (0.82 to 1.23) | 0.974 |
| Renal disease | 637 | 1552 | 70.9 | 0.98 (0.88 to 1.09) | 0.695 |
| Rheumatological disorders | 455 | 1164 | 71.9 | 0.90 (0.80 to 1.01) | 0.065 |
| Solid tumours | 182 | 468 | 72.0 | 0.96 (0.79 to 1.17) | 0.699 |
| Valvular disease | 297 | 794 | 72.8 | 1.11 (0.96 to 1.28) | 0.161 |

For peer review only

## Length of Stay

Median acute hospital LOS was 14 nights (IQR 7-26), and median total hospital LOS was 17 nights (IQR 8-33: Supplementary table 3). Acute hospital LOS increased with increasing age and was higher for older ages for both sexes and for operative than non-operative management at each age group (p<0.001; Figures 2 and 3 and Supplementary tables 3, 4 and 5).

<Figure 2 here>

<Figure 3 here>

To determine what factors were associated with increased LOS a logistic regression was performed. In the absence of an accepted definition of extended LOS in these cases we used the Upper Quartile boundary (26) to define a long LOS. Increasing age was significantly associated with a longer length of stay. A wide variety of co-morbidities were also associated with a longer LOS, but with a smaller effect than that of age (See Table 5).

*Table 5: Factors associated with a long LOS (≥26 nights)*

| Factor | OR (95% CI) | P value |
|---|---|---|
| Age 00-44, female | 0.66 (0.21 to 2.12) | 0.489 |
| Age 00-44, male | 1 | |
| Age 45-64, female | 4.41 (1.77 to 10.95) | 0.001 |
| Age 45-64, male | 3.97 (1.58 to 9.95) | 0.003 |
| Age 65-84, female | 11.01 (4.52 to 26.86) | <0.001 |
| Age 65-84, male | 9.95 (4.07 to 24.32) | <0.001 |
| Age 85+, female | 17.93 (7.35 to 43.74) | <0.001 |
| Age 85+, male | 17.00 (6.93 to 41.66) | <0.001 |
| Quintile 1 (least deprived) | 1 | |

| | | |
|---|---|---|
| Quintile 2 | 1.03 (0.93 to 1.13) | 0.603 |
| Quintile 3 | 1.02 (0.92 to 1.13) | 0.689 |
| Quintile 4 | 1.09 (0.98 to 1.22) | 0.115 |
| Quintile 5 (most deprived) | 1.15 (1.02 to 1.30) | 0.027 |
| Quintile 6 (not known) | 0.88 (0.50 to 1.53) | 0.642 |
| Alcohol misuse | 1.14 (0.92 to 1.42) | 0.221 |
| Arrhythmias | 1.27 (1.17 to 1.38) | <0.001 |
| COPD | 1.32 (1.17 to 1.50) | <0.001 |
| Cancer with metastasis | 1.10 (1.00 to 1.20) | 0.039 |
| Deficiency anaemia | 1.44 (1.18 to 1.74) | 0.003 |
| Dementia | 1.43 (1.26 to 1.63) | <0.001 |
| Depression | 1.10 (0.96 to 1.27) | 0.187 |
| Diabetes mellitus | 1.19 (1.09 to 1.31) | 0.0002 |
| Fluid disorders | 2.29 (2.04 to 2.56) | <0.001 |
| Heart failure | 1.03 (0.96 to 1.10) | 0.493 |
| Hypertension | 1.00 (0.89 to 1.12) | 0.949 |
| Hypothyroidism | 2.08 (1.54 to 2.81) | <0.001 |
| Liver disease | 0.83 (0.60 to 1.15) | 0.270 |
| Obesity | 1.25 (1.05 to 1.48) | 0.012 |
| Other neurological conditions | 1.47 (1.28 to 1.69) | <0.001 |
| Peripheral vascular disease | 2.12 (1.53 to 2.92) | <0.001 |
| Paraplegia | 1.73 (1.29 to 2.32) | 0.0003 |
| Pulmonary circulatory disorders | 1.05 (0.86 to 1.28) | 0.652 |
| Renal disease | 1.20 (1.08 to 1.33) | 0.0004 |
| Rheumatological disorders | 1.17 (1.04 to 1.32) | 0.012 |
| Solid tumours | 1.68 (1.39 to 2.04) | <0.001 |
| Valvular disease | 1.30 (1.13 to 1.49) | 0.0002 |

Final destination on discharge

89.3% of identified patients were admitted from their usual residence and 74.9% went back there afterwards: 8.9% went to a care home; 5.3% were discharged to a temporary residence; 4.3% died in hospital and 3.6% went to another hospital but for which no HES record could be linked.

The total 30-day mortality rate was 3.3% for the period April 1 2015 to June 30 2018 (deaths via linkage to the national deaths registry were available up to dates including July 31 2018).

Figure 4 shows Kaplan Meier survival curves for non-operative versus operative cases. See supplementary figures 1 & 2 for Kaplan Meier survival curves by sex and age range respectively.

<Figure 4 here>

**Discussion**

This study sought to establish the burden of periprosthetic fractures on the healthcare system in the UK and identify any factors associated with differences in treatment or outcome. The

For peer review only

number of patients admitted to hospital in England with periprosthetic fractures is currently around 450 per month. The overall number of periprosthetic fractures as coded by M96.6 is increasing by approximately 13% each year. Periprosthetic fractures are associated with a relatively prolonged length of stay and increased dependency upon discharge with 25% unable to return directly to their normal residence. 73% of people admitted with a periprosthetic fracture received an operation. Non-operative management was more common in those with co-morbidities, increasing age, and COPD. Mortality was low, at 4.3% for in-hospital and 3.3% for total 30-day mortality; for proximal femoral fractures, 30-day mortality from the latest NHFD report is higher, at 6.1%[3].

There are limitations to the data, and we have tried not to over-interpret the data we have. We cannot be certain how complete and accurate the M96.6 coding is. The study relies on accurate data recorded by clinicians and then coded by clinical coders in hospitals. The primary diagnosis and procedure fields in administrative data are known to have high accuracy (>95%)[10], though secondary diagnoses are subject to some under-recording. There will be variation between hospitals as to when the coding advice was implemented, which is why we estimated the trend in two ways. We have attempted to correct for this by analysing 'early adopters' separately from the complete cohort. Reassuringly, the conclusions are broadly the same in terms of absolute numbers and relative rate of rise. We did not attempt to distinguish between the sites of periprosthetic fractures, and some of these fractures will have been following arthroplasty of other joints, and fracture fixation, particularly neck of femur fracture.[11] M96.6 is not associated with an anatomical code to specify the bone and or joint involved. It is possible to attempt to infer the joint involved by any subsequent operation codes (M96.6 itself has no anatomical modifier), but this method is not guaranteed to be accurate and does not provide any information regarding the site of injury of those who were treated non-operatively (approximately 27% of all cases). Nonetheless, despite these limitations, we are confident that the increasing trend amongst older people with joint implants accurately reflects the everyday clinical situation.

HES data only provide in-hospital mortality, but we were able to use the established linkage to the national death registry for deaths up to the end of July 2018. More importantly, there are no direct data on functional outcomes.[12] Such additional data would be invaluable to allow clinicians and patients to discuss the most appropriate management.

This is the first study utilising the specific clinical code M96.6. This was introduced in March 2015, and although Figure 1 shows a dramatic increase when the coding advice was introduced, we are confident that there is a significant increasing underlying trend in patients with periprosthetic fractures. There have been few studies that have examined the periprosthetic patient population from a multidisciplinary perspective. There are no studies examining risk assessment for these patients with the exception of a small retrospective analysis, 39 patients with distal femoral fractures from Cleveland Ohio.[13]

Our data are in line with previous studies of complication rates which have demonstrated periprosthetic fracture rates of 0.1-18% for hip, 0.3-5.5% for knee, and 0.5-3% for shoulder arthroplasties.[14]

The number of peri-prosthetic fractures is likely to increase, given the ageing population, increased life expectancy and increasing numbers of people with joint replacements. How these individuals are managed is one of the issues that needs to be addressed in order to

For peer review only



For peer review only - http://bmjopen.bmj.com/site/about/guidelines.xhtml

provide optimum care. The likely treatment of these injuries varies depending on the location involved and the stability of the implant.

The extensive surgery involved in the operative treatment options for these individuals mean that these patients have to be able to withstand a more significant surgical insult than individuals undergoing primary joint arthroplasty or those being treated for neck of femur fractures. There are also implications for the use of hospital resources with longer operation times, expensive implants, and the availability of revision arthroplasty specialists in addition to the higher cost of post-operative care, including high dependency care.[15]

In-hospital mortality is significant, at around 5%, and the rate of new discharge to a care home of around 15% suggests an increase in dependency of this patient group – this representing those who whilst admitted from their usual residence were discharged to a different location.

For patients presenting with hip fracture, surgical management is almost always viewed as gold standard care for pain relief and to restore mobility where possible.  Early surgery is important to allow the patient to sit up thereby reducing risk of respiratory problems, malnutrition and difficulty in toileting.  This may be different in peri-prosthetic fractures, particularly those around the knee as patients may be able to tolerate sitting up and pain may be controlled by immobilising the fracture in a cast or brace.

Some patients with frailty do not have the physiological reserve to survive major surgery and this will influence decisions between non-operative management, fixation, and major revision surgery.

For all patients with frailty, either cognitive or physical, the decision regarding surgery should be made with the individual and the multidisciplinary team and in some cases may be viewed as a palliative procedure to manage pain.

Notwithstanding the limitations of our analysis, periprosthetic fractures are likely to create an increasing burden to patients, clinicians and the wider health and social care services. At current rates, the average UK trust will admit around one patient with a periprosthetic fracture each week. If the current trend of a 13% annual increase continues, this will rise to around 2 patients per week in 5- or 6-years' time, equating to around 700 extra bed-days per trust per year, alongside an increase in required operating theatre capacity. These patients are often complex, both surgically and medically, and hospitals may wish to consider whether a specialist local or regional service is provided to ensure optimal decision making and management. The costs associated with treating periprosthetic fractures often outstrips reimbursement from funders and so any such service will need to be designed in conjunction with funders to ensure it is financially viable.[16]

Data collection and accurate reporting are going to be needed to identify all of the cases, and an add-on to the NJR is required for complete data capture. If a comparison with proximal femoral fracture is made, the NHS is probably where it was with hip fracture care around 15 years ago, when the care of periprosthetic fractures is concerned. The great improvement in hip fracture care ran in parallel with the emergence of "orthogeriatrics" as a definite entity. Periprosthetic fractures will need the same level of multidisciplinary input that is adequately funded and using all the experience gained with the hip fracture population.


For peer review only - http://bmjopen.bmj.com/site/about/guidelines.xhtml

Will individual units be able to cope with demand or will there be regional specialisation? The resources needed will not be limited to orthopaedic surgery, where there will need to be enough adequately trained arthroplasty revision surgeons, but will include perioperative medicine, orthogeriatric input, high dependency care, nursing, physiotherapy and care home provision. Perhaps, dedicated units that deal with hip fractures and periprosthetic fractures will be the model of care for the future, with expertise and training opportunities concentrated in purpose-built units.

The coding change made in 2015 to make the NHS ICD-10 compliant has made the identification of periprosthetic fractures easier. There already appears to be an increase of 15% per year in these cases. This requires planning for future service provision at local and national level. This may be facilitated by prospective data collection using a system similar to the National Joint Registry or National Hip Fracture Database.

**Data Sharing**
Due to information governance rules applicable to HES, no data are available for sharing. HES data is available directly from NHS Digital. Please see: https://digital.nhs.uk/data-and-information/data-tools-and-services/data-services/hospital-episode-statistics/users-uses-and-access-to-hospital-episode-statistics#access-to-data

**Patient and Public Involvement**
This research was done without patient involvement. As an observational study of national level data it was not possible to involve these groups. The suggested next steps of designing services to treat these injuries appropriately would benefit from patient and public involvement.

**Author Contributions**
AB devised the study, extracted and analysed the data, wrote the manuscript, reviewed the manuscript.
RG devised the study, reviewed the data, wrote the manuscript, reviewed the manuscript.
SMW reviewed the data, wrote the manuscript, reviewed the manuscript.
HWJ reviewed the data, wrote the manuscript, reviewed the manuscript.
PA reviewed the data, wrote the manuscript, reviewed the manuscript.
IM reviewed the data, wrote the manuscript, reviewed the manuscript.
EAHC devised the study, reviewed the data, reviewed the manuscript.
HW reviewed the data, wrote the manuscript, reviewed the manuscript.
BMD devised the study, reviewed the data, wrote the manuscript, reviewed the manuscript, and guarantees the paper.

**Acknowledgements**
We would like to thank Ketan Patel for his assistance regarding the use of the code M96.6.

**Funding**
AB and PA work at the Dr Foster Unit, an academic unit in the Department of Primary Care and Public Health, Imperial College London. The unit receives research funding from Dr Foster Intelligence, an independent health service research organization (a wholly owned subsidiary of Telstra). The Dr Foster Unit at Imperial is affiliated with the National Institute of Health Research (NIHR) Imperial Patient Safety Translational Research Centre. The NIHR Imperial Patient Safety Translational Centre is a partnership between the Imperial College Healthcare NHS Trust and Imperial College London. The Department of Primary

For peer review only

Care and Public Health at Imperial College London is grateful for support from the NW London NIHR Collaboration for Leadership in Applied Health Research & Care (CLAHRC) and the Imperial NIHR Biomedical Research Centre.

BMD is funded by an NIHR Clinical Lecturer position.

Funders did not have any input to study conception, design, analysis or manuscript writing.

**Competing Interest Statement**

AB reports grants from Dr Foster, grants from Medtronic, outside the submitted work
RG has nothing to disclose.
HWJ has received payment in past for educational and consultation services to DePuy Synthes and Stryker. Institutional grants received from DePuy Synthes for International Surgeon visitations. Trustee of The John Charnley Trust.
EAHC has nothing to disclose.
PA has nothing to disclose.
BMD is funded by a National Institute of Health Research Clinical Lectureship appointment.
HW has nothing to disclose.
IM has nothing to disclose.
SW has nothing to disclose.

**Disclaimer**

This publication presents independent research funded by the National Institute for Health Research (NIHR). The views expressed are those of the author(s) and not necessarily those of the NHS, the NIHR or the Department of Health and Social Care.

**References**

1. Learmonth I D, Young C, Rorabeck C. The operation of the century; total hip replacement. Lancet 2007;**307**:1508–19.
2. National Joint Registry 16th Report. Available from URL: https://reports.njrcentre.org.uk/Portals/0/PDFdownloads/NJR%2016th%20Annual%20Report%202019.pdf (accessed 20/12/2019)
3. National Hip Fracture Database Annual Report 2019. Available from URL: https://www.nhfd.co.uk/files/2019ReportFiles/NHFD_2019_Annual_Report.pdf (accessed 20/12/2019)
4. Office for National Statistics 2015. Available from URL: https://www.ons.gov.uk/peoplepopulationandcommunity/birthsdeathsandmarriages/lifeexpectancies/bulletins/lifeexpectancyatbirthandatage65bylocalareasinenglandandwales/2015-11-04 (accessed 09/08/2018)
5. Clegg A, Young J, Iliffe S, Rikkert MO, Rockwood K. Frailty in elderly people. Lancet 2013;**381**:752–762.
6. Clinical Classifications Service – Health & Social Care Information Centre. Coding Clinic Version 4.1. March 2015.
7. NHS Digital. Hospital Episode Statistics. Available from URL: https://digital.nhs.uk/data-and-information/data-tools-and-services/data-services/hospital-episode-statistics (accessed Oct 11 2018)
8. International Classification of Diseases ICD-10. Available from URL: http://apps.who.int/classifications/icd10/browse/2010/en (accessed 10/08/2018)


For peer review only - http://bmjopen.bmj.com/site/about/guidelines.xhtml

9. Carstairs V, Morris R. Deprivation and Health in Scotland. Aberdeen University Press, 1991.
10. Burns EM, Rigby E, Mamidanna R, Bottle A, Aylin P, Ziprin P, Faiz OD. Systematic review of discharge coding accuracy. J Public Health (Oxf) 2012;**34(1)**:138–48.
11. Leal J, Gray AM, Prieto-Alhambra D, Arden NK, Cooper C, Jarvaid MK, Judge A. Impact of hip fracture on hospital care costs: a population-based study. Osteoporosis Int 2016;**27**:549–558.
12. Moppett I. Hip fractures: are we asking the right questions? Age Ageing 2018;**47(5)**:633–4.
13. Reich MS, Duong M, Breslin MA, Nguyen MP, Vallier HA. Perioperative Considerations when treating isolated periprosthetic distal femur fractures. The Iowa Orthopedic Journal 2017;**37**:41–45.
14. Capone A, Congia S, Civinini R, Marongiu G. Periprosthetic fractures: epidemiology and current treatment. Clin Cases Miner Bone Metab. 2017;**14(2)**:189–96.
15. Griffiths EJ, Cash DJW, Kalra S, Hopgood PJ. Time to surgery and 30-day morbidity and mortality of periprosthetic hip fractures. Injury. 2013 Dec 1;44(12):1949–52.
16. Jones AR, Williams T, Paringe V, White SP. The economic impact of surgically treated peri-prosthetic hip fractures on a university teaching hospital in Wales 7.5-year study. Injury 2016;47(2):428–31.

I, the Submitting Author has the right to grant and does grant on behalf of all authors of the Work (as defined in the below author licence), an exclusive licence and/or a non-exclusive licence for contributions from authors who are: i) UK Crown employees; ii) where BMJ has agreed a CC-BY licence shall apply, and/or iii) in accordance with the terms applicable for US Federal Government officers or employees acting as part of their official duties; on a worldwide, perpetual, irrevocable, royalty-free basis to BMJ Publishing Group Ltd ("BMJ") its licensees and where the relevant Journal is co-owned by BMJ to the co-owners of the Journal, to publish the Work in BMJ Open and any other BMJ products and to exploit all rights, as set out in our licence.

The Submitting Author accepts and understands that any supply made under these terms is made by BMJ to the Submitting Author unless you are acting as an employee on behalf of your employer or a postgraduate student of an affiliated institution which is paying any applicable article publishing charge ("APC") for Open Access articles. Where the Submitting Author wishes to make the Work available on an Open Access basis (and intends to pay the relevant APC), the terms of reuse of such Open Access shall be governed by a Creative Commons licence – details of these licences and which Creative Commons licence will apply to this Work are set out in our licence referred to above.



For peer review only - http://bmjopen.bmj.com/site/about/guidelines.xhtml

**Figure legends**

Figure 1. Number of admissions by month from April 2013 to December 2018, with elective and emergency admissions shown separately

Figure 2. Acute hospital length of stay by age and gender

Figure 3. Acute hospital length of stay by operative management

Figure 4. Kaplan Meier Survival Curve by Operative Status

For peer review only



For peer review only - http://bmjopen.bmj.com/site/about/guidelines.xhtml

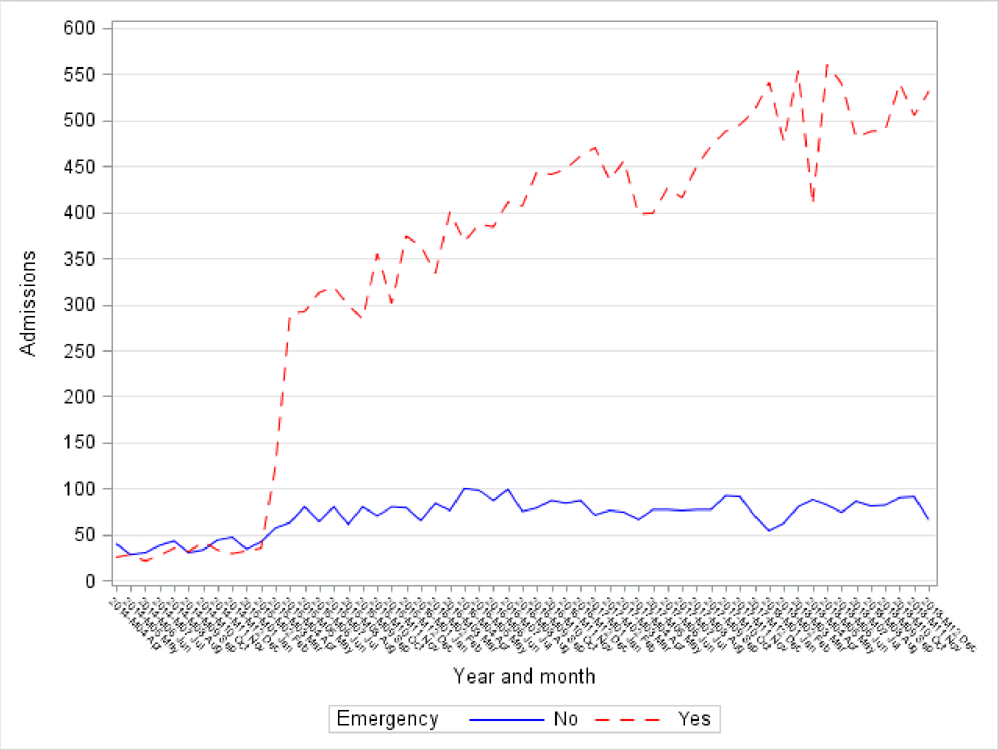


Figure 1. Number of admissions by month from April 2013 to December 2018, with elective and emergency admissions shown separately

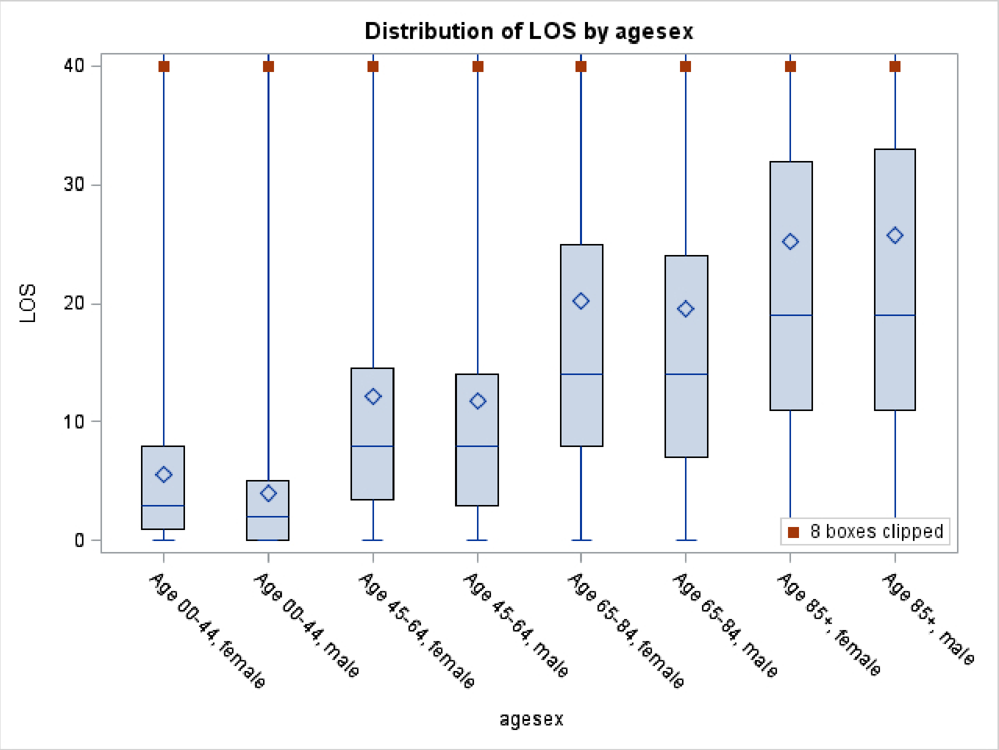


Figure 2. Acute hospital length of stay by age and gender

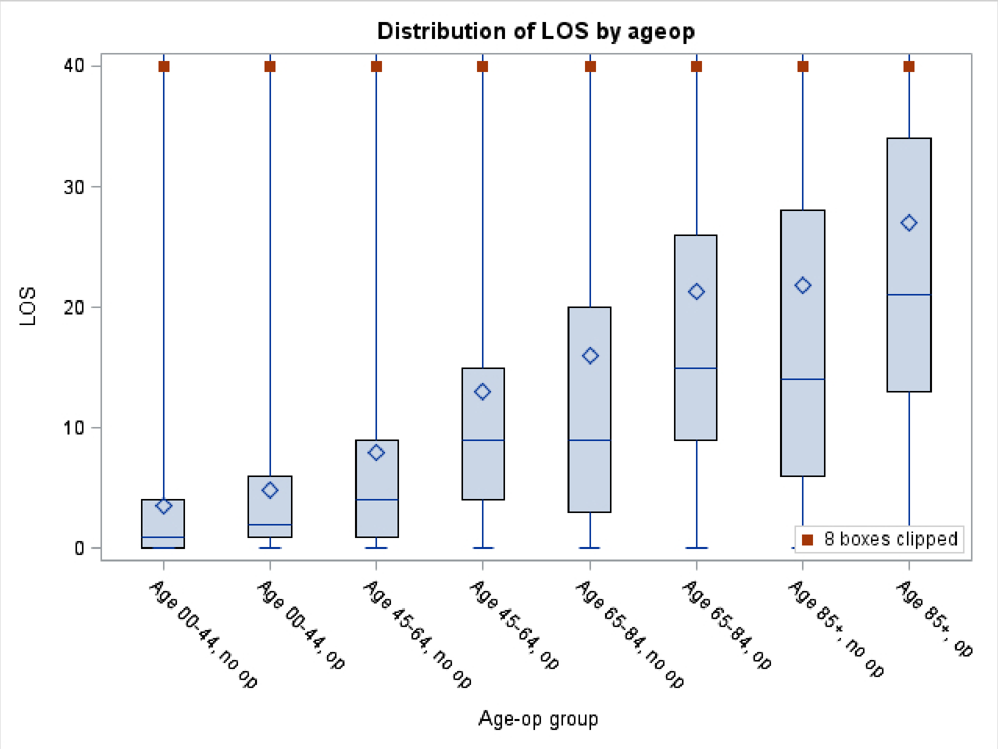


Figure 3. Acute hospital length of stay by operative management

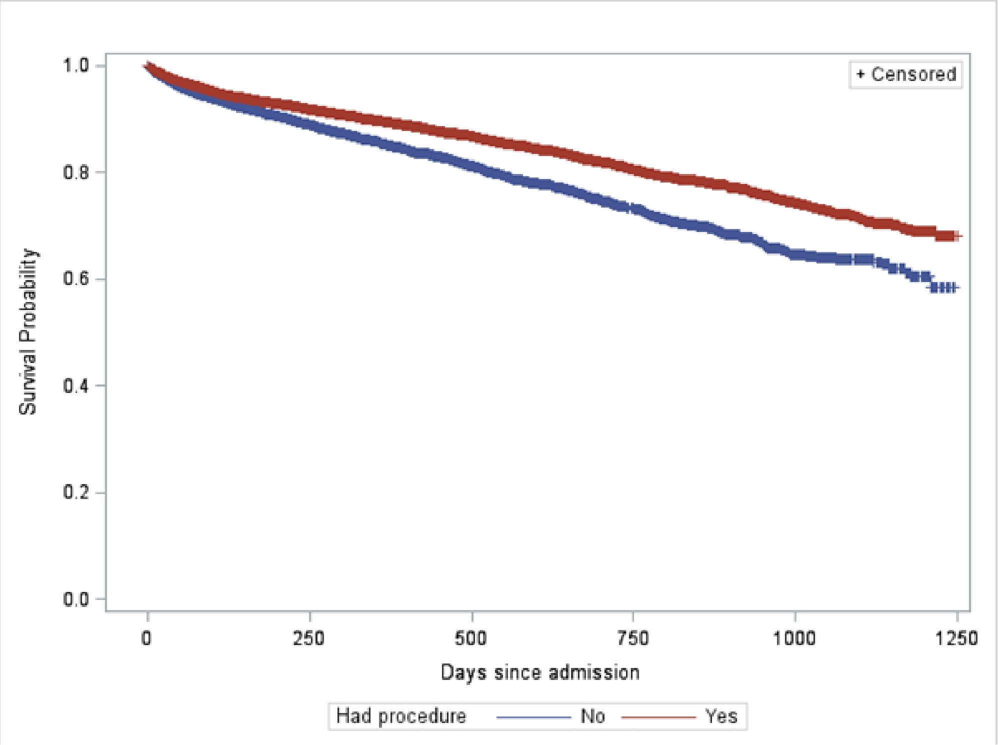


Figure 4. Kaplan Meier Survival Curve by Operative Status

# Supplementary Data - Periprosthetic fractures – the next fragility fracture epidemic? A national observational study

**Supplementary Table 1. The 20 most commonly performed procedures by four-character OPCS code.**

| Op code | Description | Number | Proportion of operations (%) |
|---|---|---|---|
| W201 | Primary open reduction of fracture of long bone and extramedullary fixation using plate NEC | 5004 | 14.8 |
| W283 | Removal of internal fixation from bone NEC | 1728 | 5.1 |
| W202 | Primary open reduction of fracture of long bone and extramedullary fixation using cerclage | 1122 | 3.3 |
| W373 | Revision of total prosthetic replacement of hip joint using cement | 843 | 2.5 |
| W383 | Revision of total prosthetic replacement of hip joint not using cement | 633 | 1.9 |
| W384 | Revision of one component of total prosthetic replacement of hip joint not using cement | 617 | 1.8 |
| W192 | Primary open reduction of fracture of long bone and fixation using rigid nail NEC | 591 | 1.7 |
| W382 | Conversion to total prosthetic replacement of hip joint not using cement | 588 | 1.7 |
| W370 | Conversion from previous cemented total prosthetic replacement of hip joint | 585 | 1.7 |
| W403 | Revision of total prosthetic replacement of knee joint using cement | 582 | 1.7 |
| W374 | Revision of one component of total prosthetic replacement of hip joint using cement | 501 | 1.5 |
| W901 | Aspiration of joint | 495 | 1.5 |
| W281 | Application of internal fixation to bone NEC | 448 | 1.3 |
| W232 | Secondary open reduction of fracture of bone and extramedullary fixation HFQ | 420 | 1.2 |
| W322 | Allograft of bone NEC | 389 | 1.1 |
| W246 | Closed reduction of fracture of bone and fixation using nail or screw | 333 | 1.0 |
| W191 | Primary open reduction of fracture of neck of femur and open fixation using pin and plate | 310 | 0.9 |
| W242 | Closed reduction of fracture of long bone and rigid internal fixation NEC | 300 | 0.9 |
| W198 | Other specified primary open reduction of fracture of bone and intramedullary fixation | 298 | 0.9 |
| W381 | Primary total prosthetic replacement of hip joint not using cement | 258 | 0.8 |
| other | All other main procedures combined | 33922 | 53.7 |

NEC = not elsewhere classified

For peer review only

**Supplementary Table 2. Operative rate according to age, gender and method of admission**

| Factor | Elective admissions with operation | Emergency admissions with operation | % of all elective admissions with operation | % of all emergency admissions with operation |
|---|---|---|---|---|
| Age 0-44, female | 65 | 98 | 95.6 | 66.7 |
| Age 0-44, male | 146 | 225 | 88.5 | 74.3 |
| Age 45-64, female | 229 | 636 | 95.0 | 76.9 |
| Age 45-64, male | 164 | 476 | 92.1 | 77.5 |
| Age 65-84, female | 552 | 4058 | 82.5 | 73.6 |
| Age 65-84, male | 304 | 2298 | 85.4 | 74.9 |
| Age 85+, female | 140 | 3249 | 64.5 | 67.0 |
| Age 85+, male | 56 | 1092 | 62.2 | 69.0 |
| Total | 1656 | 12132 | 83.5 | 71.8 |

**Supplementary Table 3. Length of stay in nights according to major operation status**

| Patient group | LOS type | N | Lower quartile | Median | Mean | Upper quartile |
|---|---|---|---|---|---|---|
| All | Acute stay | 18888 | 7 | 14 | 20.5 | 26 |
| All | Total | 18888 | 8 | 17 | 25.0 | 33 |
| No major operation | Acute stay | 5100 | 4 | 10 | 17.6 | 22 |
| No major operation | Total | 5100 | 4 | 11 | 19.8 | 26 |
| Major operation | Acute stay | 13788 | 9 | 16 | 21.6 | 27 |
| Major operation | Total | 13788 | 10 | 19 | 26.9 | 35 |

"Acute stay" means the stay at the first (acute) hospital; "Total" covers the whole admission, including inter-hospital transfers

**Supplementary Table 4. Length of stay in nights by age and gender**

| Patient group | LOS type | N | Lower quartile | Median | Mean | Upper quartile |
|---|---|---|---|---|---|---|
| Age 0-44, female | Acute stay | 215 | 1 | 3 | 5.6 | 8 |
| | Total | 215 | 1 | 3 | 6.8 | 9 |
| Age 0-44, male | Acute stay | 468 | 0 | 2 | 4.0 | 5 |
| | Total | 468 | 0 | 2 | 4.7 | 5 |
| Age 45-64, female | Acute stay | 1068 | 3.5 | 8 | 12.1 | 14.5 |
| | Total | 1068 | 4 | 9 | 14.6 | 17 |
| Age 45-64, male | Acute stay | 792 | 3 | 8 | 11.8 | 14 |
| | Total | 792 | 3 | 8 | 13.5 | 15 |
| Age 65-84, female | Acute stay | 6186 | 8 | 14 | 20.2 | 25 |
| | Total | 6186 | 9 | 17 | 24.6 | 31 |
| Age 65-84, male | Acute stay | 3424 | 7 | 14 | 19.5 | 24 |
| | Total | 3424 | 8 | 16 | 23.9 | 30 |
| Age 85+, female | Acute stay | 5063 | 11 | 19 | 25.2 | 32 |
| | Total | 5063 | 12 | 23 | 31.0 | 41 |
| Age 85+, male | Acute stay | 1672 | 11 | 19 | 25.7 | 33 |
| | Total | 1672 | 13 | 23 | 31.4 | 43 |

"Acute stay" means the stay at the first (acute) hospital; "Total" covers the whole admission, including inter-hospital transfers


For peer review only - http://bmjopen.bmj.com/site/about/guidelines.xhtml

**Supplementary Table 5. Length of stay in nights by age, gender and operative status**

| Patient group | LOS type | Operated | N | Lower quartile | Median | Mean | Upper quartile |
|---|---|---|---|---|---|---|---|
| Age 0-44, female | Acute stay | No | 52 | 1 | 2 | 4.0 | 5 |
| | | Yes | 52 | 1 | 3 | 6.1 | 8 |
| | Total | No | 163 | 1 | 2 | 4.4 | 6 |
| | | Yes | 163 | 1 | 4 | 7.6 | 10 |
| Age 0-44, male | Acute stay | No | 97 | 0 | 1 | 3.2 | 3 |
| | | Yes | 97 | 1 | 2 | 4.3 | 5 |
| | Total | No | 371 | 0 | 1 | 3.6 | 3 |
| | | Yes | 371 | 1 | 2 | 5.0 | 6 |
| Age 45-64, female | Acute stay | No | 203 | 1 | 4 | 8.3 | 11 |
| | | Yes | 203 | 4 | 9 | 13.0 | 15 |
| | Total | No | 865 | 1 | 4 | 10.2 | 13 |
| | | Yes | 865 | 5 | 10 | 15.6 | 18 |
| Age 45-64, male | Acute stay | No | 152 | 1 | 3 | 7.4 | 7.5 |
| | | Yes | 152 | 4 | 9 | 12.8 | 15 |
| | Total | No | 640 | 1 | 4 | 8.9 | 8 |
| | | Yes | 640 | 4 | 10 | 14.5 | 17 |
| Age 65-84, female | Acute stay | No | 1576 | 3 | 10 | 16.4 | 20 |
| | | Yes | 1576 | 9 | 16 | 21.4 | 26 |
| | Total | No | 4610 | 3 | 10 | 17.9 | 23 |
| | | Yes | 4610 | 11 | 19 | 26.8 | 33 |
| Age 65-84, male | Acute stay | No | 822 | 2 | 8 | 15.1 | 19 |
| | | Yes | 822 | 9 | 15 | 20.9 | 25 |
| | Total | No | 2602 | 2 | 8 | 16.5 | 21 |
| | | Yes | 2602 | 10 | 18 | 26.1 | 32 |
| Age 85+, female | Acute stay | No | 1674 | 6 | 14 | 21.8 | 28 |
| | | Yes | 1674 | 13 | 20 | 26.9 | 34 |
| | Total | No | 3389 | 7 | 16 | 25.3 | 33 |
| | | Yes | 3389 | 15 | 26 | 33.7 | 44 |
| Age 85+, male | Acute stay | No | 524 | 7 | 14 | 21.9 | 27 |
| | | Yes | 524 | 13 | 21 | 27.5 | 34 |
| | Total | No | 1148 | 7 | 16 | 24.9 | 34 |
| | | Yes | 1148 | 16 | 26 | 34.3 | 46 |

"Acute stay" means the stay at the first (acute) hospital; "Total" covers the whole admission, including inter-hospital transfers

For peer review only

**Supplementary Figure 1. Kaplan Meier Survival Curve by sex**

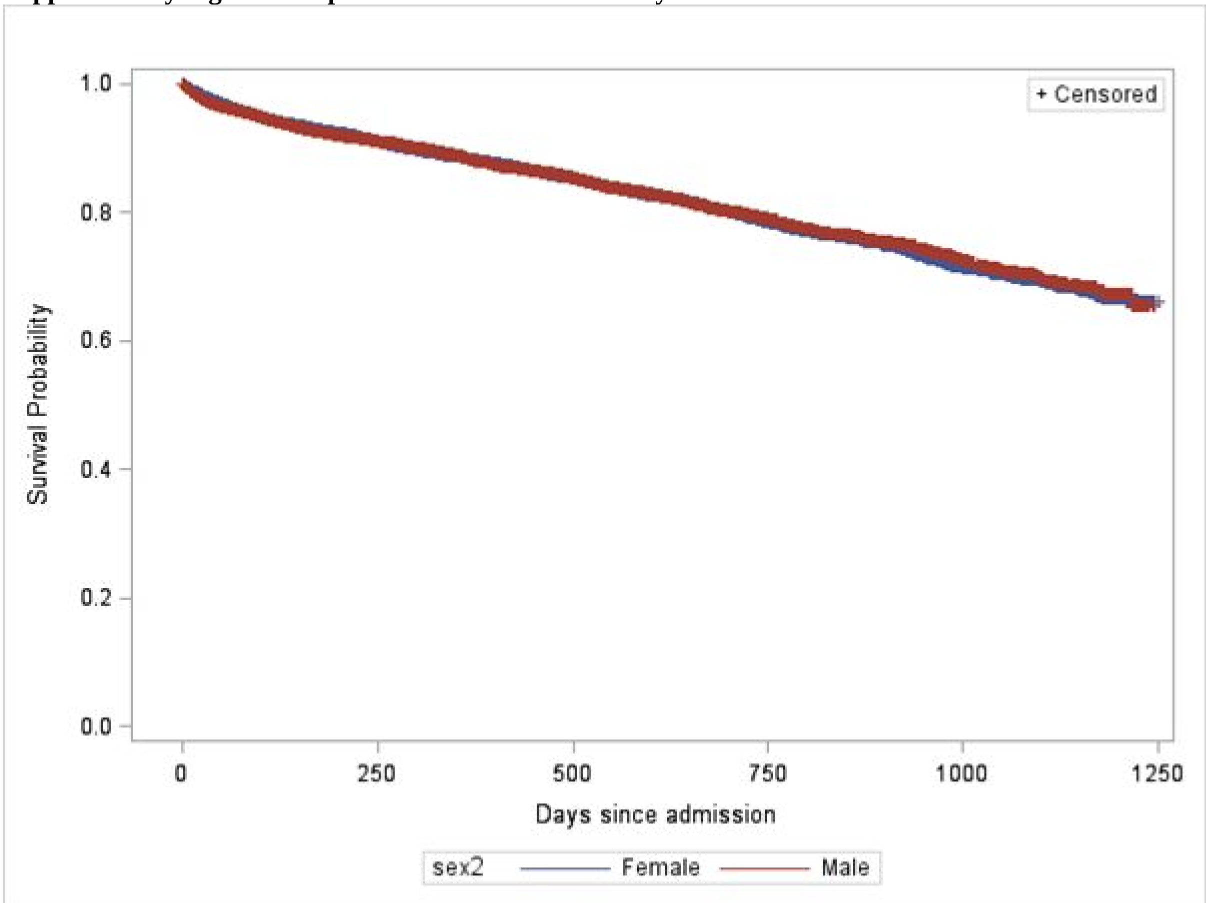




For peer review only - http://bmjopen.bmj.com/site/about/guidelines.xhtml

**Supplementary Figure 2. Kaplan Meier Survival Curve by age range**

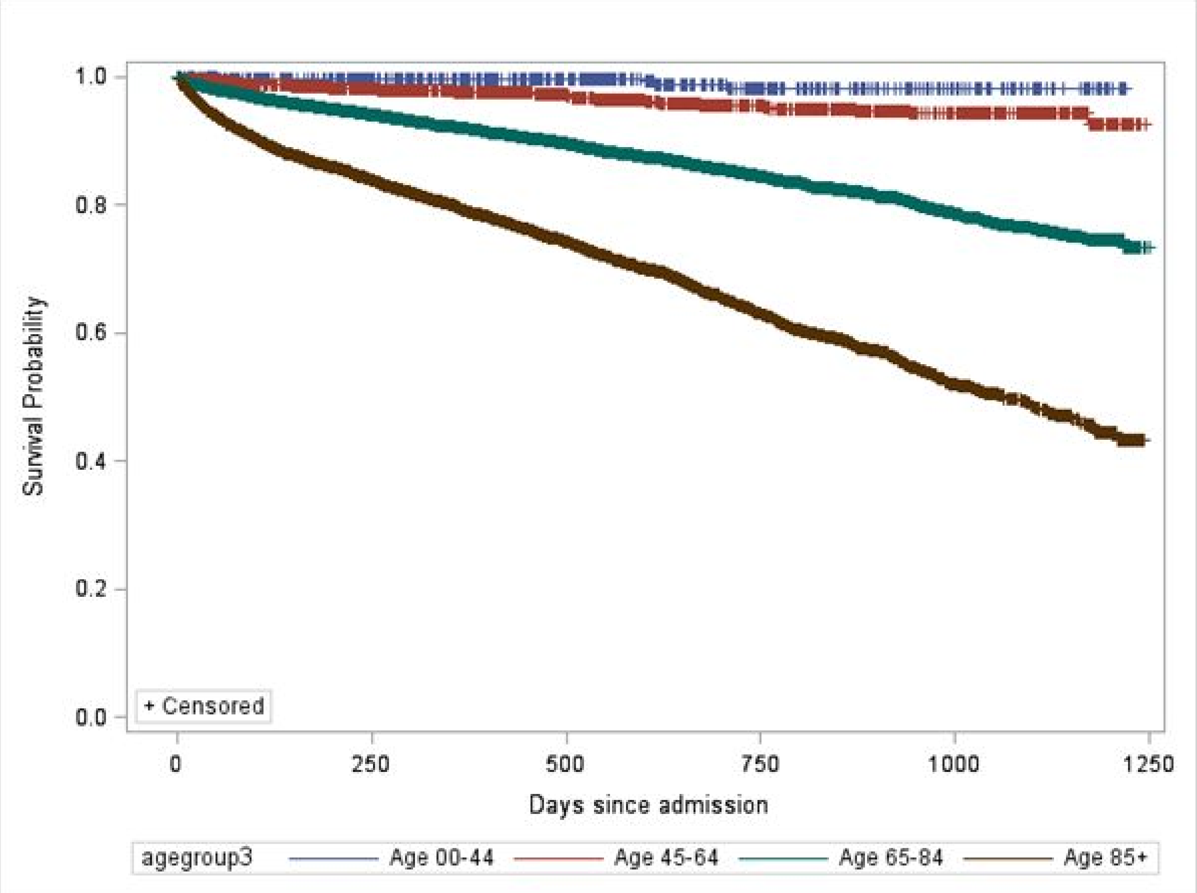




For peer review only - http://bmjopen.bmj.com/site/about/guidelines.xhtml

STROBE Statement—checklist of items that should be included in reports of observational studies

| | Item No | Recommendation | Page No |
|---|---|---|---|
| **Title and abstract** | 1 | (*a*) Indicate the study's design with a commonly used term in the title or the abstract | 1 |
| | | (*b*) Provide in the abstract an informative and balanced summary of what was done and what was found | 2 |
| **Introduction** | | | |
| Background/rationale | 2 | Explain the scientific background and rationale for the investigation being reported | 3 |
| Objectives | 3 | State specific objectives, including any prespecified hypotheses | 3 |
| **Methods** | | | |
| Study design | 4 | Present key elements of study design early in the paper | 3 |
| Setting | 5 | Describe the setting, locations, and relevant dates, including periods of recruitment, exposure, follow-up, and data collection | 3-4 |
| Participants | 6 | (*a*) *Cohort study*—Give the eligibility criteria, and the sources and methods of selection of participants. Describe methods of follow-up *Case-control study*—Give the eligibility criteria, and the sources and methods of case ascertainment and control selection. Give the rationale for the choice of cases and controls *Cross-sectional study*—Give the eligibility criteria, and the sources and methods of selection of participants | 3-4 |
| | | (*b*) *Cohort study*—For matched studies, give matching criteria and number of exposed and unexposed *Case-control study*—For matched studies, give matching criteria and the number of controls per case | n/a |
| Variables | 7 | Clearly define all outcomes, exposures, predictors, potential confounders, and effect modifiers. Give diagnostic criteria, if applicable | 4 |
| Data sources/ measurement | 8* | For each variable of interest, give sources of data and details of methods of assessment (measurement). Describe comparability of assessment methods if there is more than one group | 3-4 |
| Bias | 9 | Describe any efforts to address potential sources of bias | |
| Study size | 10 | Explain how the study size was arrived at | 4 |
| Quantitative variables | 11 | Explain how quantitative variables were handled in the analyses. If applicable, describe which groupings were chosen and why | |
| Statistical methods | 12 | (*a*) Describe all statistical methods, including those used to control for confounding | 4 |
| | | (*b*) Describe any methods used to examine subgroups and interactions | 4 |
| | | (*c*) Explain how missing data were addressed | n/a |
| | | (*d*) *Cohort study*—If applicable, explain how loss to follow-up was addressed *Case-control study*—If applicable, explain how matching of cases and controls was addressed *Cross-sectional study*—If applicable, describe analytical methods taking account of sampling strategy | |
| | | (*e*) Describe any sensitivity analyses | 4 |

Continued on next page


For peer review only - http://bmjopen.bmj.com/site/about/guidelines.xhtml

| | | | |
|---|---|---|---|
| **Results** | | | |
| Participants | 13* | (a) Report numbers of individuals at each stage of study—eg numbers potentially eligible, examined for eligibility, confirmed eligible, included in the study, completing follow-up, and analysed | 4 |
| | | (b) Give reasons for non-participation at each stage | n/a |
| | | (c) Consider use of a flow diagram | |
| Descriptive data | 14* | (a) Give characteristics of study participants (eg demographic, clinical, social) and information on exposures and potential confounders | 5 (and table 3) |
| | | (b) Indicate number of participants with missing data for each variable of interest | |
| | | (c) *Cohort study*—Summarise follow-up time (eg, average and total amount) | |
| Outcome data | 15* | *Cohort study*—Report numbers of outcome events or summary measures over time | 5-6 |
| | | *Case-control study*—Report numbers in each exposure category, or summary measures of exposure | |
| | | *Cross-sectional study*—Report numbers of outcome events or summary measures | |
| Main results | 16 | (*a*) Give unadjusted estimates and, if applicable, confounder-adjusted estimates and their precision (eg, 95% confidence interval). Make clear which confounders were adjusted for and why they were included | |
| | | (*b*) Report category boundaries when continuous variables were categorized | Table 4 |
| | | (*c*) If relevant, consider translating estimates of relative risk into absolute risk for a meaningful time period | |
| Other analyses | 17 | Report other analyses done—eg analyses of subgroups and interactions, and sensitivity analyses | 6-8 |
| **Discussion** | | | |
| Key results | 18 | Summarise key results with reference to study objectives | 9-10 |
| Limitations | 19 | Discuss limitations of the study, taking into account sources of potential bias or imprecision. Discuss both direction and magnitude of any potential bias | 11 |
| Interpretation | 20 | Give a cautious overall interpretation of results considering objectives, limitations, multiplicity of analyses, results from similar studies, and other relevant evidence | 11 |
| Generalisability | 21 | Discuss the generalisability (external validity) of the study results | 11-12 |
| **Other information** | | | |
| Funding | 22 | Give the source of funding and the role of the funders for the present study and, if applicable, for the original study on which the present article is based | 12 |

*Give information separately for cases and controls in case-control studies and, if applicable, for exposed and unexposed groups in cohort and cross-sectional studies.

**Note:** An Explanation and Elaboration article discusses each checklist item and gives methodological background and published examples of transparent reporting. The STROBE checklist is best used in conjunction with this article (freely available on the Web sites of PLoS Medicine at http://www.plosmedicine.org/, Annals of Internal Medicine at http://www.annals.org/, and Epidemiology at http://www.epidem.com/). Information on the STROBE Initiative is available at www.strobe-statement.org.


For peer review only - http://bmjopen.bmj.com/site/about/guidelines.xhtml

# Periprosthetic fractures – the next fragility fracture epidemic? A national observational study

| | |
|---|---|
| Journal: | *BMJ Open* |
| Manuscript ID | bmjopen-2020-042371.R1 |
| Article Type: | Original research |
| Date Submitted by the Author: | 05-Oct-2020 |
| Complete List of Authors: | Bottle, Alex; Imperial College, Primary Care and Public Health<br>Griffiths, Richard; Peterborough City Hospital, Anaesthesia<br>White, Stuart; Royal Sussex County Hospital,<br>Wynn-Jones, Henry; Wrightington Hospital, The Centre for Hip Surgery<br>Aylin, Paul; Imperial College London, Primary Care and Public Health<br>Moppett, Iain; University of Nottingham, Anaesthesia & Critical Care<br>Chowdhury, Emyr; North West Anglia NHS Foundation Trust<br>Wilson, Helen; Royal Surrey County Hospital NHS Foundation Trust, Department of Geriatric Medicine<br>Davies, Benjamin; University of Cambridge, Department of Surgery |
| <b>Primary Subject Heading</b>: | Surgery |
| Secondary Subject Heading: | Epidemiology, Geriatric medicine |
| Keywords: | Adult orthopaedics < ORTHOPAEDIC & TRAUMA SURGERY, EPIDEMIOLOGY, GERIATRIC MEDICINE |
| | |

SCHOLARONE™
Manuscripts

*I, the Submitting Author has the right to grant and does grant on behalf of all authors of the Work (as defined in the below author licence), an exclusive licence and/or a non-exclusive licence for contributions from authors who are: i) UK Crown employees; ii) where BMJ has agreed a CC-BY licence shall apply, and/or iii) in accordance with the terms applicable for US Federal Government officers or employees acting as part of their official duties; on a worldwide, perpetual, irrevocable, royalty-free basis to BMJ Publishing Group Ltd ("BMJ") its licensees and where the relevant Journal is co-owned by BMJ to the co-owners of the Journal, to publish the Work in this journal and any other BMJ products and to exploit all rights, as set out in our licence.*

*The Submitting Author accepts and understands that any supply made under these terms is made by BMJ to the Submitting Author unless you are acting as an employee on behalf of your employer or a postgraduate student of an affiliated institution which is paying any applicable article publishing charge ("APC") for Open Access articles. Where the Submitting Author wishes to make the Work available on an Open Access basis (and intends to pay the relevant APC), the terms of reuse of such Open Access shall be governed by a Creative Commons licence – details of these licences and which Creative Commons licence will apply to this Work are set out in our licence referred to above.*

*Other than as permitted in any relevant BMJ Author's Self Archiving Policies, I confirm this Work has not been accepted for publication elsewhere, is not being considered for publication elsewhere and does not duplicate material already published. I confirm all authors consent to publication of this Work and authorise the granting of this licence.*

For peer review only

Title:
# Periprosthetic fractures – the next fragility fracture epidemic? A national observational study

Authors:
Alex Bottle[1] (robert.bottle@imperial.ac.uk)
Richard Griffiths[2] (richardgriffiths1@nhs.net)
Stuart M White[3] (igasbest@hotmail.com)
Henry Wynn-Jones[4] (henrywynnjones@gmail.com)
Paul Aylin[1] (p.aylin@imperial.ac.uk)
Iain Moppett[5] (iain.moppett@nottingham.ac.uk)
Emyr A H Chowdhury[6] (emyr.chowdhury@nhs.net)
Helen Wilson[7] (hwilson6@nhs.net)
Benjamin M Davies[8] (bmd35@cam.ac.uk)

Affiliations:
1 – Dr Foster Unit, Dept of Primary Care and Public Health, School of Public Health, Imperial College London, Reynolds Building, St Dunstan's Road, London, United Kingdom
2 – Department of Anaesthesia, Peterborough City Hospital, Peterborough, United Kingdom
3 – Haywards Heath Hip Fracture Unit, Princess Royal Hospital, Brighton and Sussex University Hospitals NHS Trust, Haywards Heath, United Kingdom
4 – The Centre for Hip Surgery, Wrightington Hospital, Hall Lane, Appley Bridge, Lancashire, United Kingdom
5 – Anaesthesia and Critical Care, Division of Clinical Neuroscience, The University of Nottingham, Queen's Medical Centre, Nottingham, United Kingdom
6 – Department of Trauma and Orthopaedics, Peterborough City Hospital, Peterborough, United Kingdom
7 – Department of General Medicine, Stroke and Care of the Elderly, Royal Surrey County Hospital, Guildford, United Kingdom
8 – Division of Trauma and Orthopaedics, University of Cambridge, Addenbrooke's Hospital, Cambridge, United Kingdom

Corresponding Author:
Mr Benjamin Davies, Division of Trauma and Orthopaedics, University of Cambridge, Box 180, Addenbrooke's Hospital, Hills Road, Cambridge, CB2 2QQ, United Kingdom
Email: bmd35@cam.ac.uk
Telephone: 07974 192502

Keywords:
Periprosthetic fracture
Fragility fracture
Trauma
Hospital Episode Statistics

Word Count: 3,048

For peer review only


For peer review only - http://bmjopen.bmj.com/site/about/guidelines.xhtml

Abstract:

Objectives –

Periprosthetic fractures have considerable clinical implications for patients and financial implications for healthcare systems. This study aims to determine the burden of periprosthetic fractures of the lower and upper limbs in England and identify any factors associated with differences in treatment and outcome.

Design –

A national, observational study

Setting –

England

Participants –

All individuals admitted to hospital with peri-prosthetic factures between 01/04/2015 and 31/12/2018.

Primary and Secondary Outcome Measures –

Mortality, length of stay, change in rate of admissions.

Methods –

We analysed Hospital Episode Statistics (HES) data using the ICD-10 code M96.6 ("Fracture of bone following insertion of orthopaedic implant, joint prosthesis, or bone plate") to identify periprosthetic fractures recorded between April 2013 and December 2018. We determined the demographics, procedures performed, mortality rates, and discharge destinations. Patient characteristics associated with having a procedure during the index admission were estimated using logistic regression. The annual rate of increase in admissions was estimated using Poisson regression.

Results –

Between 01/04/2015 and 31/12/2018, there were 13,565 patients who had 18,888 admissions (89.5% emergency) with M96.6 in the primary diagnosis field. There was a 13% year-on-year increase in admissions for periprosthetic fracture in England during that period. Older people, people living in deprived areas, and those with heart failure or neurological disorders were less likely to receive an operation. 14.4% of patients did not return home after hospital discharge. The overall in-patient mortality was 4.3% and total 30-day mortality 3.3%.

Conclusions –

The clinical and operational burden of periprosthetic fractures is considerable and increasing rapidly. We suggest that the management of people with periprosthetic fractures should be undertaken and funded in a similar manner to that successfully employed for people sustaining hip fractures, utilising national standards and data collection to monitor and improve performance.

**Article Summary - strengths and limitations of this study**

- This is the first study to use ICD-10 code M96.6 to identify trends in peri-prosthetic fracture rates.
- This data can be used to help develop specific management pathways and allocate resources.
- Use of HES data is limited by potential inaccuracies in coding.
- The study data does not include functional outcomes which would provide useful information.

For peer review only

## Introduction

Since the successful introduction of total hip replacement in the 1960s, joint replacement has revolutionised the treatment of severely arthritic joints.[1] Joint replacement prostheses are available for all the major joints of the upper and lower limbs. In 2018, approximately 200,000 arthroplasty procedures were recorded in the 16th Annual report of the National Joint Registry (NJR) for England and Wales.[2] Between the advent of the registry, on 1st April 2003, and 31st December 2018, 2.75 million joint replacement procedures have been recorded. In addition, 28,684 femoral hemiarthroplasties were performed in 2018 for proximal femoral fractures.[3] The increasing life expectancy in the UK[4], combined with the effects of chronic disease and frailty[5] lead to an increased likelihood of sustaining a fracture due to osteoporosis and an increased incidence of falls. Fractures can also occur around joint replacements, and these are termed periprosthetic fractures. Periprosthetic fractures may require either fixation or revision of the implant. Surgery to treat such fractures is often complex and/or prolonged, requiring specialist input from a multidisciplinary team.

There has been no comprehensive assessment of the burden of these injuries upon the healthcare system in England. The NJR collects data on revision arthroplasty and identifies those performed for peri-prosthetic fracture. Fractures treated without the need to revise the implant are not recorded at present in the NJR. The alternative sources of data are the UK's four national hospital administrative databases: Hospital Episodes Statistics (HES) for England, and similar systems for each of the other three countries. Clinical coders use ICD-10 and UK-specific OPCS coding systems to record diagnosis and procedure information respectively. Owing to the complex classification of fractures and different techniques to treat them, there are many operative codes that could be used. In March 2015, NHS England issued new guidance to clinical coders regarding periprosthetic fractures.[6] The new ICD10 code M96.6 ("Fracture of bone following insertion of orthopaedic implant, joint prosthesis, or bone plate") is designed to capture all patients who have experienced a periprosthetic fracture at any time during an episode of care, regardless of site or operative treatment. It also captures fractures around fixation devices such as plates and intramedullary nails.

The purpose of this observational study is to quantify the burden of these cases on the NHS and identify any trends in the treatment of these conditions which may inform future healthcare resource planning. Using England's national hospital administrative database, HES, we describe the admissions with the new M96.6 diagnosis code, time trends, patient characteristics, operative rate and short-term outcomes. A literature review carried out by the authors did not identify any previous reporting of these national level data.

## Methods

Data

Hospital Episode Statistics (HES)[7] comprises over 125 million admitted patient, outpatient and emergency department records from hospitals within England's National Health Service (NHS) annually. Inpatient records consist of consultant episodes: each episode covers the period during which a patient is the responsibility of a given physician. A single admission at a given hospital may comprise multiple episodes. Episodes were linked into admissions and linkage with inter-hospital transfers creates "superspells"; we will use the term "admission" throughout to refer to superspells. HES provides data on in-hospital mortality and is linked to the national death register. One primary and up to 19 secondary diagnoses are coded using ICD-10;[8] one primary and up to 23 secondary procedures are coded using the UK's own "OPCS" coding system. Area-level socio-economic deprivation status in population-weighted



For peer review only - http://bmjopen.bmj.com/site/about/guidelines.xhtml

quintiles was linked to HES via the patient's postcode. Coding is completed by specialist clinical coders (rather than clinicians) in hospitals from the medical record. Once received centrally the data is cleaned to remove duplicates.

We extracted all admissions from HES with the ICD-10 code M96.6 in the primary diagnosis field (which corresponds to the main problem treated) and discharge dates between April 2013 and December 2018.

Outcome measures

We report the primary procedure performed, length of stay (acute hospital and superspell), final destination on discharge, in-hospital mortality rate and total 30-day mortality rate.

We also report the proportion of admissions with a procedure, excluding scans via the U chapter of OPCS (Diagnostic Imaging, Testing and Rehabilitation), urinary catheterisation, and auxiliary or minor procedures via the X, Y and Z chapters of OPCS (Miscellaneous and Subsidiary Classification of Methods and Sites of Operation).

Statistical analysis

With any coding change, some variation in the uptake rate between hospitals is expected. We tried to separate the uptake of the new code from any genuine underlying change in the number of admissions by dividing hospital trusts into "early adopters" and the rest. "Early adopters" were defined as those with two or more discharges with M96.6 as the primary diagnosis in March or April 2015. Simple Poisson regression models estimated the linear trend in admissions for each group of trusts ("early adopters" and "other") and for all combined. As a sensitivity analysis the modelling was repeated after excluding the first six months of the new coding period, i.e. including only admissions from October 2015 onwards.

Patient characteristics were described for the cohort from April 2015 including: age, gender, method of admission (whether emergency or not), area-level socio-economic deprivation[9] and comorbidity (using the HES ICD-10 secondary diagnosis codes). The proportion of admissions with a procedure coded, as defined above, was noted and the factors associated with a procedure derived from a multiple logistic regression model with age, gender, deprivation and comorbidities as covariates entered at once with no selection or elimination. To determine what factors were associated with increased LOS a multiple logistic regression was performed with the same predictors as for having a procedure; again, all predictors were retained in the model irrespective of their p-value. We performed the usual diagnostics for logistic regression, including checking for collinearity between predictors, goodness of fit and residuals. These were satisfactory. In the absence of an accepted definition of extended LOS in these cases we used the Upper Quartile boundary (26) to define a long LOS. Kaplan-Meier curves were plotted for overall survival since admission.

Ethical Approval

We have approval from the Secretary of State and the Health Research Authority under Regulation 5 of the Health Service (Control of Patient Information) Regulations 2002 to hold confidential data and analyse them for research purposes (CAG ref 15/CAG/0005). We have approval to use them for research and measuring quality of delivery of healthcare, including for this analysis, from the London - South East Ethics Committee (REC ref 15/LO/0824).


For peer review only - http://bmjopen.bmj.com/site/about/guidelines.xhtml

Patient and Public Involvement

This research was done without patient involvement. As an observational study of national level data it was not possible to involve these groups. The suggested next steps of designing services to treat these injuries appropriately would benefit from patient and public involvement.

**Results**

Between 1st April 2013 and 31st December 2018, there were 24,697 admissions with M96.6 recorded in any diagnosis field. After the coding guidance was introduced in March 2015, there were 22,955 such admissions (84.5% classified as emergency) for 16,105 patients. For the 80.3% who had M96.6 in the primary diagnosis field, there were 18,888 admissions (89.5% emergency) for 13,565 patients.

Change in admissions over time

Table 1 gives the number of admissions by year, split by whether M96.6 was in the primary or in the secondary diagnosis fields.

*Table 1: Total numbers of admissions with M96.6 recorded since April 2013*

| Financial year (Apr 1 to Mar 31) | Numbers of admissions with M96.6 as primary diagnosis | Numbers of admissions with M96.6 only as secondary diagnosis |
|---|---|---|
| 2013/4 | 429 | 384 |
| 2014/5 | 514 | 415 |
| 2015/16 | 3832 | 974 |
| 2016/17 | 5232 | 1077 |
| 2017/18 | 5435 | 1123 |
| 2018/19 to Dec 31 2018 | 4389<br>(5852 full year pro rata) | 893<br>(1191 full year pro rata) |
| Total | 19831 | 4866 |

The subsequent analysis is restricted to admissions with M96.6 in the primary diagnosis.

Figure 1 illustrates admissions by month since April 2014 to December 2018, with elective and emergency admissions shown separately. The rapid rise in admissions from April 2015 coincides with the introduction of the M96.6 coding guidance.

<Figure 1 here>

116 out of 141 hospital trusts using the M96.6 code were labelled early adopters. The rate of increase (slope) for the non-early adopters was significantly higher than that for the early adopters (p=0.001 for the interaction term). Taking the early adopters as being the "true" underlying trend, there was a 13% annual rise in M96.6 admissions between April 2015 and December 2018 (Table 2). Excluding the first six months since the coding change did not change the rate of increase significantly.



For peer review only - http://bmjopen.bmj.com/site/about/guidelines.xhtml

*Table 2: Poisson regression showing annual increase in use of M96.6 diagnosis code nationally and split by whether hospitals were early adopters of this code*

| Set of trusts | Relative Risk per month | 95% CI | Relative Risk per year | 95% CI |
|---|---|---|---|---|
| All | 1.011 | 1.010 - 1.012 | 1.14 | 1.13-1.15 |
| Early M96.6 adopters | 1.010 | 1.009 - 1.011 | 1.13 | 1.11-1.14 |
| Non early adopters | 1.016 | 1.013 - 1.018 | 1.21 | 1.17-1.24 |
| All (Oct 2015 onwards only) | 1.009 | 1.008 - 1.010 | 1.11 | 1.10-1.13 |

<u>Patient Characteristics</u>

The majority of admissions were for women (66.4% female, 33.6% male). Table 3 details patient characteristics.

*Table 3: Patient Characteristics*

| Factor | Admissions | Proportion of total admissions (%) |
|---|---|---|
| Age-sex | | |
| Age 0-44, female | 215 | 1.1 |
| Age 0-44, male | 468 | 2.5 |
| Age 45-64, female | 1068 | 5.7 |
| Age 45-64, male | 792 | 4.2 |
| Age 65-84, female | 6186 | 32.8 |
| Age 65-84, male | 3424 | 18.1 |
| Age 85+, female | 5063 | 26.8 |
| Age 85+, male | 1672 | 8.9 |
| Deprivation | | |
| Quintile 1 (least deprived) | 4796 | 25.4 |
| Quintile 2 | 4467 | 23.6 |
| Quintile 3 | 4090 | 21.7 |
| Quintile 4 | 3143 | 16.6 |
| Quintile 5 (most deprived) | 2300 | 12.2 |
| Quintile 6 (not known) | 92 | 0.5 |
| Dementia | 2634 | 13.9 |
| Alcohol misuse | 625 | 3.3 |
| Arrhythmias | 4130 | 21.9 |
| Heart failure | 1445 | 7.7 |
| COPD | 3337 | 17.7 |
| Deficiency anaemia | 493 | 2.6 |
| Depression | 1231 | 6.5 |
| Diabetes mellitus | 2877 | 15.2 |
| Fluid disorders | 1396 | 7.4 |
| Hypertension | 9208 | 48.8 |
| Hypothyroidism | 1804 | 9.6 |
| Liver disease | 229 | 1.2 |
| Cancer with metastasis | 251 | 1.3 |
| Obesity | 784 | 4.2 |


For peer review only - http://bmjopen.bmj.com/site/about/guidelines.xhtml
For peer review only

| | | |
|---|---|---|
| Other neurological conditions | 1204 | 6.4 |
| Paraplegia | 180 | 1 |
| Pulmonary circulatory disorders | 204 | 1.1 |
| PVD | 520 | 2.8 |
| Renal disease | 2189 | 11.6 |
| Rheumatological disorders | 1619 | 8.6 |
| Solid tumours | 650 | 3.4 |
| Valvular disease | 1091 | 5.8 |

Operative rate and main procedures performed

Given that the coding advice only came into force in March 2015, we now restrict the analysis to the 11,019 discharges from April 2015 onwards, again with M96.6 as the primary diagnosis.

Primary open reduction [W201 + W192 + W202 + W191 + W198] was the commonest recorded surgical technique (21.6%); revision of the prosthesis [W373 + W383 + W384 + W374 + W403 + W393 + W404] was performed in 10.4% (the commonest procedures are listed in Supplementary Table 1).

The overall operative rates were 74.7% in 2015/2016, 72.1% in 2016/17 and 74.5% in 2017/2018. For the elective admissions, 83.5% had a procedure performed; for the emergency admissions, 71.8% had a procedure performed, with an overall proportion of 73.0%.

Table 4 gives the operative rate by patient characteristic and the logistic regression results (See Supplementary Table 2 for operative rates by age, gender, and method of admission). Patients of both sexes aged greater than 84 years were significantly less likely to have an operation than the youngest age group. Surgery was also less likely with increasing socio-economic deprivation, Chronic Obstructive Pulmonary Disease (COPD), and neurological disorders. Surgery was more likely in the presence of recorded fluid and electrolyte disturbance, hypertension, and obesity (OR 1.78, 95% CI 1.46-2.17).

*Table 4: Operative rate by patient characteristics and multiple logistic regression results*

| Factor | No operation | Operation | Proportion with operation (%) | Adjusted OR for operation | P value |
|---|---|---|---|---|---|
| Age 00-44, female | 52 | 163 | 75.8 | 1.23 (0.83 to 1.81) | 0.299 |
| Age 00-44, male | 97 | 371 | 79.3 | 1 | |
| Age 45-64, female | 203 | 865 | 81.0 | 1.29 (0.91 to 1.83) | 0.158 |
| Age 45-64, male | 152 | 640 | 80.8 | 1.27 (0.88 to 1.82) | 0.206 |
| Age 65-84, female | 1576 | 4610 | 74.5 | 0.86 (0.62 to 1.19) | 0.371 |
| Age 65-84, male | 822 | 2602 | 76.0 | 0.97 (0.69 to 1.34) | 0.830 |
| Age 85+, female | 1674 | 3389 | 66.9 | 0.6 (0.44 to 0.83) | 0.002 |
| Age 85+, male | 524 | 1148 | 68.7 | 0.67 (0.48 to 0.94) | 0.019 |
| Quintile 1 (least deprived) | 1267 | 3529 | 73.6 | 1 | |



For peer review only - http://bmjopen.bmj.com/site/about/guidelines.xhtml

| | | | | | |
|---|---|---|---|---|---|
| Quintile 2 | 1183 | 3284 | 73.5 | 1.00 (0.91 to 1.10) | 0.998 |
| Quintile 3 | 1082 | 3008 | 73.5 | 0.98 (0.89 to 1.08) | 0.742 |
| Quintile 4 | 882 | 2261 | 71.9 | 0.89 (0.80 to 0.98) | 0.025 |
| Quintile 5 (most deprived) | 655 | 1645 | 71.5 | 0.84 (0.75 to 0.95) | 0.004 |
| Quintile 6 (not known) | 31 | 61 | 66.3 | 0.64 (0.41 to 1.01) | 0.053 |
| Alcohol misuse | 151 | 474 | 75.8 | 0.93 (0.76 to 1.14) | 0.484 |
| Arrhythmias | 1208 | 2922 | 70.8 | 0.96 (0.89 to 1.05) | 0.385 |
| COPD | 879 | 2458 | 73.7 | 0.84 (0.74 to 0.96) | 0.008 |
| Cancer with metastasis | 65 | 186 | 74.1 | 1.02 (0.93 to 1.11) | 0.681 |
| Deficiency anaemia | 150 | 343 | 69.6 | 0.89 (0.73 to 1.08) | 0.229 |
| Dementia | 910 | 1724 | 65.5 | 0.88 (0.77 to 1.00) | 0.055 |
| Depression | 319 | 912 | 74.1 | 1.02 (0.89 to 1.16) | 0.830 |
| Diabetes mellitus | 777 | 2100 | 73.0 | 0.98 (0.89 to 1.07) | 0.613 |
| Fluid disorders | 340 | 1056 | 75.6 | 1.31 (1.15 to 1.49) | <0.001 |
| Heart failure | 466 | 979 | 67.8 | 1.13 (1.06 to 1.21) | 0.0004 |
| Hypertension | 2424 | 6784 | 73.7 | 1.07 (0.95 to 1.19) | 0.264 |
| Hypothyroidism | 483 | 1321 | 73.2 | 0.98 (0.71 to 1.35) | 0.907 |
| Liver disease | 56 | 173 | 75.5 | 1.03 (0.75 to 1.42) | 0.868 |
| Obesity | 125 | 659 | 84.1 | 1.78 (1.46 to 2.17) | <0.001 |
| Other neurological conditions | 362 | 842 | 69.9 | 0.80 (0.70 to 0.91) | 0.001 |
| Peripheral vascular disease | 143 | 377 | 72.5 | 0.87 (0.63 to 1.21) | 0.401 |
| Paraplegia | 54 | 126 | 70.0 | 1.28 (0.92 to 1.79) | 0.145 |
| Pulmonary circulatory disorders | 49 | 155 | 76.0 | 1.00 (0.82 to 1.23) | 0.974 |
| Renal disease | 637 | 1552 | 70.9 | 0.98 (0.88 to 1.09) | 0.695 |
| Rheumatological disorders | 455 | 1164 | 71.9 | 0.90 (0.80 to 1.01) | 0.065 |
| Solid tumours | 182 | 468 | 72.0 | 0.96 (0.79 to 1.17) | 0.699 |
| Valvular disease | 297 | 794 | 72.8 | 1.11 (0.96 to 1.28) | 0.161 |

<ins>Length of Stay</ins>

Median acute hospital LOS was 14 nights (IQR 7-26), and median total hospital LOS was 17 nights (IQR 8-33: Supplementary Table 3). Acute hospital LOS increased with increasing age and was higher for older ages for both sexes and for operative than non-operative management at each age group (p<0.001; Figures 2 and 3 and Supplementary Tables 3, 4 and 5).

<Figure 2 here>

<Figure 3 here>

In multiple logistic regression, increasing age was significantly associated with a longer length of stay. A wide variety of co-morbidities were also associated with a longer LOS, but with a smaller effect than that of age (See Table 5).



For peer review only - http://bmjopen.bmj.com/site/about/guidelines.xhtml

*Table 5: Factors associated with a long LOS (≥26 nights)*

| Factor | OR (95% CI) | P value |
|---|---|---|
| Age 00-44, female | 0.66 (0.21 to 2.12) | 0.489 |
| Age 00-44, male | 1 | |
| Age 45-64, female | 4.41 (1.77 to 10.95) | 0.001 |
| Age 45-64, male | 3.97 (1.58 to 9.95) | 0.003 |
| Age 65-84, female | 11.01 (4.52 to 26.86) | <0.001 |
| Age 65-84, male | 9.95 (4.07 to 24.32) | <0.001 |
| Age 85+, female | 17.93 (7.35 to 43.74) | <0.001 |
| Age 85+, male | 17.00 (6.93 to 41.66) | <0.001 |
| Quintile 1 (least deprived) | 1 | |
| Quintile 2 | 1.03 (0.93 to 1.13) | 0.603 |
| Quintile 3 | 1.02 (0.92 to 1.13) | 0.689 |
| Quintile 4 | 1.09 (0.98 to 1.22) | 0.115 |
| Quintile 5 (most deprived) | 1.15 (1.02 to 1.30) | 0.027 |
| Quintile 6 (not known) | 0.88 (0.50 to 1.53) | 0.642 |
| Alcohol misuse | 1.14 (0.92 to 1.42) | 0.221 |
| Arrhythmias | 1.27 (1.17 to 1.38) | <0.001 |
| COPD | 1.32 (1.17 to 1.50) | <0.001 |
| Cancer with metastasis | 1.10 (1.00 to 1.20) | 0.039 |
| Deficiency anaemia | 1.44 (1.18 to 1.74) | 0.003 |
| Dementia | 1.43 (1.26 to 1.63) | <0.001 |
| Depression | 1.10 (0.96 to 1.27) | 0.187 |
| Diabetes mellitus | 1.19 (1.09 to 1.31) | 0.0002 |
| Fluid disorders | 2.29 (2.04 to 2.56) | <0.001 |
| Heart failure | 1.03 (0.96 to 1.10) | 0.493 |
| Hypertension | 1.00 (0.89 to 1.12) | 0.949 |
| Hypothyroidism | 2.08 (1.54 to 2.81) | <0.001 |
| Liver disease | 0.83 (0.60 to 1.15) | 0.270 |
| Obesity | 1.25 (1.05 to 1.48) | 0.012 |
| Other neurological conditions | 1.47 (1.28 to 1.69) | <0.001 |
| Peripheral vascular disease | 2.12 (1.53 to 2.92) | <0.001 |
| Paraplegia | 1.73 (1.29 to 2.32) | 0.0003 |
| Pulmonary circulatory disorders | 1.05 (0.86 to 1.28) | 0.652 |
| Renal disease | 1.20 (1.08 to 1.33) | 0.0004 |
| Rheumatological disorders | 1.17 (1.04 to 1.32) | 0.012 |
| Solid tumours | 1.68 (1.39 to 2.04) | <0.001 |
| Valvular disease | 1.30 (1.13 to 1.49) | 0.0002 |

<u>Final destination on discharge</u>

89.3% of identified patients were admitted from their usual residence and 74.9% went back there afterwards: 8.9% went to a care home; 5.3% were discharged to a temporary residence; 4.3% died in hospital and 3.6% went to another hospital but for which no HES record could be linked.



For peer review only - http://bmjopen.bmj.com/site/about/guidelines.xhtml

The total 30-day mortality rate was 3.3% for the period April 1 2015 to June 30 2018 (deaths via linkage to the national deaths registry were available up to dates including July 31 2018).

Figure 4 shows Kaplan-Meier survival curves for non-operative versus operative cases. See Supplementary Figures 1 & 2 for Kaplan-Meier survival curves by sex and age range respectively.

<Figure 4 here>

**Discussion**

This study sought to establish the burden of periprosthetic fractures on the healthcare system in the UK and identify any factors associated with differences in treatment or outcome. The number of patients admitted to hospital in England with periprosthetic fractures is currently around 450 per month. The overall number of periprosthetic fractures as coded by M96.6 is increasing by approximately 13% each year. Periprosthetic fractures are associated with a relatively prolonged length of stay and increased dependency upon discharge with 25% unable to return directly to their normal residence. 73% of people admitted with a periprosthetic fracture received an operation. Non-operative management was more common in those with co-morbidities, increasing age, and COPD. Mortality was low, at 4.3% for in-hospital and 3.3% for total 30-day mortality; for proximal femoral fractures, 30-day mortality from the latest NHFD report is higher, at 6.1%[3].

There are limitations to the data, and we have tried not to over-interpret the data we have. We cannot be certain how complete and accurate the M96.6 coding is. The study relies on accurate data recorded by clinicians and then coded by clinical coders in hospitals. The primary diagnosis and procedure fields in administrative data are known to have high accuracy (>95%)[10], though secondary diagnoses are subject to some under-recording. There will be variation between hospitals as to when the coding advice was implemented, which is why we estimated the trend in two ways. We have attempted to correct for this by analysing ‘early adopters’ separately from the complete cohort. Reassuringly, the conclusions are broadly the same in terms of absolute numbers and relative rate of rise. We did not attempt to distinguish between the sites of periprosthetic fractures, and some of these fractures will have been following arthroplasty of other joints, and fracture fixation, particularly neck of femur fracture.[11] M96.6 is not associated with an anatomical code to specify the bone and or joint involved. It is possible to attempt to infer the joint involved by any subsequent operation codes (M96.6 itself has no anatomical modifier), but this method is not guaranteed to be accurate and does not provide any information regarding the site of injury of those who were treated non-operatively (approximately 27% of all cases). Nonetheless, despite these limitations, we are confident that the increasing trend amongst older people with joint implants accurately reflects the everyday clinical situation.

HES data only provide in-hospital mortality, but we were able to use the established linkage to the national death registry for deaths up to the end of July 2018. More importantly, there are no direct data on functional outcomes.[12] Such additional data would be invaluable to allow clinicians and patients to discuss the most appropriate management.

This is the first study utilising the specific clinical code M96.6. This was introduced in March 2015, and although Figure 1 shows a dramatic increase when the coding advice was introduced, we are confident that there is a significant increasing underlying trend in patients



For peer review only - http://bmjopen.bmj.com/site/about/guidelines.xhtml

with periprosthetic fractures. There have been few studies that have examined the periprosthetic patient population from a multidisciplinary perspective. There are no studies examining risk assessment for these patients with the exception of a small retrospective analysis, 39 patients with distal femoral fractures from Cleveland Ohio.[13]

Our data are in line with previous studies of complication rates which have demonstrated periprosthetic fracture rates of 0.1-18% for hip, 0.3-5.5% for knee, and 0.5-3% for shoulder arthroplasties.[14]

The number of peri-prosthetic fractures is likely to increase, given the ageing population, increased life expectancy and increasing numbers of people with joint replacements. How these individuals are managed is one of the issues that needs to be addressed in order to provide optimum care. The likely treatment of these injuries varies depending on the location involved and the stability of the implant.

The extensive surgery involved in the operative treatment options for these individuals mean that these patients have to be able to withstand a more significant surgical insult than individuals undergoing primary joint arthroplasty or those being treated for neck of femur fractures. There are also implications for the use of hospital resources with longer operation times, expensive implants, and the availability of revision arthroplasty specialists in addition to the higher cost of post-operative care, including high dependency care.[15]

In-hospital mortality is significant, at around 5%, and the rate of new discharge to a care home of around 15% suggests an increase in dependency of this patient group – this representing those who whilst admitted from their usual residence were discharged to a different location.

For patients presenting with hip fracture, surgical management is almost always viewed as gold standard care for pain relief and to restore mobility where possible. Early surgery is important to allow the patient to sit up thereby reducing risk of respiratory problems, malnutrition and difficulty in toileting. This may be different in peri-prosthetic fractures, particularly those around the knee as patients may be able to tolerate sitting up and pain may be controlled by immobilising the fracture in a cast or brace.

Some patients with frailty do not have the physiological reserve to survive major surgery and this will influence decisions between non-operative management, fixation, and major revision surgery.

For all patients with frailty, either cognitive or physical, the decision regarding surgery should be made with the individual and the multidisciplinary team and in some cases may be viewed as a palliative procedure to manage pain.

Notwithstanding the limitations of our analysis, periprosthetic fractures are likely to create an increasing burden to patients, clinicians and the wider health and social care services. At current rates, the average UK trust will admit around one patient with a periprosthetic fracture each week. If the current trend of a 13% annual increase continues, this will rise to around 2 patients per week in 5- or 6-years' time, equating to around 700 extra bed-days per trust per year, alongside an increase in required operating theatre capacity. These patients are often complex, both surgically and medically, and hospitals may wish to consider whether a specialist local or regional service is provided to ensure optimal decision making and


For peer review only - http://bmjopen.bmj.com/site/about/guidelines.xhtml

management. The costs associated with treating periprosthetic fractures often outstrips reimbursement from funders and so any such service will need to be designed in conjunction with funders to ensure it is financially viable.[16]

Data collection and accurate reporting are going to be needed to identify all of the cases, and an add-on to the NJR is required for complete data capture. If a comparison with proximal femoral fracture is made, the NHS is probably where it was with hip fracture care around 15 years ago, when the care of periprosthetic fractures is concerned. The great improvement in hip fracture care ran in parallel with the emergence of "orthogeriatrics" as a definite entity. Periprosthetic fractures will need the same level of multidisciplinary input that is adequately funded and using all the experience gained with the hip fracture population.

Will individual units be able to cope with demand or will there be regional specialisation? The resources needed will not be limited to orthopaedic surgery, where there will need to be enough adequately trained arthroplasty revision surgeons, but will include perioperative medicine, orthogeriatric input, high dependency care, nursing, physiotherapy and care home provision. Perhaps, dedicated units that deal with hip fractures and periprosthetic fractures will be the model of care for the future, with expertise and training opportunities concentrated in purpose-built units.

The coding change made in 2015 to make the NHS ICD-10 compliant has made the identification of periprosthetic fractures easier. There already appears to be an increase of 15% per year in these cases. This requires planning for future service provision at local and national level. This may be facilitated by prospective data collection using a system similar to the National Joint Registry or National Hip Fracture Database.

**Data Sharing**

Due to information governance rules applicable to HES, no data are available for sharing. HES data is available directly from NHS Digital. Please see: https://digital.nhs.uk/data-and-information/data-tools-and-services/data-services/hospital-episode-statistics/users-uses-and-access-to-hospital-episode-statistics#access-to-data

**Author Contributions**

AB devised the study, extracted and analysed the data, wrote the manuscript, reviewed the manuscript.
RG devised the study, reviewed the data, wrote the manuscript, reviewed the manuscript.
SMW reviewed the data, wrote the manuscript, reviewed the manuscript.
HWJ reviewed the data, wrote the manuscript, reviewed the manuscript.
PA reviewed the data, wrote the manuscript, reviewed the manuscript.
IM reviewed the data, wrote the manuscript, reviewed the manuscript.
EAHC devised the study, reviewed the data, reviewed the manuscript.
HW reviewed the data, wrote the manuscript, reviewed the manuscript.
BMD devised the study, reviewed the data, wrote the manuscript, reviewed the manuscript, and guarantees the paper.

**Acknowledgements**

We would like to thank Ketan Patel for his assistance regarding the use of the code M96.6.

For peer review only

**Funding**
AB and PA work at the Dr Foster Unit, an academic unit in the Department of Primary Care and Public Health, Imperial College London. The unit receives research funding from Dr Foster Intelligence, an independent health service research organization (a wholly owned subsidiary of Telstra). The Dr Foster Unit at Imperial is affiliated with the National Institute of Health Research (NIHR) Imperial Patient Safety Translational Research Centre. The NIHR Imperial Patient Safety Translational Centre is a partnership between the Imperial College Healthcare NHS Trust and Imperial College London. The Department of Primary Care and Public Health at Imperial College London is grateful for support from the NW London NIHR Collaboration for Leadership in Applied Health Research & Care (CLAHRC) and the Imperial NIHR Biomedical Research Centre.

BMD is funded by an NIHR Clinical Lecturer position (CL-2016-14-009).

Funders did not have any input to study conception, design, analysis or manuscript writing.

**Competing Interest Statement**
AB reports grants from Dr Foster, grants from Medtronic, outside the submitted work
RG has nothing to disclose.
HWJ has received payment in past for educational and consultation services to DePuy Synthes and Stryker. Institutional grants received from DePuy Synthes for International Surgeon visitations. Trustee of The John Charnley Trust.
EAHC has nothing to disclose.
PA has nothing to disclose.
BMD is funded by a National Institute of Health Research Clinical Lectureship appointment.
HW has nothing to disclose.
IM has nothing to disclose.
SW has nothing to disclose.

**Disclaimer**
This publication presents independent research funded by the National Institute for Health Research (NIHR). The views expressed are those of the author(s) and not necessarily those of the NHS, the NIHR or the Department of Health and Social Care.

**References**
1. Learmonth I D, Young C, Rorabeck C. The operation of the century; total hip replacement. Lancet 2007;**307**:1508–19.
2. National Joint Registry 16th Report. Available from URL: https://reports.njrcentre.org.uk/Portals/0/PDFdownloads/NJR%2016th%20Annual%20Report%202019.pdf (accessed 20/12/2019)
3. National Hip Fracture Database Annual Report 2019. Available from URL: https://www.nhfd.co.uk/files/2019ReportFiles/NHFD_2019_Annual_Report.pdf (accessed 20/12/2019)
4. Office for National Statistics 2015. Available from URL: https://www.ons.gov.uk/peoplepopulationandcommunity/birthsdeathsandmarriages/lifeexpectancies/bulletins/lifeexpectancyatbirthandatage65bylocalareasinenglandandwales/2015-11-04 (accessed 09/08/2018)
5. Clegg A, Young J, Iliffe S, Rikkert MO, Rockwood K. Frailty in elderly people. Lancet 2013;**381**:752–762.


For peer review only - http://bmjopen.bmj.com/site/about/guidelines.xhtml

6. Clinical Classifications Service – Health & Social Care Information Centre. Coding Clinic Version 4.1. March 2015.
7. NHS Digital. Hospital Episode Statistics. Available from URL: https://digital.nhs.uk/data-and-information/data-tools-and-services/data-services/hospital-episode-statistics (accessed Oct 11 2018)
8. International Classification of Diseases ICD-10. Available from URL: http://apps.who.int/classifications/icd10/browse/2010/en (accessed 10/08/2018)
9. Carstairs V, Morris R. Deprivation and Health in Scotland. Aberdeen University Press, 1991.
10. Burns EM, Rigby E, Mamidanna R, Bottle A, Aylin P, Ziprin P, Faiz OD. Systematic review of discharge coding accuracy. J Public Health (Oxf) 2012;**34(1)**:138–48.
11. Leal J, Gray AM, Prieto-Alhambra D, Arden NK, Cooper C, Jarvaid MK, Judge A. Impact of hip fracture on hospital care costs: a population-based study. Osteoporosis Int 2016;**27**:549–558.
12. Moppett I. Hip fractures: are we asking the right questions? Age Ageing 2018;**47(5)**:633–4.
13. Reich MS, Duong M, Breslin MA, Nguyen MP, Vallier HA. Perioperative Considerations when treating isolated periprosthetic distal femur fractures. The Iowa Orthopedic Journal 2017;**37**:41–45.
14. Capone A, Congia S, Civinini R, Marongiu G. Periprosthetic fractures: epidemiology and current treatment. Clin Cases Miner Bone Metab. 2017;**14(2)**:189–96.
15. Griffiths EJ, Cash DJW, Kalra S, Hopgood PJ. Time to surgery and 30-day morbidity and mortality of periprosthetic hip fractures. Injury. 2013 Dec 1;44(12):1949–52.
16. Jones AR, Williams T, Paringe V, White SP. The economic impact of surgically treated peri-prosthetic hip fractures on a university teaching hospital in Wales 7.5-year study. Injury 2016;47(2):428–31.


I, the Submitting Author has the right to grant and does grant on behalf of all authors of the Work (as defined in the below author licence), an exclusive licence and/or a non-exclusive licence for contributions from authors who are: i) UK Crown employees; ii) where BMJ has agreed a CC-BY licence shall apply, and/or iii) in accordance with the terms applicable for US Federal Government officers or employees acting as part of their official duties; on a worldwide, perpetual, irrevocable, royalty-free basis to BMJ Publishing Group Ltd ("BMJ") its licensees and where the relevant Journal is co-owned by BMJ to the co-owners of the Journal, to publish the Work in BMJ Open and any other BMJ products and to exploit all rights, as set out in our licence.

The Submitting Author accepts and understands that any supply made under these terms is made by BMJ to the Submitting Author unless you are acting as an employee on behalf of your employer or a postgraduate student of an affiliated institution which is paying any applicable article publishing charge ("APC") for Open Access articles. Where the Submitting Author wishes to make the Work available on an Open Access basis (and intends to pay the relevant APC), the terms of reuse of such Open Access shall be governed by a Creative Commons licence – details of these licences and which Creative Commons licence will apply to this Work are set out in our licence referred to above.



For peer review only - http://bmjopen.bmj.com/site/about/guidelines.xhtml

**Figure legends**

Figure 1. Number of admissions by month from April 2013 to December 2018, with elective and emergency admissions shown separately

Figure 2. Acute hospital length of stay by age and gender

Figure 3. Acute hospital length of stay by operative management

Figure 4. Kaplan Meier Survival Curve by Operative Status

For peer review only

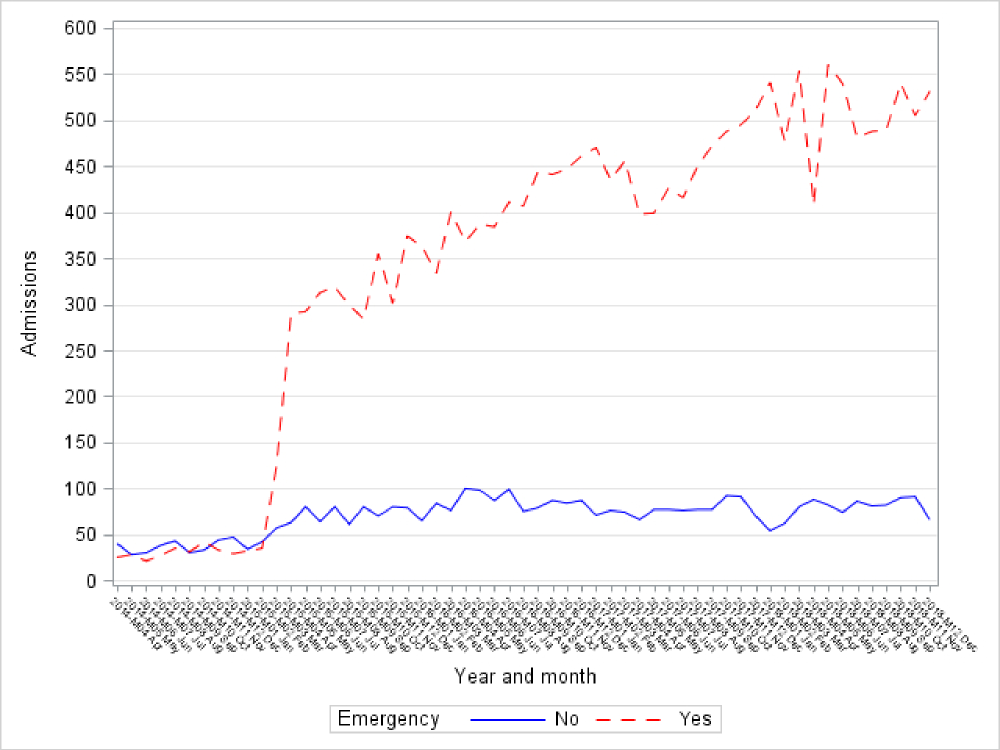


Figure 1. Number of admissions by month from April 2013 to December 2018, with elective and emergency admissions shown separately

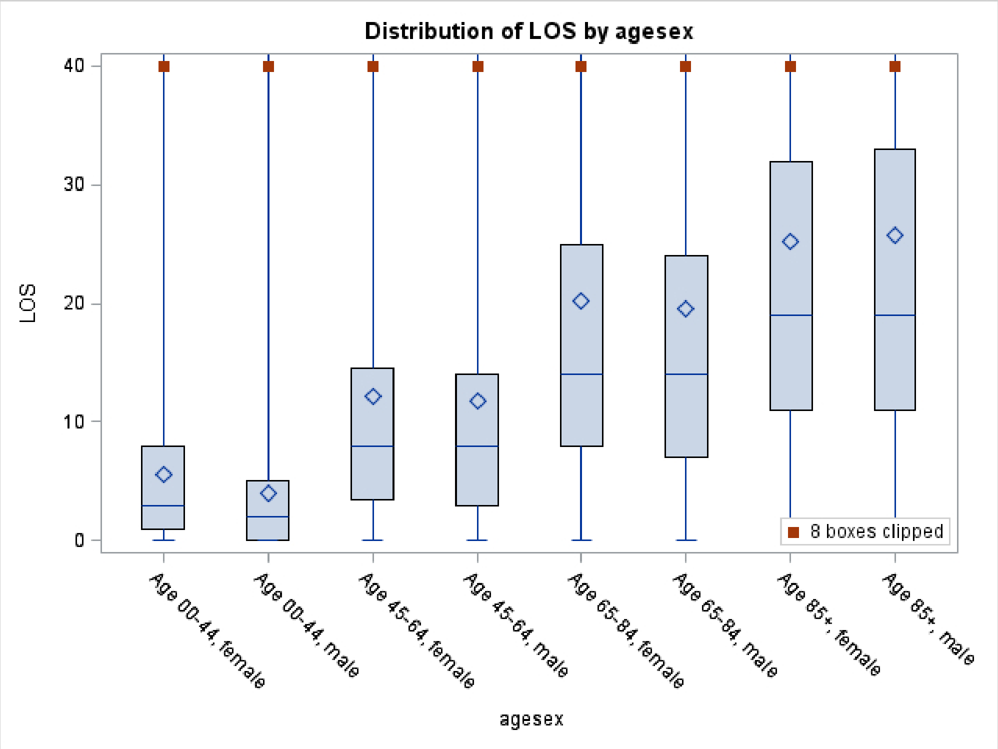


Figure 2. Acute hospital length of stay by age and gender

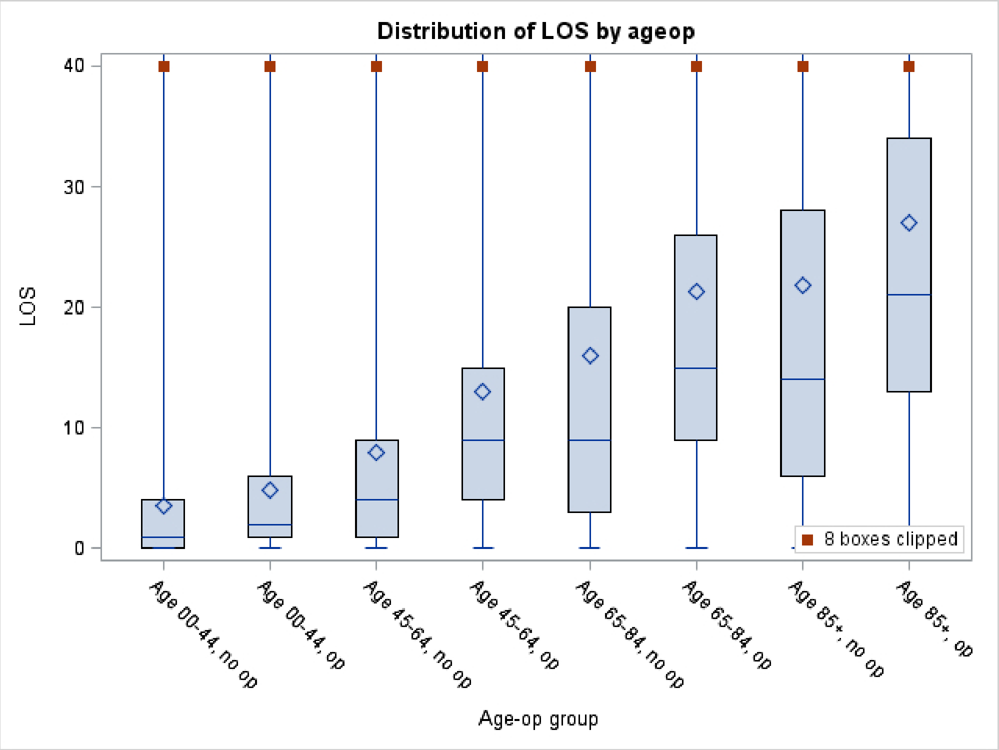


Figure 3. Acute hospital length of stay by operative management

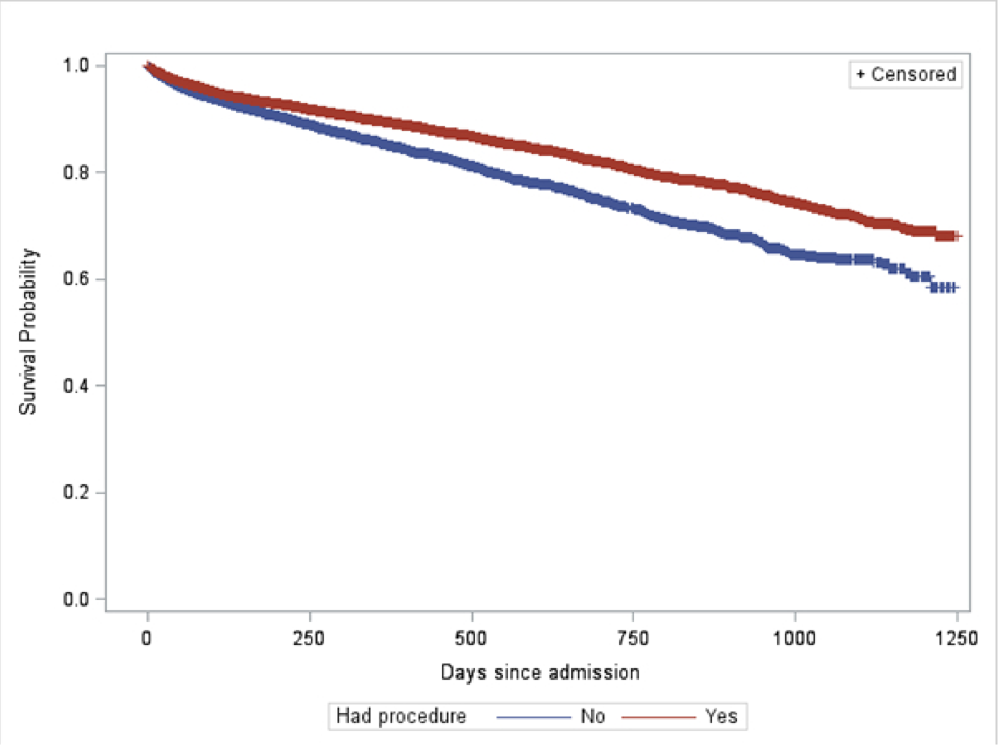


Figure 4. Kaplan Meier Survival Curve by Operative Status

# Supplementary Data - Periprosthetic fractures – the next fragility fracture epidemic? A national observational study

**Supplementary Table 1. The 20 most commonly performed procedures by four-character OPCS code.**

| Op code | Description | Number | Proportion of operations (%) |
|---|---|---|---|
| W201 | Primary open reduction of fracture of long bone and extramedullary fixation using plate NEC | 5004 | 14.8 |
| W283 | Removal of internal fixation from bone NEC | 1728 | 5.1 |
| W202 | Primary open reduction of fracture of long bone and extramedullary fixation using cerclage | 1122 | 3.3 |
| W373 | Revision of total prosthetic replacement of hip joint using cement | 843 | 2.5 |
| W383 | Revision of total prosthetic replacement of hip joint not using cement | 633 | 1.9 |
| W384 | Revision of one component of total prosthetic replacement of hip joint not using cement | 617 | 1.8 |
| W192 | Primary open reduction of fracture of long bone and fixation using rigid nail NEC | 591 | 1.7 |
| W382 | Conversion to total prosthetic replacement of hip joint not using cement | 588 | 1.7 |
| W370 | Conversion from previous cemented total prosthetic replacement of hip joint | 585 | 1.7 |
| W403 | Revision of total prosthetic replacement of knee joint using cement | 582 | 1.7 |
| W374 | Revision of one component of total prosthetic replacement of hip joint using cement | 501 | 1.5 |
| W901 | Aspiration of joint | 495 | 1.5 |
| W281 | Application of internal fixation to bone NEC | 448 | 1.3 |
| W232 | Secondary open reduction of fracture of bone and extramedullary fixation HFQ | 420 | 1.2 |
| W322 | Allograft of bone NEC | 389 | 1.1 |
| W246 | Closed reduction of fracture of bone and fixation using nail or screw | 333 | 1.0 |
| W191 | Primary open reduction of fracture of neck of femur and open fixation using pin and plate | 310 | 0.9 |
| W242 | Closed reduction of fracture of long bone and rigid internal fixation NEC | 300 | 0.9 |
| W198 | Other specified primary open reduction of fracture of bone and intramedullary fixation | 298 | 0.9 |
| W381 | Primary total prosthetic replacement of hip joint not using cement | 258 | 0.8 |
| other | All other main procedures combined | 33922 | 53.7 |

NEC = not elsewhere classified

For peer review only

**Supplementary Table 2. Operative rate according to age, gender and method of admission**

| Factor | Elective admissions with operation | Emergency admissions with operation | % of all elective admissions with operation | % of all emergency admissions with operation |
|---|---|---|---|---|
| Age 0-44, female | 65 | 98 | 95.6 | 66.7 |
| Age 0-44, male | 146 | 225 | 88.5 | 74.3 |
| Age 45-64, female | 229 | 636 | 95.0 | 76.9 |
| Age 45-64, male | 164 | 476 | 92.1 | 77.5 |
| Age 65-84, female | 552 | 4058 | 82.5 | 73.6 |
| Age 65-84, male | 304 | 2298 | 85.4 | 74.9 |
| Age 85+, female | 140 | 3249 | 64.5 | 67.0 |
| Age 85+, male | 56 | 1092 | 62.2 | 69.0 |
| Total | 1656 | 12132 | 83.5 | 71.8 |

**Supplementary Table 3. Length of stay in nights according to major operation status**

| Patient group | LOS type | N | Lower quartile | Median | Mean | Upper quartile |
|---|---|---|---|---|---|---|
| All | Acute stay | 18888 | 7 | 14 | 20.5 | 26 |
| All | Total | 18888 | 8 | 17 | 25.0 | 33 |
| No major operation | Acute stay | 5100 | 4 | 10 | 17.6 | 22 |
| No major operation | Total | 5100 | 4 | 11 | 19.8 | 26 |
| Major operation | Acute stay | 13788 | 9 | 16 | 21.6 | 27 |
| Major operation | Total | 13788 | 10 | 19 | 26.9 | 35 |

"Acute stay" means the stay at the first (acute) hospital; "Total" covers the whole admission, including inter-hospital transfers

**Supplementary Table 4. Length of stay in nights by age and gender**

| Patient group | LOS type | N | Lower quartile | Median | Mean | Upper quartile |
|---|---|---|---|---|---|---|
| Age 0-44, female | Acute stay | 215 | 1 | 3 | 5.6 | 8 |
| | Total | 215 | 1 | 3 | 6.8 | 9 |
| Age 0-44, male | Acute stay | 468 | 0 | 2 | 4.0 | 5 |
| | Total | 468 | 0 | 2 | 4.7 | 5 |
| Age 45-64, female | Acute stay | 1068 | 3.5 | 8 | 12.1 | 14.5 |
| | Total | 1068 | 4 | 9 | 14.6 | 17 |
| Age 45-64, male | Acute stay | 792 | 3 | 8 | 11.8 | 14 |
| | Total | 792 | 3 | 8 | 13.5 | 15 |
| Age 65-84, female | Acute stay | 6186 | 8 | 14 | 20.2 | 25 |
| | Total | 6186 | 9 | 17 | 24.6 | 31 |
| Age 65-84, male | Acute stay | 3424 | 7 | 14 | 19.5 | 24 |
| | Total | 3424 | 8 | 16 | 23.9 | 30 |
| Age 85+, female | Acute stay | 5063 | 11 | 19 | 25.2 | 32 |
| | Total | 5063 | 12 | 23 | 31.0 | 41 |
| Age 85+, male | Acute stay | 1672 | 11 | 19 | 25.7 | 33 |
| | Total | 1672 | 13 | 23 | 31.4 | 43 |

"Acute stay" means the stay at the first (acute) hospital; "Total" covers the whole admission, including inter-hospital transfers



For peer review only - http://bmjopen.bmj.com/site/about/guidelines.xhtml

For peer review only

**Supplementary Table 5. Length of stay in nights by age, gender and operative status**

| Patient group | LOS type | Operated | N | Lower quartile | Median | Mean | Upper quartile |
|---|---|---|---|---|---|---|---|
| Age 0-44, female | Acute stay | No | 52 | 1 | 2 | 4.0 | 5 |
| | | Yes | 52 | 1 | 3 | 6.1 | 8 |
| | Total | No | 163 | 1 | 2 | 4.4 | 6 |
| | | Yes | 163 | 1 | 4 | 7.6 | 10 |
| Age 0-44, male | Acute stay | No | 97 | 0 | 1 | 3.2 | 3 |
| | | Yes | 97 | 1 | 2 | 4.3 | 5 |
| | Total | No | 371 | 0 | 1 | 3.6 | 3 |
| | | Yes | 371 | 1 | 2 | 5.0 | 6 |
| Age 45-64, female | Acute stay | No | 203 | 1 | 4 | 8.3 | 11 |
| | | Yes | 203 | 4 | 9 | 13.0 | 15 |
| | Total | No | 865 | 1 | 4 | 10.2 | 13 |
| | | Yes | 865 | 5 | 10 | 15.6 | 18 |
| Age 45-64, male | Acute stay | No | 152 | 1 | 3 | 7.4 | 7.5 |
| | | Yes | 152 | 4 | 9 | 12.8 | 15 |
| | Total | No | 640 | 1 | 4 | 8.9 | 8 |
| | | Yes | 640 | 4 | 10 | 14.5 | 17 |
| Age 65-84, female | Acute stay | No | 1576 | 3 | 10 | 16.4 | 20 |
| | | Yes | 1576 | 9 | 16 | 21.4 | 26 |
| | Total | No | 4610 | 3 | 10 | 17.9 | 23 |
| | | Yes | 4610 | 11 | 19 | 26.8 | 33 |
| Age 65-84, male | Acute stay | No | 822 | 2 | 8 | 15.1 | 19 |
| | | Yes | 822 | 9 | 15 | 20.9 | 25 |
| | Total | No | 2602 | 2 | 8 | 16.5 | 21 |
| | | Yes | 2602 | 10 | 18 | 26.1 | 32 |
| Age 85+, female | Acute stay | No | 1674 | 6 | 14 | 21.8 | 28 |
| | | Yes | 1674 | 13 | 20 | 26.9 | 34 |
| | Total | No | 3389 | 7 | 16 | 25.3 | 33 |
| | | Yes | 3389 | 15 | 26 | 33.7 | 44 |
| Age 85+, male | Acute stay | No | 524 | 7 | 14 | 21.9 | 27 |
| | | Yes | 524 | 13 | 21 | 27.5 | 34 |
| | Total | No | 1148 | 7 | 16 | 24.9 | 34 |
| | | Yes | 1148 | 16 | 26 | 34.3 | 46 |

"Acute stay" means the stay at the first (acute) hospital; "Total" covers the whole admission, including inter-hospital transfers

For peer review only

**Supplementary Figure 1. Kaplan Meier Survival Curve by sex**

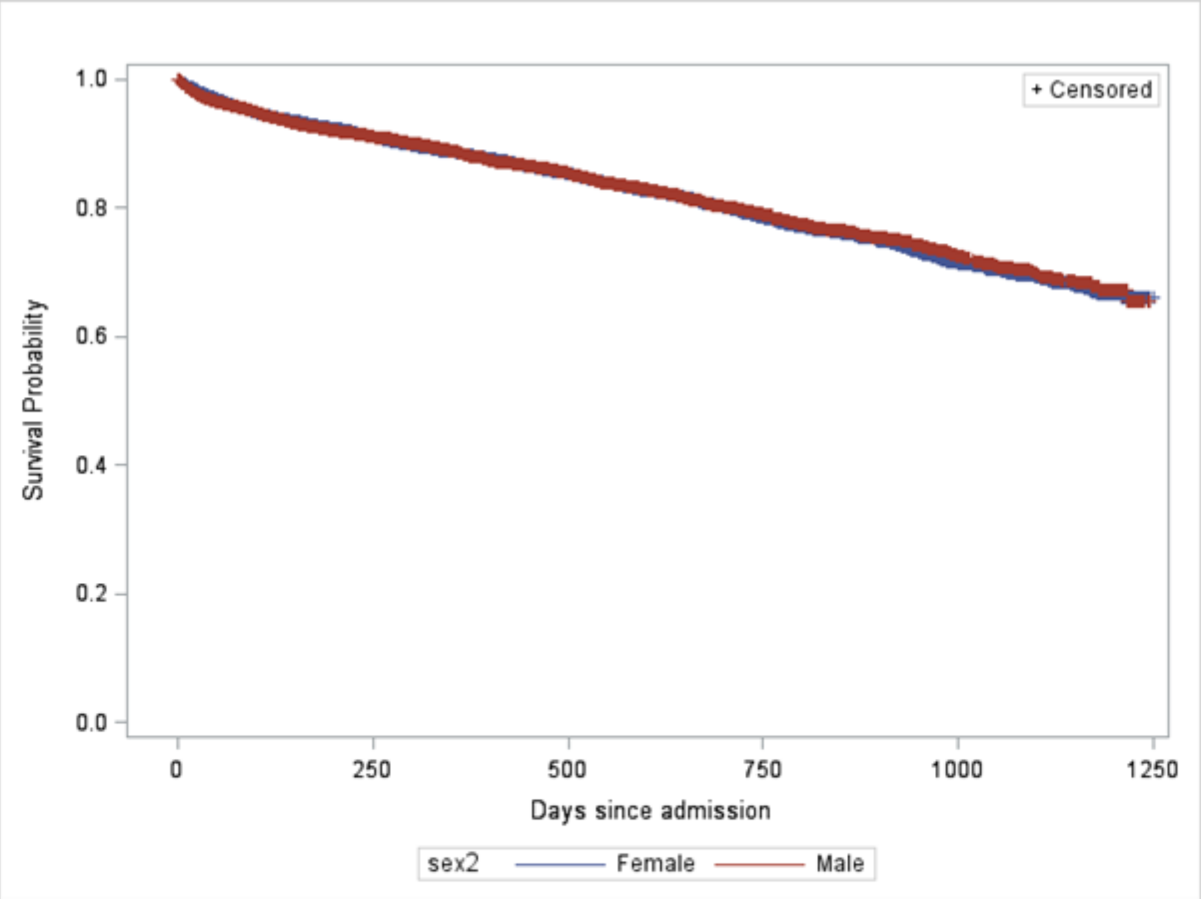


For peer review only

**Supplementary Figure 2. Kaplan Meier Survival Curve by age range**

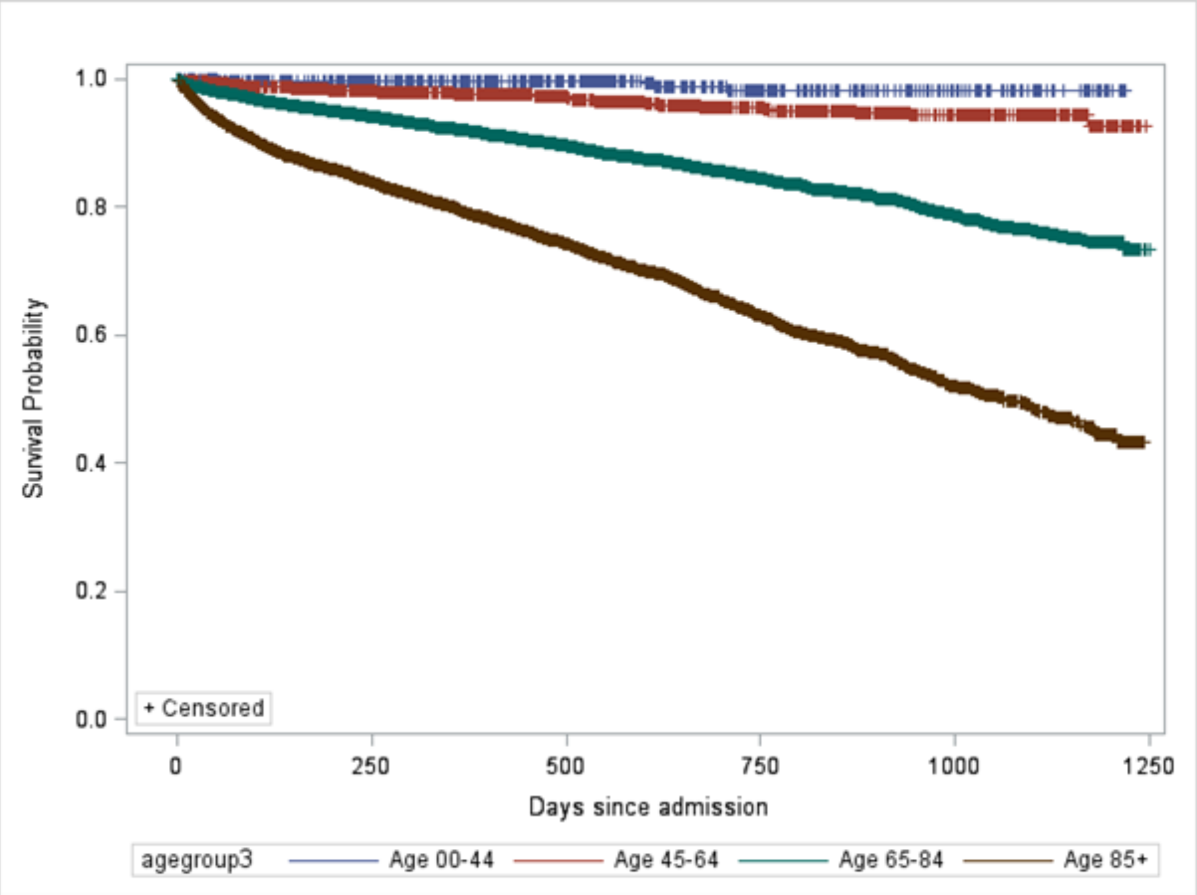




For peer review only - http://bmjopen.bmj.com/site/about/guidelines.xhtml

STROBE Statement—checklist of items that should be included in reports of observational studies

| | Item No | Recommendation | Page No |
|---|---|---|---|
| **Title and abstract** | 1 | (*a*) Indicate the study's design with a commonly used term in the title or the abstract | 1 |
| | | (*b*) Provide in the abstract an informative and balanced summary of what was done and what was found | 2 |
| **Introduction** | | | |
| Background/rationale | 2 | Explain the scientific background and rationale for the investigation being reported | 3 |
| Objectives | 3 | State specific objectives, including any prespecified hypotheses | 3 |
| **Methods** | | | |
| Study design | 4 | Present key elements of study design early in the paper | 3 |
| Setting | 5 | Describe the setting, locations, and relevant dates, including periods of recruitment, exposure, follow-up, and data collection | 3-4 |
| Participants | 6 | (*a*) *Cohort study*—Give the eligibility criteria, and the sources and methods of selection of participants. Describe methods of follow-up<br>*Case-control study*—Give the eligibility criteria, and the sources and methods of case ascertainment and control selection. Give the rationale for the choice of cases and controls<br>*Cross-sectional study*—Give the eligibility criteria, and the sources and methods of selection of participants | 3-4 |
| | | (*b*) *Cohort study*—For matched studies, give matching criteria and number of exposed and unexposed<br>*Case-control study*—For matched studies, give matching criteria and the number of controls per case | n/a |
| Variables | 7 | Clearly define all outcomes, exposures, predictors, potential confounders, and effect modifiers. Give diagnostic criteria, if applicable | 4 |
| Data sources/ measurement | 8* | For each variable of interest, give sources of data and details of methods of assessment (measurement). Describe comparability of assessment methods if there is more than one group | 3-4 |
| Bias | 9 | Describe any efforts to address potential sources of bias | |
| Study size | 10 | Explain how the study size was arrived at | 4 |
| Quantitative variables | 11 | Explain how quantitative variables were handled in the analyses. If applicable, describe which groupings were chosen and why | |
| Statistical methods | 12 | (*a*) Describe all statistical methods, including those used to control for confounding | 4 |
| | | (*b*) Describe any methods used to examine subgroups and interactions | 4 |
| | | (*c*) Explain how missing data were addressed | n/a |
| | | (*d*) *Cohort study*—If applicable, explain how loss to follow-up was addressed<br>*Case-control study*—If applicable, explain how matching of cases and controls was addressed<br>*Cross-sectional study*—If applicable, describe analytical methods taking account of sampling strategy | |
| | | (*e*) Describe any sensitivity analyses | 4 |

Continued on next page



For peer review only - http://bmjopen.bmj.com/site/about/guidelines.xhtml

| | | | |
|---|---|---|---|
| **Results** | | | |
| Participants | 13* | (a) Report numbers of individuals at each stage of study—eg numbers potentially eligible, examined for eligibility, confirmed eligible, included in the study, completing follow-up, and analysed | 4 |
| | | (b) Give reasons for non-participation at each stage | n/a |
| | | (c) Consider use of a flow diagram | |
| Descriptive data | 14* | (a) Give characteristics of study participants (eg demographic, clinical, social) and information on exposures and potential confounders | 5 (and table 3) |
| | | (b) Indicate number of participants with missing data for each variable of interest | |
| | | (c) *Cohort study*—Summarise follow-up time (eg, average and total amount) | |
| Outcome data | 15* | *Cohort study*—Report numbers of outcome events or summary measures over time | 5-6 |
| | | *Case-control study*—Report numbers in each exposure category, or summary measures of exposure | |
| | | *Cross-sectional study*—Report numbers of outcome events or summary measures | |
| Main results | 16 | (*a*) Give unadjusted estimates and, if applicable, confounder-adjusted estimates and their precision (eg, 95% confidence interval). Make clear which confounders were adjusted for and why they were included | |
| | | (*b*) Report category boundaries when continuous variables were categorized | Table 4 |
| | | (*c*) If relevant, consider translating estimates of relative risk into absolute risk for a meaningful time period | |
| Other analyses | 17 | Report other analyses done—eg analyses of subgroups and interactions, and sensitivity analyses | 6-8 |
| **Discussion** | | | |
| Key results | 18 | Summarise key results with reference to study objectives | 9-10 |
| Limitations | 19 | Discuss limitations of the study, taking into account sources of potential bias or imprecision. Discuss both direction and magnitude of any potential bias | 11 |
| Interpretation | 20 | Give a cautious overall interpretation of results considering objectives, limitations, multiplicity of analyses, results from similar studies, and other relevant evidence | 11 |
| Generalisability | 21 | Discuss the generalisability (external validity) of the study results | 11-12 |
| **Other information** | | | |
| Funding | 22 | Give the source of funding and the role of the funders for the present study and, if applicable, for the original study on which the present article is based | 12 |

*Give information separately for cases and controls in case-control studies and, if applicable, for exposed and unexposed groups in cohort and cross-sectional studies.

**Note:** An Explanation and Elaboration article discusses each checklist item and gives methodological background and published examples of transparent reporting. The STROBE checklist is best used in conjunction with this article (freely available on the Web sites of PLoS Medicine at http://www.plosmedicine.org/, Annals of Internal Medicine at http://www.annals.org/, and Epidemiology at http://www.epidem.com/). Information on the STROBE Initiative is available at www.strobe-statement.org.

For peer review only


For peer review only - http://bmjopen.bmj.com/site/about/guidelines.xhtml

BMJ Open

# Periprosthetic fractures – the next fragility fracture epidemic? A national observational study

| | |
|---|---|
| Journal: | *BMJ Open* |
| Manuscript ID | bmjopen-2020-042371.R2 |
| Article Type: | Original research |
| Date Submitted by the Author: | 05-Nov-2020 |
| Complete List of Authors: | Bottle, Alex; Imperial College London, Primary Care and Public Health<br>Griffiths, Richard; Peterborough City Hospital, Anaesthesia<br>White, Stuart; Royal Sussex County Hospital,<br>Wynn-Jones, Henry; Wrightington Hospital, The Centre for Hip Surgery<br>Aylin, Paul; Imperial College London, Primary Care and Public Health<br>Moppett, Iain; University of Nottingham, Anaesthesia & Critical Care<br>Chowdhury, Emyr; North West Anglia NHS Foundation Trust<br>Wilson, Helen; Royal Surrey County Hospital NHS Foundation Trust, Department of Geriatric Medicine<br>Davies, Benjamin; University of Cambridge, Department of Surgery |
| <b>Primary Subject Heading</b>: | Surgery |
| Secondary Subject Heading: | Epidemiology, Geriatric medicine |
| Keywords: | Adult orthopaedics < ORTHOPAEDIC & TRAUMA SURGERY, EPIDEMIOLOGY, GERIATRIC MEDICINE |
| | |

SCHOLARONE™
Manuscripts

*I, the Submitting Author has the right to grant and does grant on behalf of all authors of the Work (as defined in the below author licence), an exclusive licence and/or a non-exclusive licence for contributions from authors who are: i) UK Crown employees; ii) where BMJ has agreed a CC-BY licence shall apply, and/or iii) in accordance with the terms applicable for US Federal Government officers or employees acting as part of their official duties; on a worldwide, perpetual, irrevocable, royalty-free basis to BMJ Publishing Group Ltd ("BMJ") its licensees and where the relevant Journal is co-owned by BMJ to the co-owners of the Journal, to publish the Work in this journal and any other BMJ products and to exploit all rights, as set out in our licence.*

*The Submitting Author accepts and understands that any supply made under these terms is made by BMJ to the Submitting Author unless you are acting as an employee on behalf of your employer or a postgraduate student of an affiliated institution which is paying any applicable article publishing charge ("APC") for Open Access articles. Where the Submitting Author wishes to make the Work available on an Open Access basis (and intends to pay the relevant APC), the terms of reuse of such Open Access shall be governed by a Creative Commons licence – details of these licences and which Creative Commons licence will apply to this Work are set out in our licence referred to above.*

*Other than as permitted in any relevant BMJ Author's Self Archiving Policies, I confirm this Work has not been accepted for publication elsewhere, is not being considered for publication elsewhere and does not duplicate material already published. I confirm all authors consent to publication of this Work and authorise the granting of this licence.*

For peer review only

Title:
# Periprosthetic fractures – the next fragility fracture epidemic? A national observational study

Authors:
Alex Bottle[1] (robert.bottle@imperial.ac.uk)
Richard Griffiths[2] (richardgriffiths1@nhs.net)
Stuart M White[3] (igasbest@hotmail.com)
Henry Wynn-Jones[4] (henrywynnjones@gmail.com)
Paul Aylin[1] (p.aylin@imperial.ac.uk)
Iain Moppett[5] (iain.moppett@nottingham.ac.uk)
Emyr A H Chowdhury[6] (emyr.chowdhury@nhs.net)
Helen Wilson[7] (hwilson6@nhs.net)
Benjamin M Davies[8] (bmd35@cam.ac.uk)

Affiliations:
1 – Dr Foster Unit, Dept of Primary Care and Public Health, School of Public Health, Imperial College London, Reynolds Building, St Dunstan's Road, London, United Kingdom
2 – Department of Anaesthesia, Peterborough City Hospital, Peterborough, United Kingdom
3 – Haywards Heath Hip Fracture Unit, Princess Royal Hospital, Brighton and Sussex University Hospitals NHS Trust, Haywards Heath, United Kingdom
4 – The Centre for Hip Surgery, Wrightington Hospital, Hall Lane, Appley Bridge, Lancashire, United Kingdom
5 – Anaesthesia and Critical Care, Division of Clinical Neuroscience, The University of Nottingham, Queen's Medical Centre, Nottingham, United Kingdom
6 – Department of Trauma and Orthopaedics, Peterborough City Hospital, Peterborough, United Kingdom
7 – Department of General Medicine, Stroke and Care of the Elderly, Royal Surrey County Hospital, Guildford, United Kingdom
8 – Division of Trauma and Orthopaedics, University of Cambridge, Addenbrooke's Hospital, Cambridge, United Kingdom

Corresponding Author:
Prof Alex Bottle
Chair in Medical Statistics
Co-director, Dr Foster Unit, School of Public Health
Imperial College London
3 Dorset Rise, London EC4Y 8EN
Tel: +44 (0)207 594 0913
Email: robert.bottle@imperial.ac.uk
Keywords:
Periprosthetic fracture
Fragility fracture
Trauma
Hospital Episode Statistics

Word Count: 3,048


For peer review only - http://bmjopen.bmj.com/site/about/guidelines.xhtml

Abstract:

Objectives –

Periprosthetic fractures have considerable clinical implications for patients and financial implications for healthcare systems. This study aims to determine the burden of periprosthetic fractures of the lower and upper limbs in England and identify any factors associated with differences in treatment and outcome.

Design –

A national, observational study

Setting –

England

Participants –

All individuals admitted to hospital with peri-prosthetic factures between 01/04/2015 and 31/12/2018.

Primary and Secondary Outcome Measures –

Mortality, length of stay, change in rate of admissions.

Methods –

We analysed Hospital Episode Statistics (HES) data using the ICD-10 code M96.6 ("Fracture of bone following insertion of orthopaedic implant, joint prosthesis, or bone plate") to identify periprosthetic fractures recorded between April 2013 and December 2018. We determined the demographics, procedures performed, mortality rates, and discharge destinations. Patient characteristics associated with having a procedure during the index admission were estimated using logistic regression. The annual rate of increase in admissions was estimated using Poisson regression.

Results –

Between 01/04/2015 and 31/12/2018, there were 13,565 patients who had 18,888 admissions (89.5% emergency) with M96.6 in the primary diagnosis field. There was a 13% year-on-year increase in admissions for periprosthetic fracture in England during that period. Older people, people living in deprived areas, and those with heart failure or neurological disorders were less likely to receive an operation. 14.4% of patients did not return home after hospital discharge. The overall in-patient mortality was 4.3% and total 30-day mortality 3.3%.

Conclusions –

The clinical and operational burden of periprosthetic fractures is considerable and increasing rapidly. We suggest that the management of people with periprosthetic fractures should be undertaken and funded in a similar manner to that successfully employed for people sustaining hip fractures, utilising national standards and data collection to monitor and improve performance.

For peer review only

**Article Summary - strengths and limitations of this study**

- This is the first study to use ICD-10 code M96.6 to identify trends in peri-prosthetic fracture rates.
- This data can be used to help develop specific management pathways and allocate resources.
- Use of HES data is limited by potential inaccuracies in coding.
- The study data does not include functional outcomes which would provide useful information.

## Introduction

Since the successful introduction of total hip replacement in the 1960s, joint replacement has revolutionised the treatment of severely arthritic joints.[1] Joint replacement prostheses are available for all the major joints of the upper and lower limbs. In 2018, approximately 200,000 arthroplasty procedures were recorded in the 16th Annual report of the National Joint Registry (NJR) for England and Wales.[2] Between the advent of the registry, on 1st April 2003, and 31st December 2018, 2.75 million joint replacement procedures have been recorded. In addition, 28,684 femoral hemiarthroplasties were performed in 2018 for proximal femoral fractures.[3] The increasing life expectancy in the UK[4], combined with the effects of chronic disease and frailty[5] lead to an increased likelihood of sustaining a fracture due to osteoporosis and an increased incidence of falls. Fractures can also occur around joint replacements, and these are termed periprosthetic fractures. Periprosthetic fractures may require either fixation or revision of the implant. Surgery to treat such fractures is often complex and/or prolonged, requiring specialist input from a multidisciplinary team.

There has been no comprehensive assessment of the burden of these injuries upon the healthcare system in England. The NJR collects data on revision arthroplasty and identifies those performed for peri-prosthetic fracture. Fractures treated without the need to revise the implant are not recorded at present in the NJR. The alternative sources of data are the UK's four national hospital administrative databases: Hospital Episodes Statistics (HES) for England, and similar systems for each of the other three countries. Clinical coders use ICD-10 and UK-specific OPCS coding systems to record diagnosis and procedure information respectively. Owing to the complex classification of fractures and different techniques to treat them, there are many operative codes that could be used. In March 2015, NHS England issued new guidance to clinical coders regarding periprosthetic fractures.[6] The new ICD10 code M96.6 ("Fracture of bone following insertion of orthopaedic implant, joint prosthesis, or bone plate") is designed to capture all patients who have experienced a periprosthetic fracture at any time during an episode of care, regardless of site or operative treatment. It also captures fractures around fixation devices such as plates and intramedullary nails.

The purpose of this observational study is to quantify the burden of these cases on the NHS and identify any trends in the treatment of these conditions which may inform future healthcare resource planning. Using England's national hospital administrative database, HES, we describe the admissions with the new M96.6 diagnosis code, time trends, patient characteristics, operative rate and short-term outcomes. A literature review carried out by the authors did not identify any previous reporting of these national level data.

## Methods

<u>Data</u>

Hospital Episode Statistics (HES)[7] comprises over 125 million admitted patient, outpatient and emergency department records from hospitals within England's National Health Service (NHS) annually. Inpatient records consist of consultant episodes: each episode covers the period during which a patient is the responsibility of a given physician. A single admission at a given hospital may comprise multiple episodes. Episodes were linked into admissions and linkage with inter-hospital transfers creates "superspells"; we will use the term "admission" throughout to refer to superspells. HES provides data on in-hospital mortality and is linked to the national death register. One primary and up to 19 secondary diagnoses are coded using ICD-10;[8] one primary and up to 23 secondary procedures are coded using the UK's own "OPCS" coding system. Area-level socio-economic deprivation status in population-weighted



For peer review only - http://bmjopen.bmj.com/site/about/guidelines.xhtml

quintiles was linked to HES via the patient's postcode. Coding is completed by specialist clinical coders (rather than clinicians) in hospitals from the medical record. Once received centrally the data is cleaned to remove duplicates.

We extracted all admissions from HES with the ICD-10 code M96.6 in the primary diagnosis field (which corresponds to the main problem treated) and discharge dates between April 2013 and December 2018.

Outcome measures

We report the primary procedure performed, length of stay (acute hospital and superspell), final destination on discharge, in-hospital mortality rate and total 30-day mortality rate.

We also report the proportion of admissions with a procedure, excluding scans via the U chapter of OPCS (Diagnostic Imaging, Testing and Rehabilitation), urinary catheterisation, and auxiliary or minor procedures via the X, Y and Z chapters of OPCS (Miscellaneous and Subsidiary Classification of Methods and Sites of Operation).

Statistical analysis

With any coding change, some variation in the uptake rate between hospitals is expected. We tried to separate the uptake of the new code from any genuine underlying change in the number of admissions by dividing hospital trusts into "early adopters" and the rest. "Early adopters" were defined as those with two or more discharges with M96.6 as the primary diagnosis in March or April 2015. Simple Poisson regression models estimated the linear trend in admissions for each group of trusts ("early adopters" and "other") and for all combined. As a sensitivity analysis the modelling was repeated after excluding the first six months of the new coding period, i.e. including only admissions from October 2015 onwards.

Patient characteristics were described for the cohort from April 2015 including: age, gender, method of admission (whether emergency or not), area-level socio-economic deprivation[9] and comorbidity (using the HES ICD-10 secondary diagnosis codes). The proportion of admissions with a procedure coded, as defined above, was noted and the factors associated with a procedure derived from a multivariable logistic regression model with age, gender, deprivation and comorbidities as covariates entered at once with no selection or elimination. To determine what factors were associated with increased LOS a multivariable logistic regression was performed with the same predictors as for having a procedure; again, all predictors were retained in the model irrespective of their p-value. We performed the usual diagnostics for logistic regression, including checking for collinearity between predictors, goodness of fit and residuals. These were satisfactory. In the absence of an accepted definition of extended LOS in these cases we used the Upper Quartile boundary (26) to define a long LOS. Kaplan-Meier curves were plotted for overall survival since admission.

Ethical Approval

We have approval from the Secretary of State and the Health Research Authority under Regulation 5 of the Health Service (Control of Patient Information) Regulations 2002 to hold confidential data and analyse them for research purposes (CAG ref 15/CAG/0005). We have approval to use them for research and measuring quality of delivery of healthcare, including for this analysis, from the London - South East Ethics Committee (REC ref 15/LO/0824).


For peer review only - http://bmjopen.bmj.com/site/about/guidelines.xhtml

Patient and Public Involvement

This research was done without patient involvement. As an observational study of national level data it was not possible to involve these groups. The suggested next steps of designing services to treat these injuries appropriately would benefit from patient and public involvement.

**Results**

Between 1st April 2013 and 31st December 2018, there were 24,697 admissions with M96.6 recorded in any diagnosis field. After the coding guidance was introduced in March 2015, there were 22,955 such admissions (84.5% classified as emergency) for 16,105 patients. For the 80.3% who had M96.6 in the primary diagnosis field, there were 18,888 admissions (89.5% emergency) for 13,565 patients.

Change in admissions over time

Table 1 gives the number of admissions by year, split by whether M96.6 was in the primary or in the secondary diagnosis fields.

*Table 1: Total numbers of admissions with M96.6 recorded since April 2013*

| Financial year (Apr 1 to Mar 31) | Numbers of admissions with M96.6 as primary diagnosis | Numbers of admissions with M96.6 only as secondary diagnosis |
|---|---|---|
| 2013/4 | 429 | 384 |
| 2014/5 | 514 | 415 |
| 2015/16 | 3832 | 974 |
| 2016/17 | 5232 | 1077 |
| 2017/18 | 5435 | 1123 |
| 2018/19 to Dec 31 2018 | 4389<br>(5852 full year pro rata) | 893<br>(1191 full year pro rata) |
| Total | 19831 | 4866 |

The subsequent analysis is restricted to admissions with M96.6 in the primary diagnosis.

Figure 1 illustrates admissions by month since April 2014 to December 2018, with elective and emergency admissions shown separately. The rapid rise in admissions from April 2015 coincides with the introduction of the M96.6 coding guidance.

<Figure 1 here>

116 out of 141 hospital trusts using the M96.6 code were labelled early adopters. The rate of increase (slope) for the non-early adopters was significantly higher than that for the early adopters (p=0.001 for the interaction term). Taking the early adopters as being the “true” underlying trend, there was a 13% annual rise in M96.6 admissions between April 2015 and December 2018 (Table 2). Excluding the first six months since the coding change did not change the rate of increase significantly.



For peer review only - http://bmjopen.bmj.com/site/about/guidelines.xhtml

*Table 2: Poisson regression showing annual increase in use of M96.6 diagnosis code nationally and split by whether hospitals were early adopters of this code*

| Set of trusts | Relative Risk per month | 95% CI | Relative Risk per year | 95% CI |
|---|---|---|---|---|
| All | 1.011 | 1.010 - 1.012 | 1.14 | 1.13-1.15 |
| Early M96.6 adopters | 1.010 | 1.009 - 1.011 | 1.13 | 1.11-1.14 |
| Non early adopters | 1.016 | 1.013 - 1.018 | 1.21 | 1.17-1.24 |
| All (Oct 2015 onwards only) | 1.009 | 1.008 - 1.010 | 1.11 | 1.10-1.13 |

## Patient Characteristics

The majority of admissions were for women (66.4% female, 33.6% male). Table 3 details patient characteristics.

*Table 3: Patient Characteristics*

| Factor | Admissions | Proportion of total admissions (%) |
|---|---|---|
| Age-sex | | |
| Age 0-44, female | 215 | 1.1 |
| Age 0-44, male | 468 | 2.5 |
| Age 45-64, female | 1068 | 5.7 |
| Age 45-64, male | 792 | 4.2 |
| Age 65-84, female | 6186 | 32.8 |
| Age 65-84, male | 3424 | 18.1 |
| Age 85+, female | 5063 | 26.8 |
| Age 85+, male | 1672 | 8.9 |
| Deprivation | | |
| Quintile 1 (least deprived) | 4796 | 25.4 |
| Quintile 2 | 4467 | 23.6 |
| Quintile 3 | 4090 | 21.7 |
| Quintile 4 | 3143 | 16.6 |
| Quintile 5 (most deprived) | 2300 | 12.2 |
| Quintile 6 (not known) | 92 | 0.5 |
| Dementia | 2634 | 13.9 |
| Alcohol misuse | 625 | 3.3 |
| Arrhythmias | 4130 | 21.9 |
| Heart failure | 1445 | 7.7 |
| COPD | 3337 | 17.7 |
| Deficiency anaemia | 493 | 2.6 |
| Depression | 1231 | 6.5 |
| Diabetes mellitus | 2877 | 15.2 |
| Fluid disorders | 1396 | 7.4 |
| Hypertension | 9208 | 48.8 |
| Hypothyroidism | 1804 | 9.6 |
| Liver disease | 229 | 1.2 |
| Cancer with metastasis | 251 | 1.3 |
| Obesity | 784 | 4.2 |

For peer review only



For peer review only - http://bmjopen.bmj.com/site/about/guidelines.xhtml

| | | |
|---|---|---|
| Other neurological conditions | 1204 | 6.4 |
| Paraplegia | 180 | 1 |
| Pulmonary circulatory disorders | 204 | 1.1 |
| PVD | 520 | 2.8 |
| Renal disease | 2189 | 11.6 |
| Rheumatological disorders | 1619 | 8.6 |
| Solid tumours | 650 | 3.4 |
| Valvular disease | 1091 | 5.8 |

<u>Operative rate and main procedures performed</u>

Given that the coding advice only came into force in March 2015, we now restrict the analysis to the 11,019 discharges from April 2015 onwards, again with M96.6 as the primary diagnosis.

Primary open reduction [W201 + W192 + W202 + W191 + W198] was the commonest recorded surgical technique (21.6%); revision of the prosthesis [W373 + W383 + W384 + W374 + W403 + W393 + W404] was performed in 10.4% (the commonest procedures are listed in Supplementary Table 1).

The overall operative rates were 74.7% in 2015/2016, 72.1% in 2016/17 and 74.5% in 2017/2018. For the elective admissions, 83.5% had a procedure performed; for the emergency admissions, 71.8% had a procedure performed, with an overall proportion of 73.0%.

Table 4 gives the operative rate by patient characteristic and the logistic regression results (See Supplementary Table 2 for operative rates by age, gender, and method of admission). Patients of both sexes aged greater than 84 years were significantly less likely to have an operation than the youngest age group. Surgery was also less likely with increasing socio-economic deprivation, Chronic Obstructive Pulmonary Disease (COPD), and neurological disorders. Surgery was more likely in the presence of recorded fluid and electrolyte disturbance, hypertension, and obesity (OR 1.78, 95% CI 1.46-2.17).

*Table 4: Operative rate by patient characteristics and multivariable logistic regression results*

| Factor | No operation | Operation | Proportion with operation (%) | Adjusted OR for operation | P value |
|---|---|---|---|---|---|
| Age 00-44, female | 52 | 163 | 75.8 | 1.23 (0.83 to 1.81) | 0.299 |
| Age 00-44, male | 97 | 371 | 79.3 | 1 | |
| Age 45-64, female | 203 | 865 | 81.0 | 1.29 (0.91 to 1.83) | 0.158 |
| Age 45-64, male | 152 | 640 | 80.8 | 1.27 (0.88 to 1.82) | 0.206 |
| Age 65-84, female | 1576 | 4610 | 74.5 | 0.86 (0.62 to 1.19) | 0.371 |
| Age 65-84, male | 822 | 2602 | 76.0 | 0.97 (0.69 to 1.34) | 0.830 |
| Age 85+, female | 1674 | 3389 | 66.9 | 0.6 (0.44 to 0.83) | 0.002 |
| Age 85+, male | 524 | 1148 | 68.7 | 0.67 (0.48 to 0.94) | 0.019 |
| Quintile 1 (least deprived) | 1267 | 3529 | 73.6 | 1 | |



For peer review only - http://bmjopen.bmj.com/site/about/guidelines.xhtml

| | | | | | |
|---|---|---|---|---|---|
| Quintile 2 | 1183 | 3284 | 73.5 | 1.00 (0.91 to 1.10) | 0.998 |
| Quintile 3 | 1082 | 3008 | 73.5 | 0.98 (0.89 to 1.08) | 0.742 |
| Quintile 4 | 882 | 2261 | 71.9 | 0.89 (0.80 to 0.98) | 0.025 |
| Quintile 5 (most deprived) | 655 | 1645 | 71.5 | 0.84 (0.75 to 0.95) | 0.004 |
| Quintile 6 (not known) | 31 | 61 | 66.3 | 0.64 (0.41 to 1.01) | 0.053 |
| Alcohol misuse | 151 | 474 | 75.8 | 0.93 (0.76 to 1.14) | 0.484 |
| Arrhythmias | 1208 | 2922 | 70.8 | 0.96 (0.89 to 1.05) | 0.385 |
| COPD | 879 | 2458 | 73.7 | 0.84 (0.74 to 0.96) | 0.008 |
| Cancer with metastasis | 65 | 186 | 74.1 | 1.02 (0.93 to 1.11) | 0.681 |
| Deficiency anaemia | 150 | 343 | 69.6 | 0.89 (0.73 to 1.08) | 0.229 |
| Dementia | 910 | 1724 | 65.5 | 0.88 (0.77 to 1.00) | 0.055 |
| Depression | 319 | 912 | 74.1 | 1.02 (0.89 to 1.16) | 0.830 |
| Diabetes mellitus | 777 | 2100 | 73.0 | 0.98 (0.89 to 1.07) | 0.613 |
| Fluid disorders | 340 | 1056 | 75.6 | 1.31 (1.15 to 1.49) | <0.001 |
| Heart failure | 466 | 979 | 67.8 | 1.13 (1.06 to 1.21) | 0.0004 |
| Hypertension | 2424 | 6784 | 73.7 | 1.07 (0.95 to 1.19) | 0.264 |
| Hypothyroidism | 483 | 1321 | 73.2 | 0.98 (0.71 to 1.35) | 0.907 |
| Liver disease | 56 | 173 | 75.5 | 1.03 (0.75 to 1.42) | 0.868 |
| Obesity | 125 | 659 | 84.1 | 1.78 (1.46 to 2.17) | <0.001 |
| Other neurological conditions | 362 | 842 | 69.9 | 0.80 (0.70 to 0.91) | 0.001 |
| Peripheral vascular disease | 143 | 377 | 72.5 | 0.87 (0.63 to 1.21) | 0.401 |
| Paraplegia | 54 | 126 | 70.0 | 1.28 (0.92 to 1.79) | 0.145 |
| Pulmonary circulatory disorders | 49 | 155 | 76.0 | 1.00 (0.82 to 1.23) | 0.974 |
| Renal disease | 637 | 1552 | 70.9 | 0.98 (0.88 to 1.09) | 0.695 |
| Rheumatological disorders | 455 | 1164 | 71.9 | 0.90 (0.80 to 1.01) | 0.065 |
| Solid tumours | 182 | 468 | 72.0 | 0.96 (0.79 to 1.17) | 0.699 |
| Valvular disease | 297 | 794 | 72.8 | 1.11 (0.96 to 1.28) | 0.161 |

<ins>Length of Stay</ins>

Median acute hospital LOS was 14 nights (IQR 7-26), and median total hospital LOS was 17 nights (IQR 8-33: Supplementary Table 3). Acute hospital LOS increased with increasing age and was higher for older ages for both sexes and for operative than non-operative management at each age group (p<0.001; Figures 2 and 3 and Supplementary Tables 3, 4 and 5).

<Figure 2 here>

<Figure 3 here>

In multivariable logistic regression, increasing age was significantly associated with a longer length of stay. A wide variety of co-morbidities were also associated with a longer LOS, but with a smaller effect than that of age (See Table 5).



For peer review only - http://bmjopen.bmj.com/site/about/guidelines.xhtml

*Table 5: Factors associated with a long LOS (≥26 nights)*

| Factor | OR (95% CI) | P value |
|---|---|---|
| Age 00-44, female | 0.66 (0.21 to 2.12) | 0.489 |
| Age 00-44, male | 1 | |
| Age 45-64, female | 4.41 (1.77 to 10.95) | 0.001 |
| Age 45-64, male | 3.97 (1.58 to 9.95) | 0.003 |
| Age 65-84, female | 11.01 (4.52 to 26.86) | <0.001 |
| Age 65-84, male | 9.95 (4.07 to 24.32) | <0.001 |
| Age 85+, female | 17.93 (7.35 to 43.74) | <0.001 |
| Age 85+, male | 17.00 (6.93 to 41.66) | <0.001 |
| Quintile 1 (least deprived) | 1 | |
| Quintile 2 | 1.03 (0.93 to 1.13) | 0.603 |
| Quintile 3 | 1.02 (0.92 to 1.13) | 0.689 |
| Quintile 4 | 1.09 (0.98 to 1.22) | 0.115 |
| Quintile 5 (most deprived) | 1.15 (1.02 to 1.30) | 0.027 |
| Quintile 6 (not known) | 0.88 (0.50 to 1.53) | 0.642 |
| Alcohol misuse | 1.14 (0.92 to 1.42) | 0.221 |
| Arrhythmias | 1.27 (1.17 to 1.38) | <0.001 |
| COPD | 1.32 (1.17 to 1.50) | <0.001 |
| Cancer with metastasis | 1.10 (1.00 to 1.20) | 0.039 |
| Deficiency anaemia | 1.44 (1.18 to 1.74) | 0.003 |
| Dementia | 1.43 (1.26 to 1.63) | <0.001 |
| Depression | 1.10 (0.96 to 1.27) | 0.187 |
| Diabetes mellitus | 1.19 (1.09 to 1.31) | 0.0002 |
| Fluid disorders | 2.29 (2.04 to 2.56) | <0.001 |
| Heart failure | 1.03 (0.96 to 1.10) | 0.493 |
| Hypertension | 1.00 (0.89 to 1.12) | 0.949 |
| Hypothyroidism | 2.08 (1.54 to 2.81) | <0.001 |
| Liver disease | 0.83 (0.60 to 1.15) | 0.270 |
| Obesity | 1.25 (1.05 to 1.48) | 0.012 |
| Other neurological conditions | 1.47 (1.28 to 1.69) | <0.001 |
| Peripheral vascular disease | 2.12 (1.53 to 2.92) | <0.001 |
| Paraplegia | 1.73 (1.29 to 2.32) | 0.0003 |
| Pulmonary circulatory disorders | 1.05 (0.86 to 1.28) | 0.652 |
| Renal disease | 1.20 (1.08 to 1.33) | 0.0004 |
| Rheumatological disorders | 1.17 (1.04 to 1.32) | 0.012 |
| Solid tumours | 1.68 (1.39 to 2.04) | <0.001 |
| Valvular disease | 1.30 (1.13 to 1.49) | 0.0002 |

For peer review only

Final destination on discharge

89.3% of identified patients were admitted from their usual residence and 74.9% went back there afterwards: 8.9% went to a care home; 5.3% were discharged to a temporary residence; 4.3% died in hospital and 3.6% went to another hospital but for which no HES record could be linked.

The total 30-day mortality rate was 3.3% for the period April 1 2015 to June 30 2018 (deaths via linkage to the national deaths registry were available up to dates including July 31 2018).

Figure 4 shows Kaplan-Meier survival curves for non-operative versus operative cases. See Supplementary Figures 1 & 2 for Kaplan-Meier survival curves by sex and age range respectively.

<Figure 4 here>

**Discussion**

This study sought to establish the burden of periprosthetic fractures on the healthcare system in the UK and identify any factors associated with differences in treatment or outcome. The number of patients admitted to hospital in England with periprosthetic fractures is currently around 450 per month. The overall number of periprosthetic fractures as coded by M96.6 is increasing by approximately 13% each year. Periprosthetic fractures are associated with a relatively prolonged length of stay and increased dependency upon discharge with 25% unable to return directly to their normal residence. 73% of people admitted with a periprosthetic fracture received an operation. Non-operative management was more common in those with co-morbidities, increasing age, and COPD. Mortality was low, at 4.3% for in-hospital and 3.3% for total 30-day mortality; for proximal femoral fractures, 30-day mortality from the latest NHFD report is higher, at 6.1%[3].

There are limitations to the data, and we have tried not to over-interpret the data we have. We cannot be certain how complete and accurate the M96.6 coding is. The study relies on accurate data recorded by clinicians and then coded by clinical coders in hospitals. The primary diagnosis and procedure fields in administrative data are known to have high accuracy (>95%)[10], though secondary diagnoses are subject to some under-recording. There will be variation between hospitals as to when the coding advice was implemented, which is why we estimated the trend in two ways. We have attempted to correct for this by analysing ‘early adopters’ separately from the complete cohort. Reassuringly, the conclusions are broadly the same in terms of absolute numbers and relative rate of rise. We did not attempt to distinguish between the sites of periprosthetic fractures, and some of these fractures will have been following arthroplasty of other joints, and fracture fixation, particularly neck of femur fracture.[11] M96.6 is not associated with an anatomical code to specify the bone and or joint involved. It is possible to attempt to infer the joint involved by any subsequent operation codes (M96.6 itself has no anatomical modifier), but this method is not guaranteed to be accurate and does not provide any information regarding the site of injury of those who were treated non-operatively (approximately 27% of all cases). Nonetheless, despite these limitations, we are confident that the increasing trend amongst older people with joint implants accurately reflects the everyday clinical situation.

HES data only provide in-hospital mortality, but we were able to use the established linkage to the national death registry for deaths up to the end of July 2018. More importantly, there are no direct data on functional outcomes.[12] Such additional data would be invaluable to allow clinicians and patients to discuss the most appropriate management.

This is the first study utilising the specific clinical code M96.6. This was introduced in March 2015, and although Figure 1 shows a dramatic increase when the coding advice was introduced, we are confident that there is a significant increasing underlying trend in patients


For peer review only - http://bmjopen.bmj.com/site/about/guidelines.xhtml

with periprosthetic fractures. There have been few studies that have examined the periprosthetic patient population from a multidisciplinary perspective. There are no studies examining risk assessment for these patients with the exception of a small retrospective analysis, 39 patients with distal femoral fractures from Cleveland Ohio.[13]

Our data are in line with previous studies of complication rates which have demonstrated periprosthetic fracture rates of 0.1-18% for hip, 0.3-5.5% for knee, and 0.5-3% for shoulder arthroplasties.[14]

The number of peri-prosthetic fractures is likely to increase, given the ageing population, increased life expectancy and increasing numbers of people with joint replacements. How these individuals are managed is one of the issues that needs to be addressed in order to provide optimum care. The likely treatment of these injuries varies depending on the location involved and the stability of the implant.

The extensive surgery involved in the operative treatment options for these individuals mean that these patients have to be able to withstand a more significant surgical insult than individuals undergoing primary joint arthroplasty or those being treated for neck of femur fractures. There are also implications for the use of hospital resources with longer operation times, expensive implants, and the availability of revision arthroplasty specialists in addition to the higher cost of post-operative care, including high dependency care.[15]

In-hospital mortality is significant, at around 5%, and the rate of new discharge to a care home of around 15% suggests an increase in dependency of this patient group – this representing those who whilst admitted from their usual residence were discharged to a different location.

For patients presenting with hip fracture, surgical management is almost always viewed as gold standard care for pain relief and to restore mobility where possible. Early surgery is important to allow the patient to sit up thereby reducing risk of respiratory problems, malnutrition and difficulty in toileting. This may be different in peri-prosthetic fractures, particularly those around the knee as patients may be able to tolerate sitting up and pain may be controlled by immobilising the fracture in a cast or brace.

Some patients with frailty do not have the physiological reserve to survive major surgery and this will influence decisions between non-operative management, fixation, and major revision surgery.

For all patients with frailty, either cognitive or physical, the decision regarding surgery should be made with the individual and the multidisciplinary team and in some cases may be viewed as a palliative procedure to manage pain.

Notwithstanding the limitations of our analysis, periprosthetic fractures are likely to create an increasing burden to patients, clinicians and the wider health and social care services. At current rates, the average UK trust will admit around one patient with a periprosthetic fracture each week. If the current trend of a 13% annual increase continues, this will rise to around 2 patients per week in 5- or 6-years' time, equating to around 700 extra bed-days per trust per year, alongside an increase in required operating theatre capacity. These patients are often complex, both surgically and medically, and hospitals may wish to consider whether a specialist local or regional service is provided to ensure optimal decision making and


For peer review only - http://bmjopen.bmj.com/site/about/guidelines.xhtml

management. The costs associated with treating periprosthetic fractures often outstrips reimbursement from funders and so any such service will need to be designed in conjunction with funders to ensure it is financially viable.[16]

Data collection and accurate reporting are going to be needed to identify all of the cases, and an add-on to the NJR is required for complete data capture. If a comparison with proximal femoral fracture is made, the NHS is probably where it was with hip fracture care around 15 years ago, when the care of periprosthetic fractures is concerned. The great improvement in hip fracture care ran in parallel with the emergence of "orthogeriatrics" as a definite entity. Periprosthetic fractures will need the same level of multidisciplinary input that is adequately funded and using all the experience gained with the hip fracture population.

Will individual units be able to cope with demand or will there be regional specialisation? The resources needed will not be limited to orthopaedic surgery, where there will need to be enough adequately trained arthroplasty revision surgeons, but will include perioperative medicine, orthogeriatric input, high dependency care, nursing, physiotherapy and care home provision. Perhaps, dedicated units that deal with hip fractures and periprosthetic fractures will be the model of care for the future, with expertise and training opportunities concentrated in purpose-built units.

The coding change made in 2015 to make the NHS ICD-10 compliant has made the identification of periprosthetic fractures easier. There already appears to be an increase of 15% per year in these cases. This requires planning for future service provision at local and national level. This may be facilitated by prospective data collection using a system similar to the National Joint Registry or National Hip Fracture Database.

**Data Sharing**

Due to information governance rules applicable to HES, no data are available for sharing. HES data is available directly from NHS Digital. Please see: https://digital.nhs.uk/data-and-information/data-tools-and-services/data-services/hospital-episode-statistics/users-uses-and-access-to-hospital-episode-statistics#access-to-data

**Author Contributions**

AB devised the study, extracted and analysed the data, wrote the manuscript, reviewed the manuscript.
RG devised the study, reviewed the data, wrote the manuscript, reviewed the manuscript.
SMW reviewed the data, wrote the manuscript, reviewed the manuscript.
HWJ reviewed the data, wrote the manuscript, reviewed the manuscript.
PA reviewed the data, wrote the manuscript, reviewed the manuscript.
IM reviewed the data, wrote the manuscript, reviewed the manuscript.
EAHC devised the study, reviewed the data, reviewed the manuscript.
HW reviewed the data, wrote the manuscript, reviewed the manuscript.
BMD devised the study, reviewed the data, wrote the manuscript, reviewed the manuscript, and guarantees the paper.

**Acknowledgements**

We would like to thank Ketan Patel for his assistance regarding the use of the code M96.6.

For peer review only

**Funding**
AB and PA work at the Dr Foster Unit, an academic unit in the Department of Primary Care and Public Health, Imperial College London. The unit receives research funding from Dr Foster Intelligence, an independent health service research organization (a wholly owned subsidiary of Telstra). The Dr Foster Unit at Imperial is affiliated with the National Institute of Health Research (NIHR) Imperial Patient Safety Translational Research Centre. The NIHR Imperial Patient Safety Translational Centre is a partnership between the Imperial College Healthcare NHS Trust and Imperial College London. The Department of Primary Care and Public Health at Imperial College London is grateful for support from the NW London NIHR Collaboration for Leadership in Applied Health Research & Care (CLAHRC) and the Imperial NIHR Biomedical Research Centre.

BMD is funded by an NIHR Clinical Lecturer position (CL-2016-14-009).

Funders did not have any input to study conception, design, analysis or manuscript writing.

**Competing Interest Statement**
AB reports grants from Dr Foster, grants from Medtronic, outside the submitted work
RG has nothing to disclose.
HWJ has received payment in past for educational and consultation services to DePuy Synthes and Stryker. Institutional grants received from DePuy Synthes for International Surgeon visitations. Trustee of The John Charnley Trust.
EAHC has nothing to disclose.
PA has nothing to disclose.
BMD is funded by a National Institute of Health Research Clinical Lectureship appointment.
HW has nothing to disclose.
IM has nothing to disclose.
SW has nothing to disclose.

**Disclaimer**
This publication presents independent research funded by the National Institute for Health Research (NIHR). The views expressed are those of the author(s) and not necessarily those of the NHS, the NIHR or the Department of Health and Social Care.

**References**
1. Learmonth I D, Young C, Rorabeck C. The operation of the century; total hip replacement. Lancet 2007;**307**:1508–19.
2. National Joint Registry 16th Report. Available from URL: https://reports.njrcentre.org.uk/Portals/0/PDFdownloads/NJR%2016th%20Annual%20Report%202019.pdf (accessed 20/12/2019)
3. National Hip Fracture Database Annual Report 2019. Available from URL: https://www.nhfd.co.uk/files/2019ReportFiles/NHFD_2019_Annual_Report.pdf (accessed 20/12/2019)
4. Office for National Statistics 2015. Available from URL: https://www.ons.gov.uk/peoplepopulationandcommunity/birthsdeathsandmarriages/lifeexpectancies/bulletins/lifeexpectancyatbirthandatage65bylocalareasinenglandandwales/2015-11-04 (accessed 09/08/2018)
5. Clegg A, Young J, Iliffe S, Rikkert MO, Rockwood K. Frailty in elderly people. Lancet 2013;**381**:752–762.


For peer review only - http://bmjopen.bmj.com/site/about/guidelines.xhtml

6. Clinical Classifications Service – Health & Social Care Information Centre. Coding Clinic Version 4.1. March 2015.
7. NHS Digital. Hospital Episode Statistics. Available from URL: https://digital.nhs.uk/data-and-information/data-tools-and-services/data-services/hospital-episode-statistics (accessed Oct 11 2018)
8. International Classification of Diseases ICD-10. Available from URL: http://apps.who.int/classifications/icd10/browse/2010/en (accessed 10/08/2018)
9. Carstairs V, Morris R. Deprivation and Health in Scotland. Aberdeen University Press, 1991.
10. Burns EM, Rigby E, Mamidanna R, Bottle A, Aylin P, Ziprin P, Faiz OD. Systematic review of discharge coding accuracy. J Public Health (Oxf) 2012;**34(1)**:138–48.
11. Leal J, Gray AM, Prieto-Alhambra D, Arden NK, Cooper C, Jarvaid MK, Judge A. Impact of hip fracture on hospital care costs: a population-based study. Osteoporosis Int 2016;**27**:549–558.
12. Moppett I. Hip fractures: are we asking the right questions? Age Ageing 2018;**47(5)**:633–4.
13. Reich MS, Duong M, Breslin MA, Nguyen MP, Vallier HA. Perioperative Considerations when treating isolated periprosthetic distal femur fractures. The Iowa Orthopedic Journal 2017;**37**:41–45.
14. Capone A, Congia S, Civinini R, Marongiu G. Periprosthetic fractures: epidemiology and current treatment. Clin Cases Miner Bone Metab. 2017;**14(2)**:189–96.
15. Griffiths EJ, Cash DJW, Kalra S, Hopgood PJ. Time to surgery and 30-day morbidity and mortality of periprosthetic hip fractures. Injury. 2013 Dec 1;44(12):1949–52.
16. Jones AR, Williams T, Paringe V, White SP. The economic impact of surgically treated peri-prosthetic hip fractures on a university teaching hospital in Wales 7.5-year study. Injury 2016;47(2):428–31.

I, the Submitting Author has the right to grant and does grant on behalf of all authors of the Work (as defined in the below author licence), an exclusive licence and/or a non-exclusive licence for contributions from authors who are: i) UK Crown employees; ii) where BMJ has agreed a CC-BY licence shall apply, and/or iii) in accordance with the terms applicable for US Federal Government officers or employees acting as part of their official duties; on a worldwide, perpetual, irrevocable, royalty-free basis to BMJ Publishing Group Ltd ("BMJ") its licensees and where the relevant Journal is co-owned by BMJ to the co-owners of the Journal, to publish the Work in BMJ Open and any other BMJ products and to exploit all rights, as set out in our licence.

The Submitting Author accepts and understands that any supply made under these terms is made by BMJ to the Submitting Author unless you are acting as an employee on behalf of your employer or a postgraduate student of an affiliated institution which is paying any applicable article publishing charge ("APC") for Open Access articles. Where the Submitting Author wishes to make the Work available on an Open Access basis (and intends to pay the relevant APC), the terms of reuse of such Open Access shall be governed by a Creative Commons licence – details of these licences and which Creative Commons licence will apply to this Work are set out in our licence referred to above.


For peer review only - http://bmjopen.bmj.com/site/about/guidelines.xhtml

**Figure legends**

Figure 1. Number of admissions by month from April 2013 to December 2018, with elective and emergency admissions shown separately

Figure 2. Acute hospital length of stay by age and gender

Figure 3. Acute hospital length of stay by operative management

Figure 4. Kaplan Meier Survival Curve by Operative Status

For peer review only

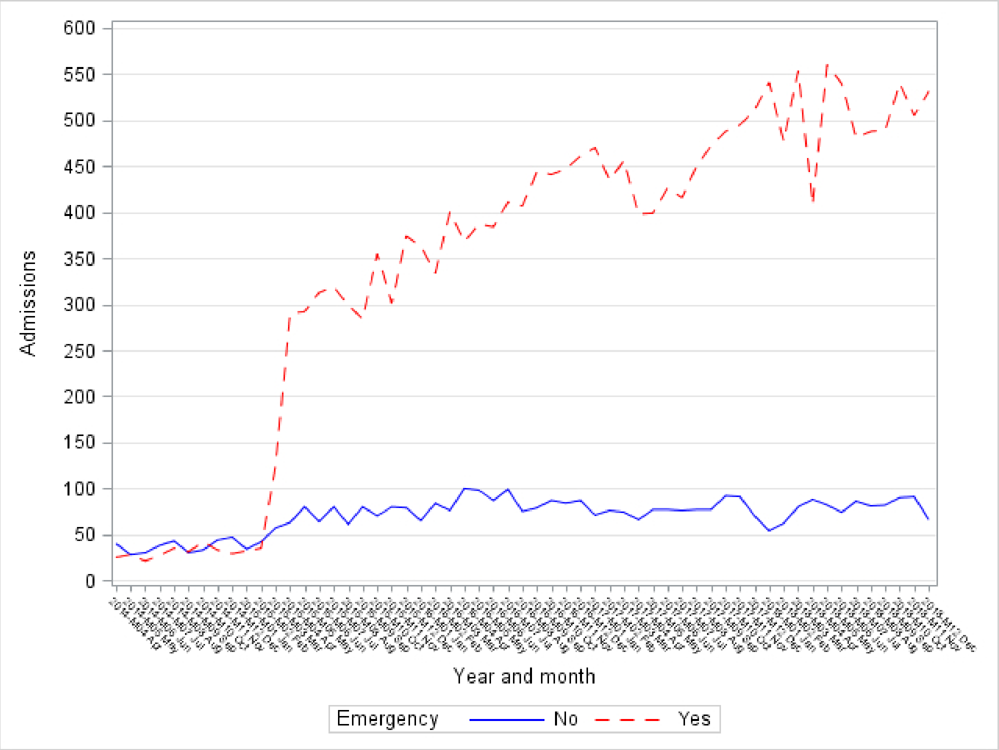


Figure 1. Number of admissions by month from April 2013 to December 2018, with elective and emergency admissions shown separately

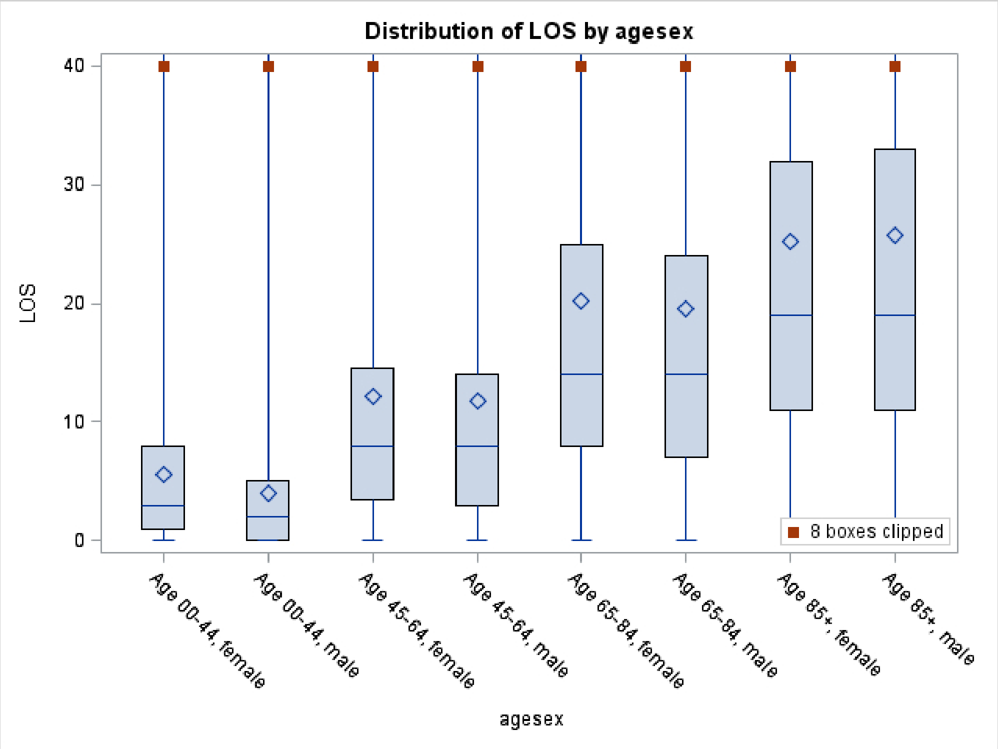


Figure 2. Acute hospital length of stay by age and gender

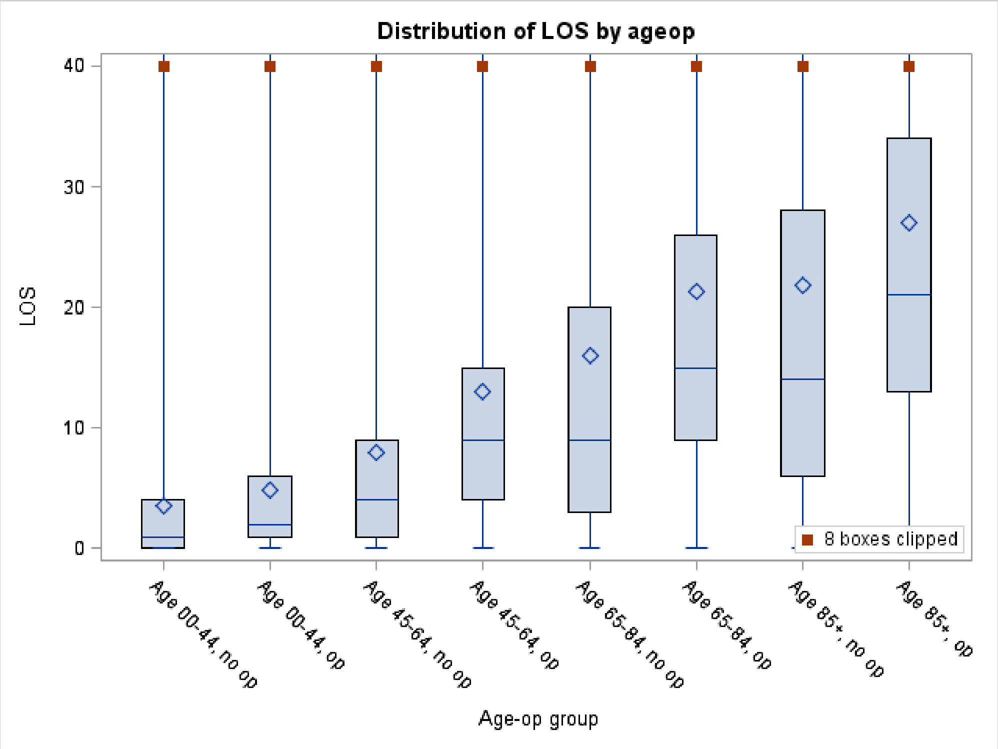


Figure 3. Acute hospital length of stay by operative management

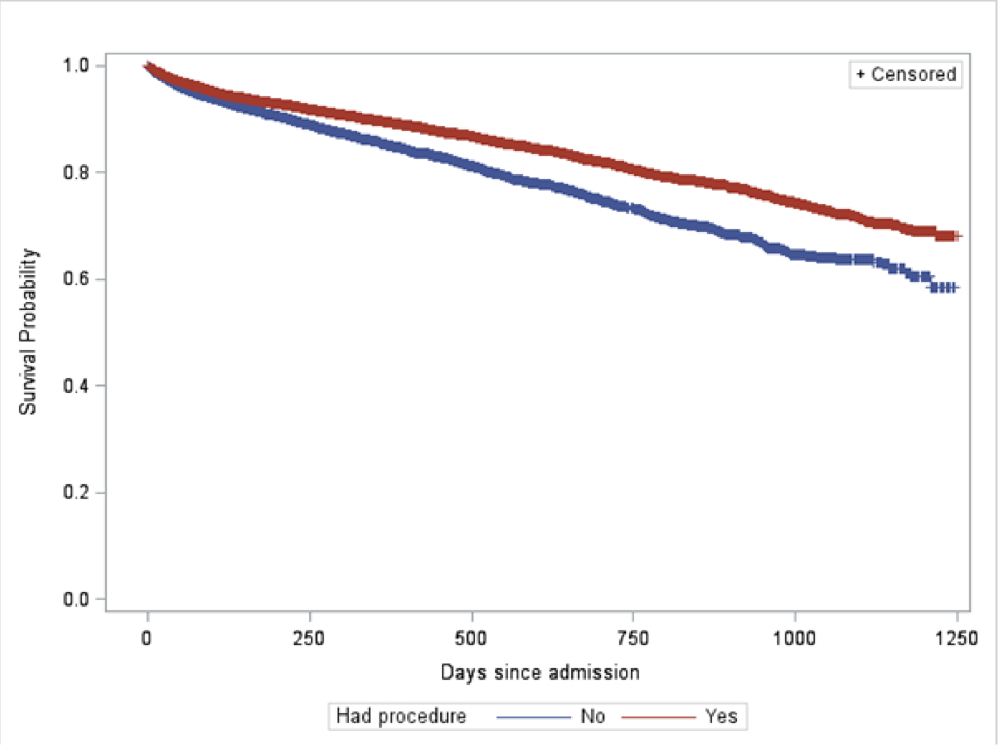


Figure 4. Kaplan Meier Survival Curve by Operative Status

# Supplementary Data - Periprosthetic fractures – the next fragility fracture epidemic? A national observational study

**Supplementary Table 1. The 20 most commonly performed procedures by four-character OPCS code.**

| Op code | Description | Number | Proportion of operations (%) |
|---|---|---|---|
| W201 | Primary open reduction of fracture of long bone and extramedullary fixation using plate NEC | 5004 | 14.8 |
| W283 | Removal of internal fixation from bone NEC | 1728 | 5.1 |
| W202 | Primary open reduction of fracture of long bone and extramedullary fixation using cerclage | 1122 | 3.3 |
| W373 | Revision of total prosthetic replacement of hip joint using cement | 843 | 2.5 |
| W383 | Revision of total prosthetic replacement of hip joint not using cement | 633 | 1.9 |
| W384 | Revision of one component of total prosthetic replacement of hip joint not using cement | 617 | 1.8 |
| W192 | Primary open reduction of fracture of long bone and fixation using rigid nail NEC | 591 | 1.7 |
| W382 | Conversion to total prosthetic replacement of hip joint not using cement | 588 | 1.7 |
| W370 | Conversion from previous cemented total prosthetic replacement of hip joint | 585 | 1.7 |
| W403 | Revision of total prosthetic replacement of knee joint using cement | 582 | 1.7 |
| W374 | Revision of one component of total prosthetic replacement of hip joint using cement | 501 | 1.5 |
| W901 | Aspiration of joint | 495 | 1.5 |
| W281 | Application of internal fixation to bone NEC | 448 | 1.3 |
| W232 | Secondary open reduction of fracture of bone and extramedullary fixation HFQ | 420 | 1.2 |
| W322 | Allograft of bone NEC | 389 | 1.1 |
| W246 | Closed reduction of fracture of bone and fixation using nail or screw | 333 | 1.0 |
| W191 | Primary open reduction of fracture of neck of femur and open fixation using pin and plate | 310 | 0.9 |
| W242 | Closed reduction of fracture of long bone and rigid internal fixation NEC | 300 | 0.9 |
| W198 | Other specified primary open reduction of fracture of bone and intramedullary fixation | 298 | 0.9 |
| W381 | Primary total prosthetic replacement of hip joint not using cement | 258 | 0.8 |
| other | All other main procedures combined | 33922 | 53.7 |

NEC = not elsewhere classified

For peer review only

**Supplementary Table 2. Operative rate according to age, gender and method of admission**

| Factor | Elective admissions with operation | Emergency admissions with operation | % of all elective admissions with operation | % of all emergency admissions with operation |
|---|---|---|---|---|
| Age 0-44, female | 65 | 98 | 95.6 | 66.7 |
| Age 0-44, male | 146 | 225 | 88.5 | 74.3 |
| Age 45-64, female | 229 | 636 | 95.0 | 76.9 |
| Age 45-64, male | 164 | 476 | 92.1 | 77.5 |
| Age 65-84, female | 552 | 4058 | 82.5 | 73.6 |
| Age 65-84, male | 304 | 2298 | 85.4 | 74.9 |
| Age 85+, female | 140 | 3249 | 64.5 | 67.0 |
| Age 85+, male | 56 | 1092 | 62.2 | 69.0 |
| Total | 1656 | 12132 | 83.5 | 71.8 |

**Supplementary Table 3. Length of stay in nights according to major operation status**

| Patient group | LOS type | N | Lower quartile | Median | Mean | Upper quartile |
|---|---|---|---|---|---|---|
| All | Acute stay | 18888 | 7 | 14 | 20.5 | 26 |
| All | Total | 18888 | 8 | 17 | 25.0 | 33 |
| No major operation | Acute stay | 5100 | 4 | 10 | 17.6 | 22 |
| No major operation | Total | 5100 | 4 | 11 | 19.8 | 26 |
| Major operation | Acute stay | 13788 | 9 | 16 | 21.6 | 27 |
| Major operation | Total | 13788 | 10 | 19 | 26.9 | 35 |

"Acute stay" means the stay at the first (acute) hospital; "Total" covers the whole admission, including inter-hospital transfers

**Supplementary Table 4. Length of stay in nights by age and gender**

| Patient group | LOS type | N | Lower quartile | Median | Mean | Upper quartile |
|---|---|---|---|---|---|---|
| Age 0-44, female | Acute stay | 215 | 1 | 3 | 5.6 | 8 |
| | Total | 215 | 1 | 3 | 6.8 | 9 |
| Age 0-44, male | Acute stay | 468 | 0 | 2 | 4.0 | 5 |
| | Total | 468 | 0 | 2 | 4.7 | 5 |
| Age 45-64, female | Acute stay | 1068 | 3.5 | 8 | 12.1 | 14.5 |
| | Total | 1068 | 4 | 9 | 14.6 | 17 |
| Age 45-64, male | Acute stay | 792 | 3 | 8 | 11.8 | 14 |
| | Total | 792 | 3 | 8 | 13.5 | 15 |
| Age 65-84, female | Acute stay | 6186 | 8 | 14 | 20.2 | 25 |
| | Total | 6186 | 9 | 17 | 24.6 | 31 |
| Age 65-84, male | Acute stay | 3424 | 7 | 14 | 19.5 | 24 |
| | Total | 3424 | 8 | 16 | 23.9 | 30 |
| Age 85+, female | Acute stay | 5063 | 11 | 19 | 25.2 | 32 |
| | Total | 5063 | 12 | 23 | 31.0 | 41 |
| Age 85+, male | Acute stay | 1672 | 11 | 19 | 25.7 | 33 |
| | Total | 1672 | 13 | 23 | 31.4 | 43 |

"Acute stay" means the stay at the first (acute) hospital; "Total" covers the whole admission, including inter-hospital transfers



For peer review only - http://bmjopen.bmj.com/site/about/guidelines.xhtml

For peer review only

**Supplementary Table 5. Length of stay in nights by age, gender and operative status**

| Patient group | LOS type | Operated | N | Lower quartile | Median | Mean | Upper quartile |
|---|---|---|---|---|---|---|---|
| Age 0-44, female | Acute stay | No | 52 | 1 | 2 | 4.0 | 5 |
| | | Yes | 52 | 1 | 3 | 6.1 | 8 |
| | Total | No | 163 | 1 | 2 | 4.4 | 6 |
| | | Yes | 163 | 1 | 4 | 7.6 | 10 |
| Age 0-44, male | Acute stay | No | 97 | 0 | 1 | 3.2 | 3 |
| | | Yes | 97 | 1 | 2 | 4.3 | 5 |
| | Total | No | 371 | 0 | 1 | 3.6 | 3 |
| | | Yes | 371 | 1 | 2 | 5.0 | 6 |
| Age 45-64, female | Acute stay | No | 203 | 1 | 4 | 8.3 | 11 |
| | | Yes | 203 | 4 | 9 | 13.0 | 15 |
| | Total | No | 865 | 1 | 4 | 10.2 | 13 |
| | | Yes | 865 | 5 | 10 | 15.6 | 18 |
| Age 45-64, male | Acute stay | No | 152 | 1 | 3 | 7.4 | 7.5 |
| | | Yes | 152 | 4 | 9 | 12.8 | 15 |
| | Total | No | 640 | 1 | 4 | 8.9 | 8 |
| | | Yes | 640 | 4 | 10 | 14.5 | 17 |
| Age 65-84, female | Acute stay | No | 1576 | 3 | 10 | 16.4 | 20 |
| | | Yes | 1576 | 9 | 16 | 21.4 | 26 |
| | Total | No | 4610 | 3 | 10 | 17.9 | 23 |
| | | Yes | 4610 | 11 | 19 | 26.8 | 33 |
| Age 65-84, male | Acute stay | No | 822 | 2 | 8 | 15.1 | 19 |
| | | Yes | 822 | 9 | 15 | 20.9 | 25 |
| | Total | No | 2602 | 2 | 8 | 16.5 | 21 |
| | | Yes | 2602 | 10 | 18 | 26.1 | 32 |
| Age 85+, female | Acute stay | No | 1674 | 6 | 14 | 21.8 | 28 |
| | | Yes | 1674 | 13 | 20 | 26.9 | 34 |
| | Total | No | 3389 | 7 | 16 | 25.3 | 33 |
| | | Yes | 3389 | 15 | 26 | 33.7 | 44 |
| Age 85+, male | Acute stay | No | 524 | 7 | 14 | 21.9 | 27 |
| | | Yes | 524 | 13 | 21 | 27.5 | 34 |
| | Total | No | 1148 | 7 | 16 | 24.9 | 34 |
| | | Yes | 1148 | 16 | 26 | 34.3 | 46 |

"Acute stay" means the stay at the first (acute) hospital; "Total" covers the whole admission, including inter-hospital transfers

For peer review only

**Supplementary Figure 1. Kaplan Meier Survival Curve by sex**

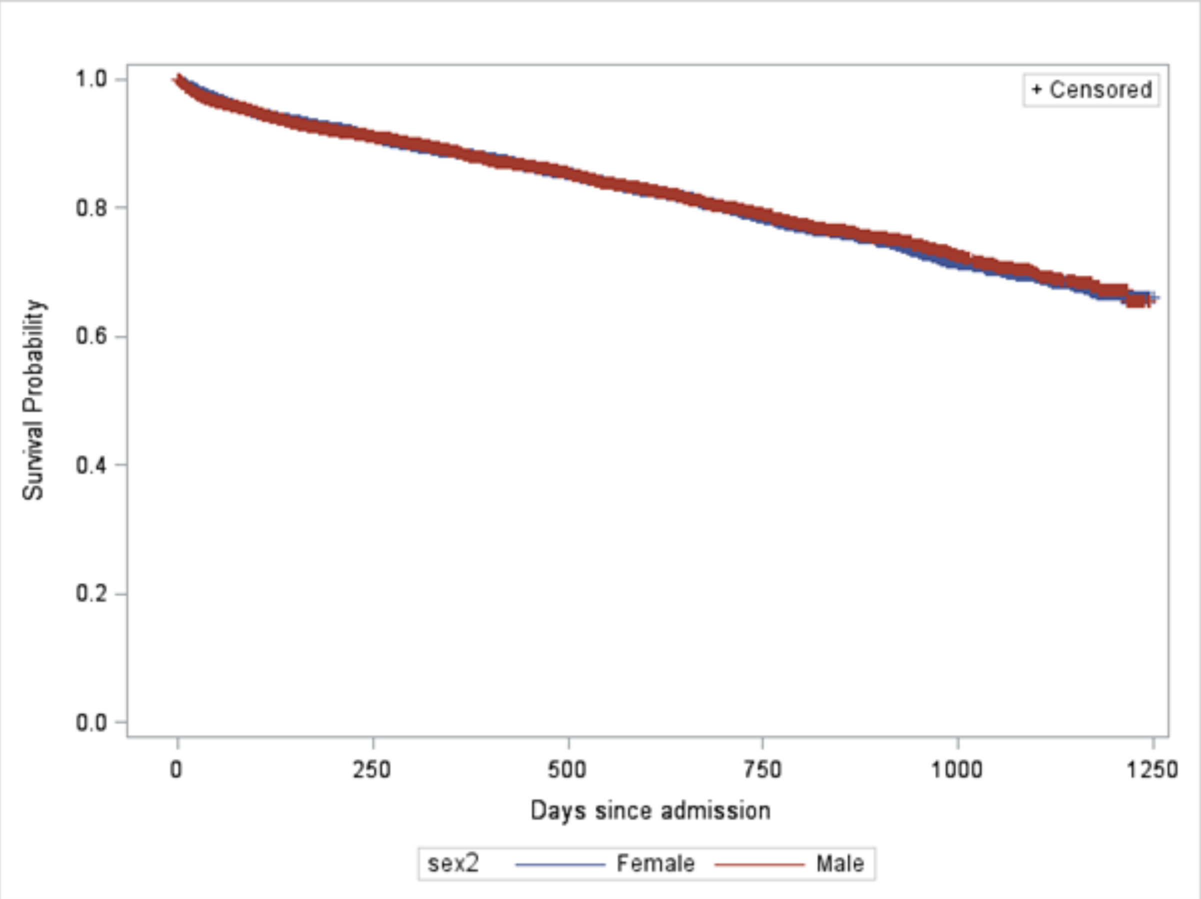


For peer review only

**Supplementary Figure 2. Kaplan Meier Survival Curve by age range**

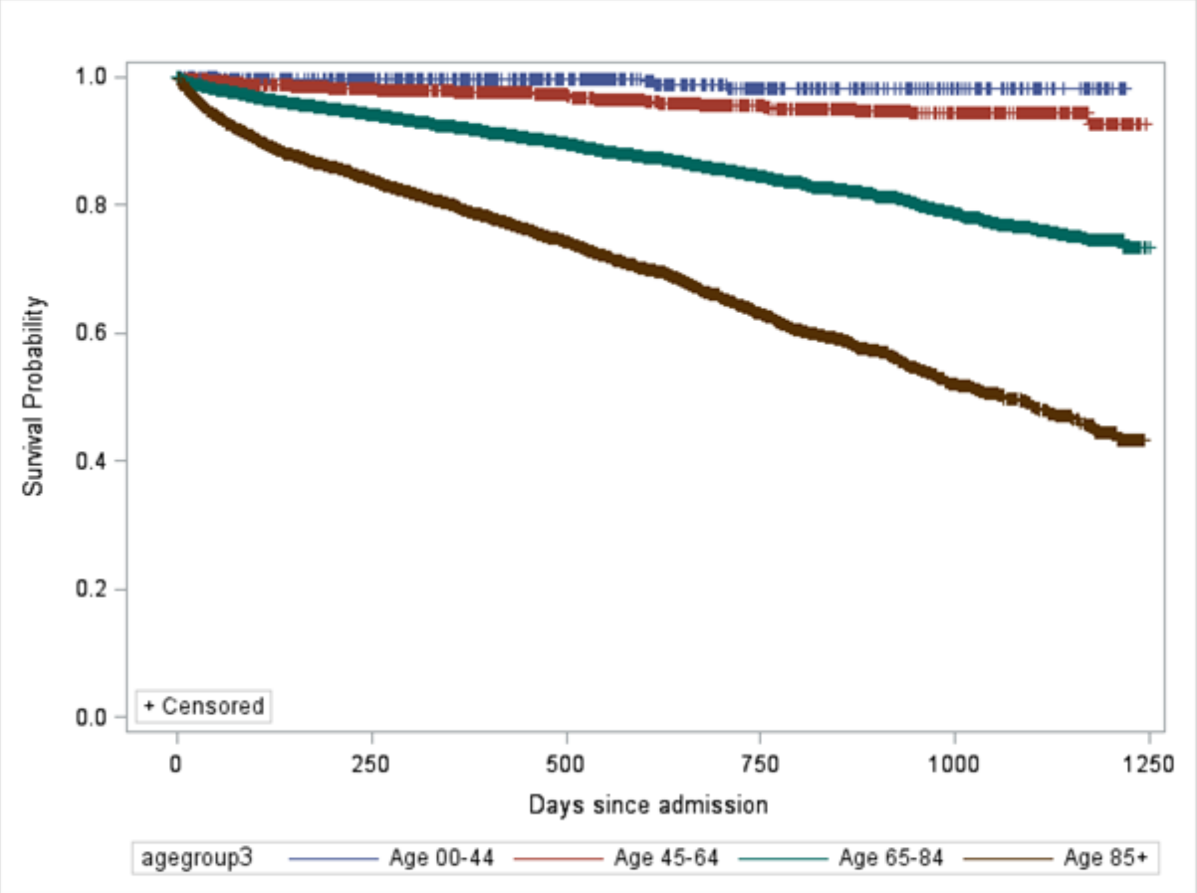


For peer review only

STROBE Statement—checklist of items that should be included in reports of observational studies

| | Item No | Recommendation | Page No |
|---|---|---|---|
| **Title and abstract** | 1 | (*a*) Indicate the study's design with a commonly used term in the title or the abstract | 1 |
| | | (*b*) Provide in the abstract an informative and balanced summary of what was done and what was found | 2 |
| **Introduction** | | | |
| Background/rationale | 2 | Explain the scientific background and rationale for the investigation being reported | 3 |
| Objectives | 3 | State specific objectives, including any prespecified hypotheses | 3 |
| **Methods** | | | |
| Study design | 4 | Present key elements of study design early in the paper | 3 |
| Setting | 5 | Describe the setting, locations, and relevant dates, including periods of recruitment, exposure, follow-up, and data collection | 3-4 |
| Participants | 6 | (*a*) *Cohort study*—Give the eligibility criteria, and the sources and methods of selection of participants. Describe methods of follow-up<br>*Case-control study*—Give the eligibility criteria, and the sources and methods of case ascertainment and control selection. Give the rationale for the choice of cases and controls<br>*Cross-sectional study*—Give the eligibility criteria, and the sources and methods of selection of participants | 3-4 |
| | | (*b*) *Cohort study*—For matched studies, give matching criteria and number of exposed and unexposed<br>*Case-control study*—For matched studies, give matching criteria and the number of controls per case | n/a |
| Variables | 7 | Clearly define all outcomes, exposures, predictors, potential confounders, and effect modifiers. Give diagnostic criteria, if applicable | 4 |
| Data sources/ measurement | 8* | For each variable of interest, give sources of data and details of methods of assessment (measurement). Describe comparability of assessment methods if there is more than one group | 3-4 |
| Bias | 9 | Describe any efforts to address potential sources of bias | |
| Study size | 10 | Explain how the study size was arrived at | 4 |
| Quantitative variables | 11 | Explain how quantitative variables were handled in the analyses. If applicable, describe which groupings were chosen and why | |
| Statistical methods | 12 | (*a*) Describe all statistical methods, including those used to control for confounding | 4 |
| | | (*b*) Describe any methods used to examine subgroups and interactions | 4 |
| | | (*c*) Explain how missing data were addressed | n/a |
| | | (*d*) *Cohort study*—If applicable, explain how loss to follow-up was addressed<br>*Case-control study*—If applicable, explain how matching of cases and controls was addressed<br>*Cross-sectional study*—If applicable, describe analytical methods taking account of sampling strategy | |
| | | (*e*) Describe any sensitivity analyses | 4 |

Continued on next page



For peer review only - http://bmjopen.bmj.com/site/about/guidelines.xhtml

| | | | |
|---|---|---|---|
| **Results** | | | |
| Participants | 13* | (a) Report numbers of individuals at each stage of study—eg numbers potentially eligible, examined for eligibility, confirmed eligible, included in the study, completing follow-up, and analysed | 4 |
| | | (b) Give reasons for non-participation at each stage | n/a |
| | | (c) Consider use of a flow diagram | |
| Descriptive data | 14* | (a) Give characteristics of study participants (eg demographic, clinical, social) and information on exposures and potential confounders | 5 (and table 3) |
| | | (b) Indicate number of participants with missing data for each variable of interest | |
| | | (c) *Cohort study*—Summarise follow-up time (eg, average and total amount) | |
| Outcome data | 15* | *Cohort study*—Report numbers of outcome events or summary measures over time | 5-6 |
| | | *Case-control study*—Report numbers in each exposure category, or summary measures of exposure | |
| | | *Cross-sectional study*—Report numbers of outcome events or summary measures | |
| Main results | 16 | (*a*) Give unadjusted estimates and, if applicable, confounder-adjusted estimates and their precision (eg, 95% confidence interval). Make clear which confounders were adjusted for and why they were included | |
| | | (*b*) Report category boundaries when continuous variables were categorized | Table 4 |
| | | (*c*) If relevant, consider translating estimates of relative risk into absolute risk for a meaningful time period | |
| Other analyses | 17 | Report other analyses done—eg analyses of subgroups and interactions, and sensitivity analyses | 6-8 |
| **Discussion** | | | |
| Key results | 18 | Summarise key results with reference to study objectives | 9-10 |
| Limitations | 19 | Discuss limitations of the study, taking into account sources of potential bias or imprecision. Discuss both direction and magnitude of any potential bias | 11 |
| Interpretation | 20 | Give a cautious overall interpretation of results considering objectives, limitations, multiplicity of analyses, results from similar studies, and other relevant evidence | 11 |
| Generalisability | 21 | Discuss the generalisability (external validity) of the study results | 11-12 |
| **Other information** | | | |
| Funding | 22 | Give the source of funding and the role of the funders for the present study and, if applicable, for the original study on which the present article is based | 12 |

*Give information separately for cases and controls in case-control studies and, if applicable, for exposed and unexposed groups in cohort and cross-sectional studies.

**Note:** An Explanation and Elaboration article discusses each checklist item and gives methodological background and published examples of transparent reporting. The STROBE checklist is best used in conjunction with this article (freely available on the Web sites of PLoS Medicine at http://www.plosmedicine.org/, Annals of Internal Medicine at http://www.annals.org/, and Epidemiology at http://www.epidem.com/). Information on the STROBE Initiative is available at www.strobe-statement.org.


For peer review only - http://bmjopen.bmj.com/site/about/guidelines.xhtml
